# Asynchronous magnetic resonance elastography: Shear wave speed reconstruction using noise correlation of incoherent waves

**DOI:** 10.1002/mrm.29502

**Published:** 2022-10-27

**Authors:** Khoi D. Nguyen, Benjamin P. Bonner, Anna N. Foster, Mehdi Sadighi, Christopher T. Nguyen

**Affiliations:** ^1^ Cardiovascular Innovation Research Center Heart Vascular & Thoracic Institute, Cleveland Clinic Cleveland Ohio USA; ^2^ Cardiovascular Research Center Massachusetts General Hospital Charlestown Massachusetts USA; ^3^ Martinos Center for Biomedical Imaging Massachusetts General Hospital Charlestown Massachusetts USA; ^4^ Department of Diagnostic Radiology Imaging Imaging Institute, Cleveland Clinic Cleveland Ohio USA; ^5^ Department of Cardiovascular & Metabolic Sciences Lerner Research Institute, Cleveland Clinic Cleveland Ohio USA

**Keywords:** elastography, noise correlation, shear wave speed, tissue stiffness

## Abstract

**Purpose:**

The noninvasive measurement of biological tissue elasticity is an evolving technology that enables the robust characterization of soft tissue mechanics for a wide array of biomedical engineering and clinical applications. We propose, design, and implement here a new MRI technique termed asynchronous magnetic resonance elastography (aMRE) that pushes the measurement technology toward a driverless implementation. This technique can be added to clinical MRI scanners without any additional specialized hardware.

**Theory:**

Asynchronous MRE is founded on the theory of diffuse wavefields and noise correlation previously developed in ultrasound to reconstruct shear wave speeds using seemingly incoherent wavefields. Unlike conventional elastography methods that solve an inverse problem, aMRE directly reconstructs a pixel‐wise mapping of wave speed using the spatial–temporal statistics of the measured wavefield.

**Methods:**

Incoherent finger tapping served as the wave‐generating source for all aMRE measurements. Asynchronous MRE was performed on a phantom using a Siemens Prismafit as an experimental validation of the theory. It was further performed on thigh muscles as a proof‐of‐concept implementation of in vivo imaging using a Siemens Skyra scanner.

**Results:**

Numerical and phantom experiments show an accurate reconstruction of wave speeds from seemingly noisy wavefields. The proof‐of‐concept thigh experiments also show that the aMRE protocol can reconstruct a pixel‐wise mapping of wave speeds.

**Conclusion:**

Asynchronous MRE is shown to accurately reconstruct shear wave speeds in phantom experiments and remains at the proof‐of‐concept stage for in vivo imaging. After further validation and improvements, it has the potential to lower both the technical and monetary barriers of entry to measuring tissue elasticity.

## 1 INTRODUCTION

Tissue elastography builds on the physical principle that mechanical waves propagate faster in stiff tissues than in soft tissues and, by noninvasively imaging the speed of propagation, infer tissue elasticity.^1^, ^2^, ^3^, ^4^, ^5^ The clinical utility and diagnostic value of elasticity characterizations has previously been shown in liver, breast, and eye tissues where an abnormally high stiffness is a marker of diseased pathological state.^5^, ^6^, ^7^, ^8^ Furthermore, undergoing active research is the use of tissue elasticity in heart and brain studies to better understand diseases and functions underlaid by the organ's biomechanics.^3^, ^9^, ^10^, ^11^, ^12^, ^13^ The imaging modality differs depending on the tissue of interest and is typically MRI, ultrasound, or optical coherence tomography. In the case of MRI, the technology is known as magnetic resonance elastography (MRE), and we propose, design, and implement in this paper a novel MRE technique that does not require additional hardware.

In conventional implementations,^1^, ^2^, ^3^, ^6^, ^14^, ^15^ MRE tracks the propagation of shear waves externally generated by a commercially available, MR‐compatible driver placed on the skin near the tissue of interest and vibrating at a user‐specified frequency. The driver is akin to an ultrasound transducer that also generates mechanical waves at the skin, and so can also suffer from limited tissue penetration depth, which leads to lower signal quality for deeper tissues. In situations in which conventional MRI may not generate sufficient signal, alternative implementations of MRE that do not rely on an external driver can instead provide the desired information. Furthermore, a driverless MRE implementation would lower both the technical and monetary barriers of entry for incorporating tissue elasticity into the clinical domain because it would in principle be another pulse sequence that does not require any additional specialized hardware.

In this paper, we present a new driverless implementation of MRE based on the theory of noise correlation and ideas developed in ultrasound elastography, where wave‐generating sources internal to the body, such as intrinsic mechanical vibrations from the heart or vocal cords,^9^, ^12^, ^13^, ^16^, ^17^, ^18^ are used instead of an external driver. The tradeoff between temporal and spatial resolutions has therefore far prevented the translation of these ideas to MRI, where the higher imaging quality and relative ease of 3D imaging can provide more comprehensive and accurate measurements of tissue elasticity. We present here a solution that bypasses this tradeoff and which we refer to as asynchronous magnetic resonance elastography (aMRE). Specifically, aMRE combines information from two separate scans, one of high temporal resolution but low spatial resolution and another of high spatial but low temporal resolution, to allow for direct measurement of shear wave speeds. The asynchrony in aMRE references the lack of communication between the MRI machine and the wave‐generating source, which conventional MRE implementations require. The source may be internal or external to the body, but the point of importance is that the exact temporal characteristics of the source need not be known a priori because they will be directly measured. This opens the possibility of wave‐generating sources that do not require additional MR‐compatible hardware, and, in this paper, we use random tapping of a volunteer's fingers on their skin as the source.

The paper proceeds with preliminaries that introduce the theory of elastography based on noise correlation, although we refer to more dedicated works for a deeper dive into the mathematics.^18^, ^19^, ^20^, ^21^ As two independent forms of validation, the paper provides numerical simulations and phantom experiments to test the accuracy of aMRE in measuring wave speeds from diffuse wavefields generated by random finger tappings. Next, we use aMRE and finger tappings in the thigh muscles of volunteers as a proof‐of‐concept implementation for in vivo imaging. Finally, we conclude with a discussion of aMRE's potential in the clinical domain and of its technical limitations.

## 2 THEORY

Elastography based on noise correlation is founded on the idea of diffuse wavefields consisting of random plane waves propagating in all directions with equal intensity and without attenuation. Structured wavefields, for comparison, consist of plane waves propagating along certain directions. In practice, the wavefield is typically an experimentally measured tissue velocity and assumed to be diffuse if multiple wave sources (either external or internal to the body) and multiple wave‐reflecting tissue boundaries lead to an apparently incoherent wavefield.^4^, ^16^ This incoherence has traditionally been treated as ambient noise inside the human body but is treated here as the primary signal of interest whose spatial–temporal statistics are used to infer tissue elasticity.

The wave speed calculation centers on a spatial–temporal correlation function between two nearby points.^18^, ^19^, ^20^ Given a diffuse wavefield ϕ(r,t) as a function of the spatial coordinate r and time t, the correlation function is defined as

(1) $$A\left(r,\delta r,t\right)=\int_{-\infty }^{\infty }\phi \left(r,\mu \right)\phi \left(r+\delta r,\mu +t\right)d\mu$$

for two points separated by a displacement vector δr. If the temporal dynamics of the diffuse wavefield also consists of a single dominant characteristic frequency (ie, the spectrum of ϕ(r,t)) has a single localized peak for all r, then the wave speed c can be approximated in terms of the correlation function at δr=0 and t=0 as

(2) $$\begin{array}{cc} & c^{2}\approx {\left.\frac{\frac{\partial^{2}A}{\partial t^{2}}}{\frac{\partial^{2}A}{\partial r^{2}}}\right|}_{\delta r=0,t=0}. \end{array}$$

We refer to Zemzemi et al^21^ for a more exact relation between wave speed and the correlation function. A physical interpretation of Equation (2) can be made in terms of a spatial correlation length scale λ and a temporal correlation time scale τ. The correlation function A(r,δr,t) peaks at δr=0 and t=0 and decays to zero as either δr or t increases, and, like a Gaussian distribution, the magnitude at the peak divided by its curvature defines the characteristic scales over which the function decays to zero. The decay due to δr defines the spatial correlation length scale λ, while the decay due to t similarly defines the temporal correlation time scale τ such that the ratio of the two yields wave speed. Namely,

(3) $$\begin{array}{cc} & c=\frac{\lambda }{\tau } \end{array}$$

where

(4) $$\begin{array}{cc} & \lambda \approx 2\pi \sqrt{\frac{A}{\frac{\partial^{2}A}{\partial r^{2}}}}\;\text{and}\;\tau \approx 2\pi \sqrt{\frac{A}{\frac{\partial^{2}A}{\partial t^{2}}}}. \end{array}$$

The length scale λ is the dominant wavelength within the diffuse wavefield with a corresponding frequency f=1/τ. Equation (3) can also be directly expressed in terms of the wavefield ϕ(r,t) as

(5) $$\begin{array}{cc} & \;{\left.\frac{\partial^{2}A}{\partial t^{2}}\right|}_{\delta r=0,t=0}=-\int_{-\infty }^{\infty }{\left(\frac{\partial \phi }{\partial t}\right)}^{2}\mathrm{dt}\;\text{and}\\ & {\left.\frac{\partial^{2}A}{\partial r^{2}}\right|}_{\delta r=0,\;t=0}=-\int_{-\infty }^{\infty }{\left(\frac{\partial \phi }{\partial r}\right)}^{2}\mathrm{dt} \end{array}$$

This yields an expression based solely on its derivatives as

(6) $$\begin{array}{cc} & c^{2}\approx \frac{\int_{-\infty }^{\infty }\;{\left(\frac{\partial \phi }{\partial t}\right)}^{2}\mathrm{dt}}{\int_{-\infty }^{\infty }{\left(\frac{\partial \phi }{\partial r}\right)}^{2}\mathrm{dt}}. \end{array}$$

Equation (6) shows that this method of computing wave speed based on noise correlation requires only knowledge of ϕ(r,t) at r and nearby points, and sufficient spatial and temporal resolutions to numerically compute the derivatives.

### 2.1 Numerical simulations

**FIGURE 1:**
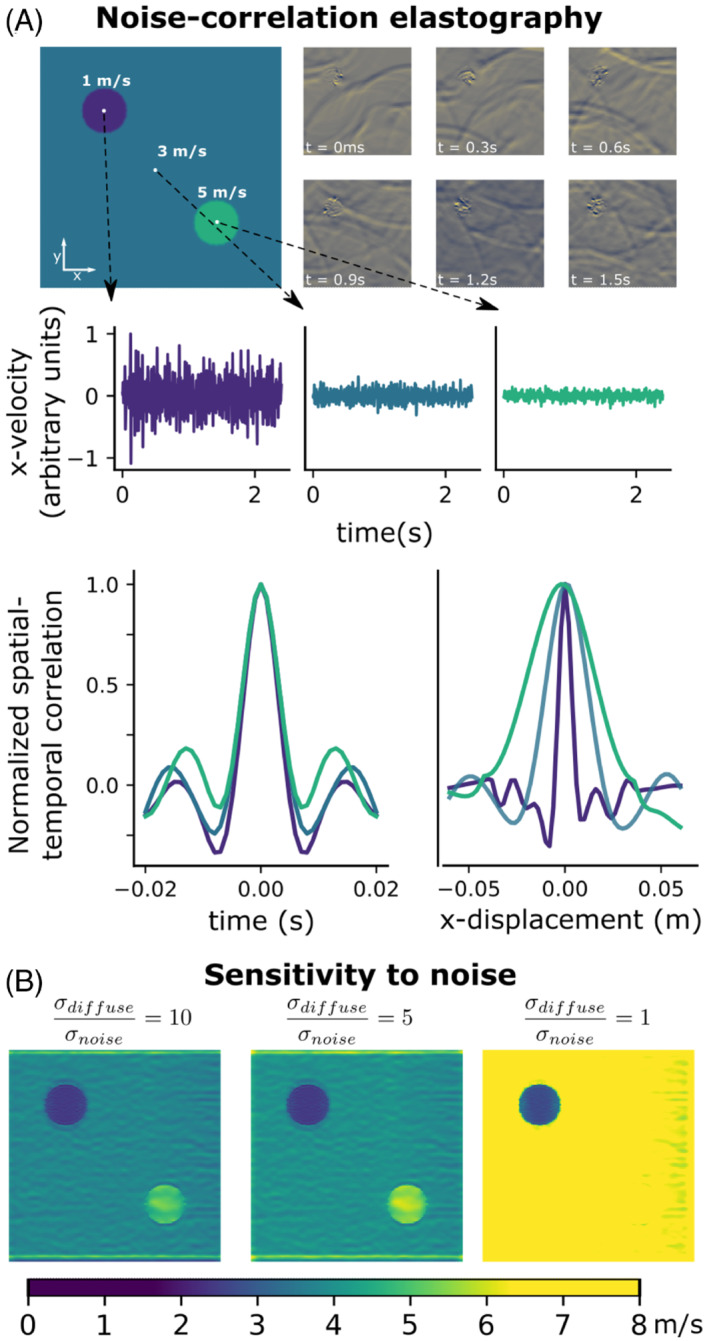
Numerical simulations of wave speed reconstruction using spatial–temporal correlations. A, Diffuse wavefield simulation using k‐wave,^22^ an acoustics library programmed in *MATLAB*, and characteristic decay in space and time of the correlation function for three sample points. The source code and time‐series images are available as Supporting Information. B, The ratio of the decay in space to the decay in time leads to a pixelwise reconstruction of wave speeds. Three reconstructions are shown for different levels of measurement noise in the form of additive white Gaussian noise. Higher noise levels lead to a systematic overestimation of wave speeds

As a validation of theory, we use numerical experiments to demonstrate an accurate computation of wave speeds from a diffuse wavefield simulated in a 2D elastic medium with circular inclusions of varying wave speeds (see Figure 1). The simulation is an implementation of k‐wave,^22^ an acoustics library programmed in *MATLAB* (Natick, MA), and computes longitudinal waves instead of shear waves for computational simplicity and because Equations (2, )–(6) are equally valid for either type of mechanical waves. A diffuse wavefield is generated within a square elastic medium (512 × 512 mm) with reflecting boundary conditions and by a set of five randomly localized point sources. These point sources perturb the elastic medium to generate radially expanding waves that propagate throughout the elastic medium and reflect off the boundaries. After a transitory period, the wavefield is assumed diffuse due to destructive interference of multiple overlapping reflections of the initial point source perturbations. The velocity along the *x*‐axis is then taken to be the measured signal ϕ(r,t) in Equation (6) and then used to directly calculate wave speeds of the elastic medium (3 m/s) and of the two circular inclusions (1 and 5 m/s wave speeds and 40‐mm radius) by numerical approximations of the spatial and time derivatives.

A deeper look into the spatial–temporal correlation functions for representative sample points show that the correlation length scale λ and time scale $\tau =f^{-1}$ can be extracted from apparently incoherent signals. The sample points capture the elastic medium and two circular inclusions and numerically show that the variable wave speeds affect only λ but not τ (see Figure 1A), as the characteristic decay in the correlation function is variable in space but constant in time for the three sample points. More precisely, τ is invariant in space because there is no loss or gain of energy as waves propagate in an elastic medium. Consequently, timescale τ is determined solely by the dynamics of the initial wave‐generating perturbations. The correlation length scale λ necessarily varies in space because wave speed varies in space. For these reasons, τ is a constant and λ will vary with spatial position r such that the reconstructed wave speed image is c(r)=λ(r)/τ.

Furthermore, to evaluate sensitivity to measurement noise, white Gaussian noise is added to the signal ϕ(r,t) at different noise levels before the wave speed reconstruction. In phase‐contras MRI where the desired signal is the phase rather than magnitude, the SD of velocity noise $\sigma_{\text{noise}}$ relates to the SNR of the magnitude image as follows:

(7) $$\begin{array}{cc} & \frac{v_{\mathrm{enc}}}{\sigma_{\text{noise}}}=\frac{\pi }{\sqrt{2}}\mathrm{SNR} \end{array}$$

where $v_{\mathrm{enc}}$ is the velocity‐encoding parameter and SNR is defined as the ratio of expected magnitude signal to the SD of background noise.^23^ For our purposes, we choose $v_{\mathrm{enc}}=\left(\frac{\pi }{\sqrt{2}}\right)\sigma_{\text{diffuse}}$, where $\sigma_{\text{diffuse}}$ is the SD of the zero‐mean diffuse wavefield such that $\sigma_{\text{diffuse}}/\sigma_{\text{noise}}$ is simply equal to SNR. Figure 1B shows the wave speed reconstruction for conservative SNR values ranging from 1 to 10. For comparison, typical SNR values for phase‐contrast imaging range from 10 to 100.^24^, ^25^ The reconstruction closely matches the true values at low noise and starts to overestimate the values as the noise level increases. An ambient background remains even for the lowest noise reconstruction, which we attribute to a finite simulation time and deviations from the diffuse wavefield idealization. The systematic overestimation at high noise is a numerical artifact because evaluating wave speed c in Equation (6) for white Gaussian noise will yield the ratio of spatial to temporal resolutions of the numerical simulation, which must be greater than the highest wave speeds for numerical convergence. In practice, we expect that the wave speed reconstruction at high noise will evaluate the ratio of characteristic length and time scales inherent to the noise and not of the diffuse wavefield.

## 3 METHODS

**FIGURE 2:**
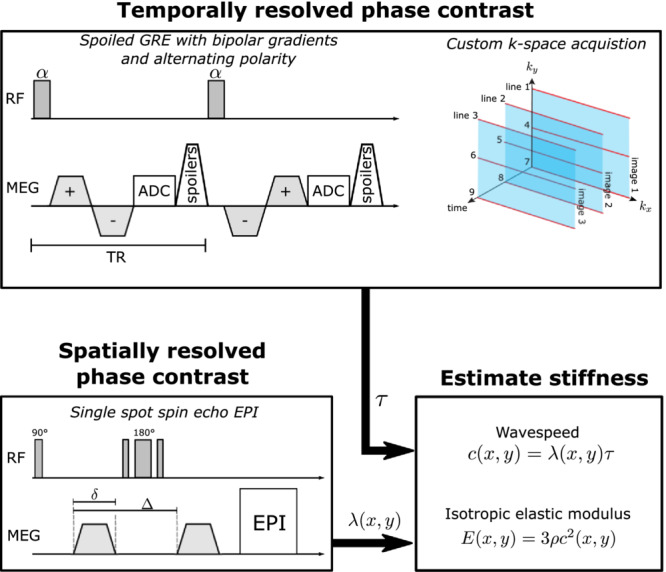
Imaging protocol for asynchronous magnetic resonance elastography (aMRE). Flow chart for the aMRE protocol combines information from two scans designed to separately measure the temporal and spatial properties of a diffuse wavefield. Abbreviations: GRE, gradient echo; MEG, motion‐encoding gradients

Phase‐contrast MRI using motion‐encoding gradients allows for direct measurement of tissue velocity along any spatial direction.^26^, ^27^, ^28^ However, unlike numerical simulations with arbitrarily high temporal and spatial resolutions, phase‐contrast MRI does not offer sufficiently high temporal resolutions without a restrictive decrease in spatial resolution. To mitigate the tradeoff, we use two scans that measure parameters τ and λ of Equation (4) separately. The computation of τ involves only temporal and not spatial derivatives, so it can be measured by a phase‐contrast scan with high temporal but low spatial resolutions. Furthermore, because the time scale is independent of space as illustrated in Figure 1A, taking a pixel‐wise average over the entire region of interest yields a noise‐filtered estimate for the time scale. The computation of λ, on the other hand, involves spatial and not temporal derivatives and can be measured by a scan with high spatial but low temporal resolutions. However, unlike τ, a spatial average is not taken because the variations in shear wave speeds are embedded in λ. The final wave speed image is then generated by dividing λ by constant τ (see Figure 2). Because the wave speed reconstruction combines information from two separate scans, the dynamics of finger tapping or any wave‐generating source must be consistent across the scans and would introduce errors if sufficiently different.

### 3.1 Temporally resolved phase contrast

The pulse sequence designed for the temporally resolved scan involves spoiled gradient echo with bipolar motion‐encoding gradients and alternating polarity. The spoilers are necessary for fast acquisition, and the polarity helps to remove spurious phase contributions. However, unlike standard phase‐contrast imaging, we implement a custom Cartesian k‐space acquisition scheme programmed in Pulseq,^29^ an open‐source library, to further improve temporal resolution by sacrificing spatial coherence. Specifically, each k‐space line is sampled multiple times before moving on to the next k‐space line, and the *i*th image is formed by collecting the *i*th sample of each k‐space line. This custom acquisition scheme is illustrated in the flowchart of Figure 2, where the acquisition goes in numerical order (1 or 9) but the images are formed using lines out of numerical order (ie, 1, 4, 7 for the first image). The result of this custom k‐space acquisition scheme is a wavefield that is spatially coherent along the read‐out direction but not the phase‐encoding direction, because different k‐space lines of the *i*th image are collected far apart in time. The loss of spatial coherence does not affect the calculation of τ, however, because it is a constant independent of space for an elastic material that does not exhibit any frequency dispersion. The temporal resolution here is 2*TR, regardless of the number *N* of k‐space lines.

### 3.2 Spatially resolved phase contrast

The spatially resolved scan is a single‐shot spin‐echo EPI pulse sequence with motion‐encoding gradients. The sequence is a modification of the standard diffusion sequence^30^, ^31^ at ultralow *b*‐values on the order of 10^−3^ s/mm^2^. Specifically, the *b*‐value and delta parameters (δ,Δ) of the diffusion sequence maps to the velocity‐encoding parameter $v_{\mathrm{enc}}$ of phase‐contrast imaging as follows:

(8) $$\begin{array}{cc} & v_{\mathrm{enc}}={\left[\frac{1}{b}\left(\frac{1}{\Delta }-\frac{\delta }{3\Delta^{2}}\right)\right]}^{\frac{1}{2}} \end{array}$$

where the delta parameters are minimized, and *b*‐value is chosen to yield the desired velocity‐encoding parameter. The single‐shot EPI ensures snapshot imaging of the wavefield because the waves propagate faster than can be imaged using multiple RF excitations.

### 3.3 Imaging parameters

**FIGURE 3:**
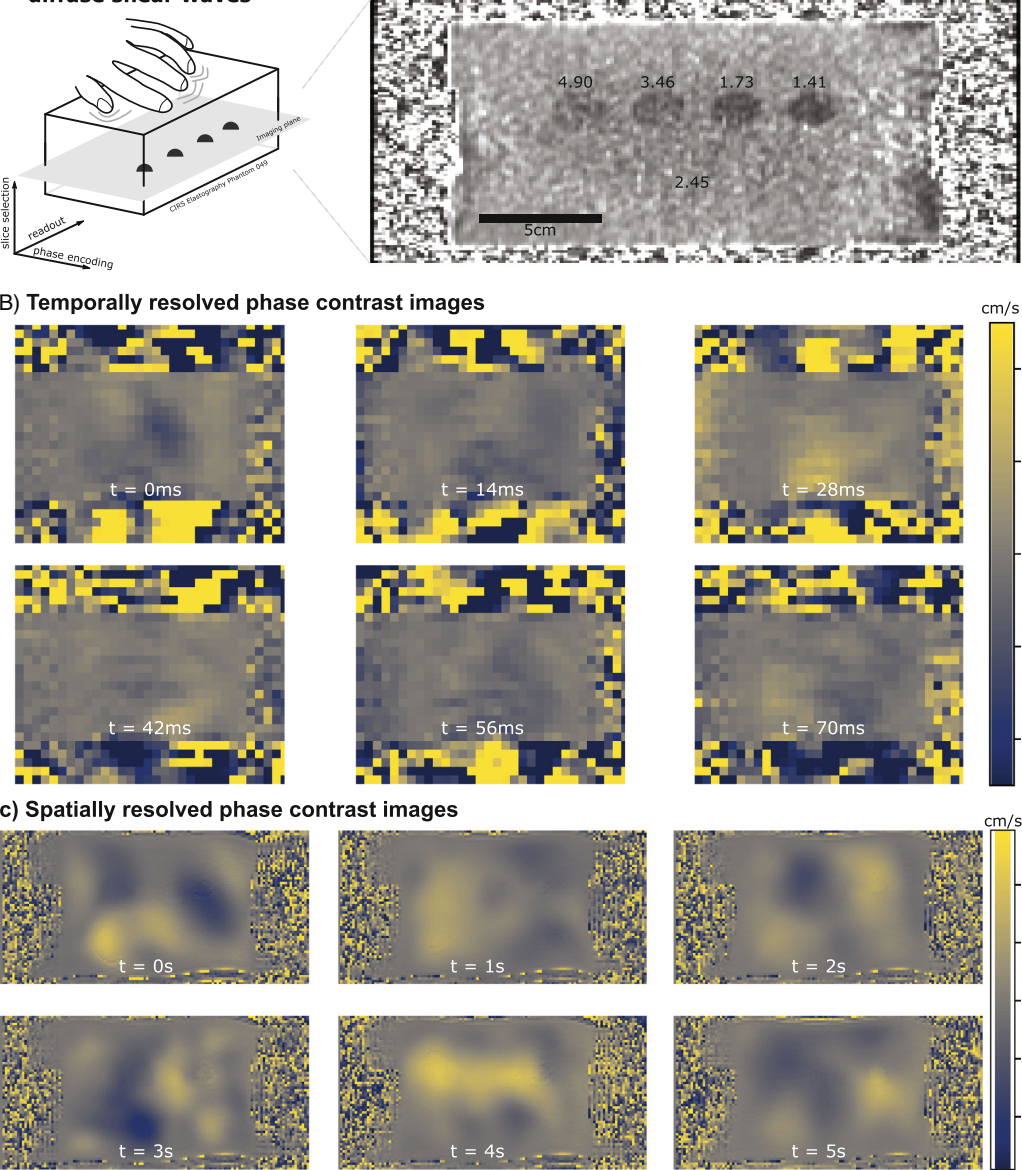
Snapshots for aMRE protocol on an elastography phantom. A, Illustration of the box‐shaped phantom, the imaging plane, the spherical inclusions of varying stiffnesses, and the finger tapping on the top of the box. A T_2_‐weighted image is also shown that overlaps the known wave speeds in units of m/s. B, C, The first few reconstructed images of the temporally resolved and spatially resolved phase‐contrast scans. All velocity measurements were sensitized along the phase‐encoding direction

We implement aMRE on an elastography phantom (CIRS model 049) as an experimental validation of the theory using a Siemens Prismafit 3T and a 64‐channel head coil. A volunteer resided inside the bore with the phantom to randomly finger‐tap on the phantom's surface for the duration of the scans (Figure 3A). The imaging parameters for the temporally resolved scan are $v_{\mathrm{enc}}=15$ cm/s, spin angle α=10°, TR = 7.1 ms, FOV = 220 × 220 mm, in‐plane resolution = 6.8 mm, slice thickness = 10 mm, 400 images, and acquisition time of approximately 2 min. For the spatially resolved scan, $v_{\mathrm{enc}}=15$ cm/s, *b* = 1.4*10^−3^ s/mm^2^, δ=14.8 ms, Δ=25.2 ms, TR = 1000 ms, FOV = 305 × 152.5 mm, in‐plane resolution = 2.38 mm, slice thickness = 5 mm, 640 images, and acquisition time of approximately 11 min.

We also implement aMRE on thigh muscles as a proof of concept for in vivo imaging using a Siemens Skyra 3T and single‐channel extremity coil. Two volunteers were asked to finger tap with both hands either on the front anterior or the sides of their left thigh for the duration of the scans while in a supine position. The volunteers were also instructed to keep their thigh muscles at rest. The parameters for the temporally resolved scan are $v_{\mathrm{enc}}=10\;$ cm/s, spin angle α=10°, TR = 11.3 ms, FOV = 320 × 320 mm, in‐plane resolution = 10 mm, slice thickness = 10 mm, 350 images, and acquisition time of approximately 2 min. For the spatially resolved scan, $v_{\mathrm{enc}}=10$ cm/s, *b* = 4*10^−3^ s/mm^2^, δ=9.8 ms, Δ=20.2 ms, TR = 1000 ms, FOV = 305 × 152.5 mm, in‐plane resolution = 2.38 mm, slice thickness = 5 mm, 320 images, and acquisition time of approximately 5 min.

All protocols were approved by the institutional review board at Massachusetts General Hospital and informed consent was obtained for all volunteers. The volunteers for the in vivo thigh scan are healthy males aged 29 and 55. The total duration of aMRE was approximately 13 min for the phantom scan and 7 min for the in vivo scans. Volunteers did not report any finger fatigue from tapping continuously for both durations. All phase‐contrast scans were sensitized along the phase‐encoding direction. Following image reconstruction, a phase‐unwrapping filter was applied to all images.^32^

### 3.4 Numerical computation of wave speed

Denoting the reconstructed phase signals from the temporally and spatially resolved scans as $\phi_{T}\left(t_{i},x_{j},y_{k}\right)$ and $\;\phi_{s}\left(t_{i},x_{j},y_{k}\right)$, respectively, for discretized time $t_{i}$ and space $\left(x_{j},y_{k}\right)$, we numerically approximate wave speed using discretized versions of Equations (3, )–(5). The time derivative is numerically computed using a forward difference to approximate $\partial \phi_{T}/\partial t\;$ and the spatial derivative using a centered difference to approximate $\partial \phi_{S}/\partial r$, where the spatial coordinate r is taken to be along the phase direction $y_{k}$. Specifically,

(9) $$\begin{array}{cc} & \frac{\partial \phi_{T}}{\partial t}\left(t_{i},x_{j},y_{k}\right)\approx \frac{\phi_{T}\left(t_{i+1},x_{j},y_{k}\right)-\phi_{T}\left(t_{i},x_{j},y_{k}\right)}{\Delta t} \end{array}$$

(10) $$\begin{array}{cc} & \;\frac{\partial \phi_{S}}{\partial r}\left(t_{i},x_{j},y_{k}\right)\approx \frac{\phi_{S}\left(t_{i},x_{j},y_{k+1}\right)-\phi_{S}\left(t_{i},x_{j},y_{k-1}\right)}{2\Delta y} \end{array}$$

where resolutions (Δt,Δy) equal 14.2 ms and 2.38 mm for the phantom experiment and 22.6 ms and 2.38 mm for the in vivo experiment. The wavelength λ and timescale τ are then computed as follows:

(11) $$\begin{array}{cc} \lambda \left(x_{j},y_{k}\right) & =2\pi \sqrt{\frac{\sum_{i}\phi_{S}^{2}}{\sum_{i}{\left(\frac{\partial \phi_{S}}{\partial r}\right)}^{2}}\;}\;\text{and}\\ \tau & =2\pi E\left[\sqrt{\frac{\sum_{i}\phi_{T}^{2}}{\sum_{i}{\left(\frac{\partial \phi_{T}}{\partial t}\right)}^{2}}\;}\right] \end{array}$$

where the summation over i numerically integrates the phase signals in time and E[·] is the spatial average over the entire tissue or phantom being imaged. The total integration times are 640 s and 2.84 s for the phantom experiment and 350 s and 3.62 s for the in vivo experiment. The estimated wave speed is then computed as $c\left(x_{j},y_{k}\right)=\lambda \left(x_{j},y_{k}\right)/\tau$.

## 4 RESULTS

### 4.1 Phantom experiments

The elastography phantom (CIRS model 049) is a tissue‐mimicking device with elastic moduli representative of soft tissues, and we show here that aMRE with finger tapping can accurately reconstruct the phantom's known elasticities. The phantom is a rectangular box with spherical inclusions of elastic moduli varying from 6 to 75 kPa, which map to shear wave speeds in the range of 1.4–5.0 m/s according to the relation

(12) $$\begin{array}{cc} & c=\sqrt{\frac{E}{3\rho }} \end{array}$$

for elastic moduli E and density $\rho \approx 10^{3}$ kg/m^3^ (see Figure 3A). The first few images of the temporally and spatially resolved scans are shown in Figure 3B,C with the entire time series available as Supporting Information. By design, the waves in the temporally resolved scan are not distinct but the fast framerate (*dt* = 14 ms) allows us to analyze their dynamics. The scan resulted in a correlation time scale τ = 53 ms, which we speculate to be the average contact time of the volunteer's finger taps. The waves in the spatially resolved scans, on the other hand, are far more distinct but are imaged sufficiently far apart in time that it is not possible to observe their propagation.

**FIGURE 4:**
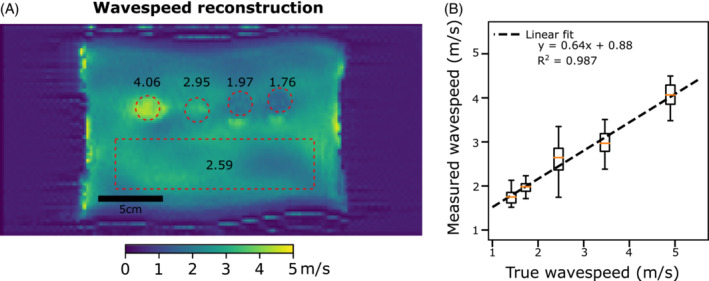
Shear wave speed reconstruction of elastography phantom. A, The reconstructed wave speeds sampled at τ=53 ms or f=19 Hz with manually drawn regions of interest for the background and each of the four spherical inclusions. The averaged wave speed is overlaid on each region. B, Plotting true wave speeds against measured wave speed using pixels from the regions of interest show a strong correlation and that aMRE can reconstruct wave speeds. The fitted line is a linear regression using the average of each region of interest and its true wave speed

Combining the two scans yields the wave speed reconstruction in Figure 4A. A geometric distortion is visible throughout the entire image and most apparent along the top and bottom boundaries of the rectangular box. This geometric distortion is due to EPI of the spatially resolved scan and to be improved upon in further iterations of aMRE. Drawing regions of interests for the four spherical balls and the background and then correlating the pixels inside the regions show a clear correlation ($R^{2}=0.987$) between measured and true wave speeds.

### 4.2 Proof‐of‐concept in vivo imaging

**FIGURE 5:**
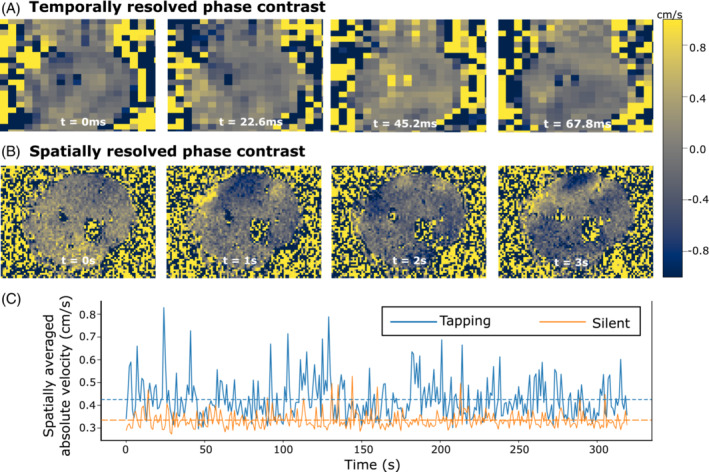
Proof of concept in vivo thigh scan using aMRE and finger tapping. A, B, The first few reconstructed images of the temporally resolved and spatially resolved phase‐contrast scans for a healthy volunteer (male, 29 year old). The entire time series is available as Supporting Information. C, Averaging the absolute velocity over the thigh cross‐section shows that, over time, the phase signal from finger tapping is consistently higher than without tapping over time. The dashed horizontal lines further average the signals in time to quantitatively show the difference between tapping and without tapping

The same protocol on a healthy volunteer (male, 29 years old) shows that aMRE and finger tapping can reconstruct the underlying tissue wave speeds of thigh muscles at rest. Here, we imaged a transverse cross‐section of the volunteer's left thigh while he laid in a relaxed supine position and randomly tapped with all 10 fingers on the front, anterior side of the thigh. Snapshots of the temporally and spatially resolved scans are also shown in Figure 5A,B with the entire time series for all scans available as Supporting Information. Figure 5C shows a sanity check demonstrating that finger tapping generates higher phase signals than without tapping by plotting the absolute tissue velocities averaged over the entire thigh and measured from the spatially resolved scan.

**FIGURE 6:**
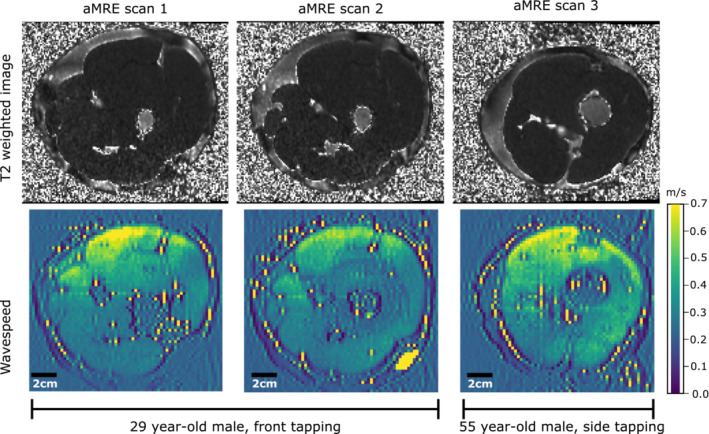
Wave speed reconstruction of in vivo thigh scans. Three aMRE protocols were implemented on 2 healthy male volunteers. The wave speeds were sampled at τ≈95 ms or f≈11 Hz for the three scans. Snapshots of the first scan on the left are displayed in Figure 5

Although finger tapping is not a well‐controlled task, we demonstrate that the wave speed reconstruction is reproducible in Figure 6 by comparing two separate aMRE scans on the same volunteer. Both scans show roughly similar wave speed magnitudes in different muscle groups and a consistently higher wave speed for the anterior thigh muscles than the posterior muscles. We also show a third aMRE scan of a second volunteer (male, 55 years old) who tapped with both hands on the sides of his left thigh (Figure 6). The higher wave speeds of anterior muscles are also apparent in this side tapping scan, suggesting that the higher wave speeds of the anterior muscles are not a consequence of proximity to finger tapping. The correlation time scales for these three scans ranged from 93 ms to 97 ms.

## 5 DISCUSSION

A driverless implementation of conventional MRE methods is a natural progression of the current elastography technology and will further push forward the application of tissue elasticity in patient care, because it does not require additional specialized hardware. We showed here a solution to the relatively slow framerates typical of MRI, which allow for a direct translation of ideas previously developed in ultrasound elastography and which we refer to as asynchronous MRE. It combines information from a temporally resolved scan (∼2 min) and a spatially resolved scan (∼5–11 min) to reconstruct shear wave speeds. The wave‐generating sources used in this study are random finger tappings by a volunteer on their skin near the region of interest rather than by a commercial driver. Although finger tapping is comparatively far less precisely controlled than a driver, we showed, using numerical and phantom experiments, that wave speeds can be accurately reconstructed by imaging the noisy and incoherent waves generated by tapping. The in vivo thigh wave speed reconstructions take this further and show a proof‐of‐concept application of aMRE in volunteers. Our work thus puts forth aMRE as a potentially new MRI tool available to both clinicians and researchers interested in tissue elasticity. Importantly, aMRE is not necessarily a direct replacement of conventional MRE or ultrasound elastography techniques. However, it offers an imaging solution for when the specialized driver is not available or for deep tissues that are otherwise difficult to image with ultrasound, which have typical penetration depths of several centimeters,^10^, ^33^ whereas the aMRE scans shown here easily image objects that are approximately 10–20 cm in size.

Unlike elastography phantoms, tissues are neither purely elastic nor isotropic, and any form of elastography will depend on both the direction and frequency of measurement.^4^, ^10^, ^33^, ^34^, ^35^ The thigh muscles are a topical example. Their stiffnesses will change by several fold, depending on whether the measurement is made along or perpendicular to their fiber orientations.^33^ They also have viscous effects that slowly dissipate elastic energy over time.^35^, ^36^ How the viscous dissipation affects muscle function or correlates with the muscle's physiological state are open questions, but its effects here are to modulate the estimated wave speed in a frequency‐dependent manner such that low frequencies lead to lower estimates and high frequencies approach an asymptotic value.^4^, ^10^, ^36^ The measured wave speeds in the three thigh aMRE scans are about 0.5 m/s (Figure 6), but other elastography measurements estimate that the quadriceps are closer to 2 m/s,^33^, ^37^ an approximate 4‐fold difference. This is in part because the finger tapping probes the viscoelastic properties on a time scale of about 95 ms or 11 Hz in the low‐frequency regime, whereas other elastography methods probe the properties in the high‐frequency regime. Furthermore, the correlation timescale τ computed from the temporally resolved scan can vary in space because of viscous dissipation. Finally, the thigh muscles themselves are likely functioning as waveguides, because wavelengths on the order of 0.5 m/s*95 ms = 4.8 cm are on the same size scale as the thigh muscles. Finite boundary effects come into play and can lead to Lamb waves rather than shear waves that assume an infinite medium.^5^ For both viscoelastic and finite boundary effects, the 4‐fold difference can be minimized in future iterations of aMRE by exploring wave‐generating sources acting faster than finger tapping, such mechanical vibrations arising from the cardiac cycle or vocal cords.

There are also technical limitations to aMRE that can be iterated and improved upon. First, the resolution of the temporally resolved scan can be increased further. Although the resolution used in the experiments here are fast relative to typical MRI scans, a 5–10‐fold improvement is necessary to image shear waves on the same frequencies used in conventional MRE and ultrasound elastography methods.^1^, ^2^, ^3^, ^10^, ^33^ Currently, the resolution is 14 ms in the phantom scans and 22 ms in the in vivo scans, whereas cardiac MRE typically uses frequencies exceeding 100 Hz that require a resolution of 5 ms or better to image the waves. This improvement may arise from further optimization of parameters in the current bipolar gradient pulse sequence (Figure 2) or exploring other fast phase‐contract techniques such as balanced SSFP.^27^ Second, geometric distortions arising from the single‐shot EPI imaging of the spatially resolved scan appear to be the biggest factor limiting spatial resolution. This distortion can be minimized in future iterations by a more careful selection of imaging parameters, distortion correction techniques, and post‐reconstruction algorithms designed to filter out the distortions. Finally, an overall improvement in phase‐contrast sensitivity for both temporally and spatially resolved scans will allow for imaging of lower amplitude shear waves without sacrificing SNR. This opens the possibility of using passive physiological noise or other external vibration sources as the wave‐generating source that act faster than finger tapping and importantly do not require voluntary effort from the patient. Showing that these sources work in conjunction with aMRE would greatly improve the ease of measuring tissue elasticity in patients.

## 6 CONCLUSIONS

Tissue elasticity can be a marker for diseased physiological states and is a crucial component of all biomechanical models used to simulate and design therapeutic devices. We proposed, designed, and implemented aMRE as an imaging solution to noninvasively measure the stiffness of the tissue of interest by mapping the tissue elasticity at every pixel of the image. It is a paradigm shift away from current elastography technology that relies on specialized hardware to generate mechanical shear waves and prior knowledge of an input mechanical shear wave. We showed using numerical simulations, phantom experiments, and in vivo scans that aMRE can reconstruct the seemingly noisy and incoherent waves generated by a volunteer's random finger tapping into an accurate spatial mapping of shear wave speeds. This technique remains at the proof‐of‐concept stage for in vivo imaging and requires further validation and improvements, but has the near‐term potential to lower both the technical and monetary barriers of entry to incorporating tissue elasticity into new biomedical research studies seeking to noninvasively characterize tissue elasticity, and eventually clinical studies seeking to diagnose patients.

## AUTHOR CONTRIBUTIONS

*Study concept*: KDN and CTN. *Methodology*: KDN, BPB, ANF, MS, and CTN. *Investigation*: KDN, BPB, ANF, MS, and CTN. *Visualization*: KDN. *Supervision*: CTN. *Manuscript draft*: KDN. *Writing, reviewing, and editing*: KDN, BPB, ANF, MS, and CTN.

## FUNDING INFORMATION

The National Institutes of Health (R01 HL151704, R01 HL159010, and R01 HL135242) and a fellowship from the Sarnoff Cardiovascular Research Foundation (to BPB)

## CONFLICT OF INTEREST

The authors declare that they have no competing interests.

## Supporting information


**DATA S1** *MATLAB* code used to generate the numerical simulations of Figure 1

**VIDEO S1** Video showing the complete time series of the k‐wave implementation of a diffuse wavefield illustrated in Figure 1A

**VIDEO S2** Video showing the complete time series of the temporally resolved scan of the phantom illustrated in Figure 3B

**VIDEO S3** Video showing the complete time series of the spatially resolved scan of the phantom illustrated in Figure 3C

**VIDEO S4** Video showing the complete time series of the temporally resolved scan of the thigh asynchronous magnetic resonance elastography (aMRE) scan shown in Figures 5 and 6 (labeled as “aMRE scan 1”)

**VIDEO S5** Video showing the complete time series of the spatially resolved scan of the thigh aMRE scan shown in Figures 5 and 6 (labeled as “aMRE scan 1”)

**VIDEO S6** Video showing the complete time series of the temporally resolved scan of the thigh aMRE scan in Figure 6 (labeled as “aMRE scan 2”)

**VIDEO S7** Video showing the complete time series of the spatially resolved scan of the thigh aMRE scan in Figure 6 (labeled as “aMRE scan 2”)

**VIDEO S8** Video showing the complete time series of the temporally resolved scan of the thigh aMRE scan in Figure 6 (labeled as “aMRE scan 3”)

**VIDEO S9** Video showing the complete time series of the spatially resolved scan of the thigh aMRE scan in Figure 6 (labeled as “aMRE scan 3”)

## Data Availability

All data needed to evaluate the conclusions in the paper are present in the paper and/or the Supporting Information.
