# Supplementary material for: Asynchronous magnetic resonance elastography: Shear wave speed reconstruction using noise correlation of incoherent waves
Source: Magn Reson Med. 2022 Oct 27;89(3):990–1001. doi: 10.1002/mrm.29502 (PMC9792433; doi:10.1002/mrm.29502)
Supplement: Supplementary file 1 — DATA S1 MATLAB code used to generate the numerical simulations of Figure 1 [file MRM-89-990-s002.zip › k-Wave/k-wave_user_manual_1.1.pdf]

k-Wave

A MATLAB toolbox for the time domain simulation of acoustic wave fields

User Manual

Manual Version 1.1 (August 27, 2016), Toolbox Release 1.1
Authored by Bradley Treeby, Ben Cox, and Jiri Jaros

Contents

1 Introduction **1**
- 1.1 Overview . . . 1
- 1.2 History and Contributors . . . 1
- 1.3 What's in this Manual . . . 2
- 1.4 Installation . . . 2
- 1.5 License . . . 3
- 1.6 Alternative Software . . . 4

2 Numerical Model **5**
- 2.1 Governing Equations . . . 5
- 2.2 Acoustic Source Terms . . . 7
- 2.3 Overview of the k-space pseudospectral method . . . 8
- 2.4 Discrete k-space Equations . . . 12
- 2.5 Modelling Power Law Acoustic Absorption . . . 15
- 2.6 Perfectly Matched Layer . . . 16
- 2.7 Accuracy, Stability and the CFL Number . . . 18
- 2.8 Smoothing and the Band-Limited Interpolant . . . 23

3 First-Order Simulation Functions **26**
- 3.1 Overview . . . 26
- 3.2 Defining the Computational Grid . . . 27
- 3.3 Defining the Acoustic Medium . . . 31
- 3.4 Defining the Acoustic Source Terms . . . 33
- 3.5 Defining the Sensor . . . 36
- 3.6 Optional Input Parameters . . . 40
- 3.7 Using a Diagnostic Ultrasound Transducer as a Source or Sensor . . . 41
- 3.8 Improving Performance using the 'DataCast' Option . . . 48

4 Using optimised CPU and GPU Codes **51**
- 4.1 Overview . . . 51
- 4.2 Running Simulations using the Optimised Codes . . . 52
- 4.3 Reloading the Output Data into MATLAB . . . 56
- 4.4 Running the Code using a Bash Script . . . 57
- 4.5 Running the Code from MATLAB . . . 57
- 4.6 Format of the HDF5 Input and Output files . . . 58
- 4.7 Compiling the CPU/GPU Source Code in Linux . . . 61

4.8 Compiling the CPU/GPU Source Code in Windows 64
4.9 Performance and Memory Usage 64

Appendix A List of Optional Input Parameters 68

Appendix B Format of the C++ HDF5 Files 71

Appendix C Performance Evaluation of the CPU and GPU code 79

Chapter 1

Introduction

1.1 Overview

k-Wave is an open source, third party, MATLAB toolbox designed for the time-domain simulation of propagating acoustic waves in 1D, 2D, or 3D. The toolbox has a wide range of functionality, but at its heart is an advanced numerical model that can account for both linear and nonlinear wave propagation, an arbitrary distribution of heterogeneous material parameters, and power law acoustic absorption [1, 2]. The interface to the simulation functions has been designed to be both flexible and user friendly, while the computational engine has been optimised for speed and accuracy. The functions are called using MATLAB scripts with user-defined input parameters, so some familiarity with the MATLAB environment is necessary to get started. However, the toolbox now includes more than 50 worked examples and is also supported by an online forum (`http://www.k-wave.org/forum`). k-Wave is still under active development, and its functionality is still evolving. This process is helped immensely by feedback from you, the user community. So if something is missing, doesn't work the way it should, or fails to do what you'd hoped, please get in touch.

1.2 History and Contributors

The k-Wave toolbox was originally developed within the Photoacoustic Imaging Group at University College London (UCL). The first beta version, released in July 2009, focussed primarily on forward and inverse initial value problems for the simulation and reconstruction of photoacoustic[1] wave fields in lossless media [1]. Subsequent releases of the toolbox have extended this functionality to include time varying pressure and velocity sources, acoustic absorption, nonlinearity, elastic materials, and models for ultrasound transducers. The overall development of the toolbox has been driven by Bradley Treeby and Ben Cox (UCL), while the C++ version of `kspaceFirstOrder3D` is developed by Jiri Jaros (Brno University of Technology). A considerable number of other users, collaborators,

[1]Photoacoustic tomography is a biomedical imaging modality based on the thermoelastic generation of ultrasound waves using pulsed laser light [3].

and students have also contributed to this project, both directly (through code development) and indirectly (through suggestions, usage feedback, and bug reports). A sincere thanks goes to the user community for continuing to support the toolbox.

1.3 What's in this Manual

This manual includes a general introduction to the governing equations and numerical methods used in the main simulation functions in k-Wave for fluid media. It also provides a basic overview of the software architecture and a number of canonical examples. The content is divided into three main sections, which can be read largely independently. Section 2 describes the underlying governing equations and numerical methods, Sec. 3 describes how to use the main simulation functions in MATLAB, and Sec. 4 describes how to install and use the C++ code. More details on the elastic code can be found in [4].

The manual is intended to accompany the extensive html documentation that is also provided with the toolbox. After installation, the html documentation can be accessed from the MATLAB help browser by selecting "k-Wave Toolbox" from the contents page. In versions of MATLAB prior to 2012b, the help browser is opened by clicking on the blue question mark icon on the menu bar. In MATLAB 2012b (and later), the documentation is accessed by selecting "Help" from the ribbon bar, and then clicking on "Supplemental Software". In MATLAB 2015a (and later), the documentation is accessed by selecting "Help" from the ribbon bar, and then selecting "k-Wave Toolbox" from under the "Supplemental Software" heading. This additional documentation provides detailed information on how to use individual functions as well as more than 50 worked examples.

1.4 Installation

The k-Wave toolbox is installed by adding the root k-Wave folder to the MATLAB path. This can be done using the "Set Path" dialog box which is accessed by typing `>> pathtool` at the MATLAB command line.[2] This dialog box can also be accessed using the dropdown menus "File → Set Path" if using MATLAB 2012a and earlier, or the the "Set Path" button on the ribbon bar if using MATLAB 2012b and later. Once the dialog box is open, the toolbox is installed by clicking "Add Folder", selecting the k-Wave toolbox folder, and clicking "save". The toolbox can be uninstalled in the same fashion.

For Linux users, using the "Set Path" dialog box requires write access to `pathdef.m`. This file can be found under `<...matlabroot...>/toolbox/local`. To find where MATLAB is installed, type `>> matlabroot` at the MATLAB command line.

Alternatively, the toolbox can be installed by adding the line

```
addpath('<...pathname...>/k-Wave Toolbox');
```

[2]The `>>` symbol is the default MATLAB command prompt and is used here to denote commands that are entered in the MATLAB command window. The symbol itself is not actually entered.

to the `startup.m` file, where `<...pathname...>` is replaced with the location of the toolbox, and the slashes should be in the direction native to your operating system. If no `startup.m` file exists, create one, and save it in the MATLAB startup directory.

After installation, restart MATLAB. You should then be able to see the k-Wave help files in the MATLAB help browser. Try selecting one of the examples and then clicking "run the file". If you can't see "k-Wave Toolbox" in the contents list of the MATLAB help browser, try typing `>> help k-Wave` at the command prompt to see if the toolbox has been installed correctly. If it has and you still can't see the help files, open "Preferences" and select "Help" and make sure "k-Wave Toolbox" or "All Products" is checked.

After installation, to make the k-Wave documentation searchable from within the MATLAB help browser, run

```
>> builddocsearchdb('<...pathname...>/k-Wave Toolbox/helpfiles');
```

again using the slash direction native to your operating system. Note, the created database file will only work with the version of MATLAB used to create it.

If using the C++ or CUDA versions of `kspaceFirstOrder3D` (see discussion in Chapter 4), the appropriate binaries (and library files if using Windows) should also be downloaded from `http://www.k-wave.org/download.php` and placed in the root "binaries" folder of the toolbox.

1.5 License

k-Wave © 2009-2016 Bradley Treeby, Ben Cox, and Jiri Jaros.

The k-Wave toolbox is distributed by the copyright owners under the terms of the GNU Lesser General Public License (LGPL). This is a set of additional permissions added to the GNU General Public License (GPL). The full text of both licenses is included with the toolbox in the folder "license" or is available online from `http://www.gnu.org/licenses/`.

The LGPL license places copyleft restrictions on the k-Wave toolbox. Essentially, anyone can use the software for any purpose (commercial or non-commercial), the source code for the toolbox is freely available, and anyone can redistribute the software (in its original form or modified) as long as the distributed product comes with the full source code and is also licensed under the LGPL. You can make private modified versions of the toolbox without any obligation to divulge the modifications so long as the modified software is not distributed to anyone else. The copyleft restrictions only apply directly to the toolbox, but not to other (non-derivative) software that simply links to or uses the toolbox.

k-Wave is distributed in the hope that it will be useful, but WITHOUT ANY WARRANTY; without even the implied warranty of MERCHANTABILITY or FITNESS FOR A PARTICULAR PURPOSE. See the GNU Lesser General Public License for more details.

If you find the toolbox useful for your academic work, please consider citing one or more of the following:

> B. E. Treeby and B. T. Cox, "k-Wave: MATLAB toolbox for the simulation and reconstruction of photoacoustic wave-fields," Journal of Biomedical Optics, vol. 15, no. 2, p. 021314, 2010.

> B. E. Treeby, J. Jaros, A. P. Rendell, and B. T. Cox, "Modeling nonlinear ultrasound propagation in heterogeneous media with power law absorption using a k-space pseudospectral method," Journal of the Acoustical Society of America, vol. 131, no. 6, pp. 4324-4336, 2012.

> B. E. Treeby, J. Jaros, D. Rohrbach, and B. T. Cox, "Modelling Elastic Wave Propagation Using the k-Wave MATLAB Toolbox," IEEE International Ultrasonics Symposium, pp. 146-149, 2014.

The first paper gives an overview of the toolbox with applications in photoacoustics, the second describes the nonlinear ultrasound model and the C++ code, and the third describes the elastic code.

1.6 Alternative Software

The k-Wave toolbox is a powerful tool for general acoustic modelling. However, this doesn't mean it's the best tool for every purpose! There is a diverse range of other software packages available that might be more appropriate in particular circumstances. We try to maintain a list of useful acoustic packages at `http://www.k-wave.org/acousticsoftware.php`. If you think we've made any errors or omissions, please get in touch.

Chapter 2

Numerical Model

2.1 Governing Equations

When an acoustic wave passes through a compressible medium, there are dynamic fluctuations in the pressure, density, temperature, particle velocity, etc. These changes can be described by a series of coupled first-order partial differential equations based on the conservation of mass, momentum, and energy within the medium. Often in acoustics, these equations are combined together into a single "wave equation" which is a second-order partial differential equation in a single acoustic variable (most often the acoustic pressure). For example, in the classical case of a small amplitude acoustic wave propagating through a homogeneous and lossless fluid medium, the first-order equations are given by [5]

$$\begin{aligned}
\frac{\partial \mathbf{u}}{\partial t} &= -\frac{1}{\rho_0}\nabla p \ , && \text{(momentum conservation)} \\
\frac{\partial \rho}{\partial t} &= -\rho_0 \nabla \cdot \mathbf{u} \ , && \text{(mass conservation)} \\
p &= c_0^2 \rho \ . && \text{(pressure-density relation)}
\end{aligned} \tag{2.1}$$

Here $\mathbf{u}$ is the acoustic particle velocity, p is the acoustic pressure, ρ is the acoustic density, ρ_0 is ambient (or equilibrium) density, and c_0 is the isentropic sound speed. These equations assume the background medium is quiescent (meaning there is no net flow and the other ambient parameters don't change with time) and isotropic (meaning the material parameters do not depend on the direction the wave is travelling). When they are combined together, they give the familiar second-order wave equation

$$\nabla^2 p - \frac{1}{c_0^2}\frac{\partial^2 p}{\partial t^2} = 0 \ . \tag{2.2}$$

The main simulation functions in k-Wave (`kspaceFirstOrder1D`, `kspaceFirstOrder2D`, `kspaceFirstOrder3D`) solve the coupled first-order system of equations rather than the equivalent second-order equation. This is done for several reasons. First, it allows both mass and force sources to be easily included into the discrete equations. Second, it allows the use of a special anisotropic layer (known as a perfectly matched layer or PML) for

absorbing the acoustic waves when they reach the edges of the computational domain. Finally, the calculation of the particle velocity allows quantities such as the acoustic intensity to be calculated. This is useful, for example, when modelling how ultrasound heats biological tissue due to acoustic absorption.

The complexity of the governing equations used in k-Wave depends on the properties of the simulation set by the user. Often, the acoustic medium is heterogeneous, with a spatially varying sound speed and ambient density. In this case, the governing equations must include some additional terms. Similarly, as an acoustic wave propagates, it generally loses some acoustic energy to random thermal motion resulting in acoustic absorption. When the absorption parameters are defined (`medium.alpha_coeff` and `medium.alpha_power`), k-Wave treats the medium as a sound-absorbing fluid in which the absorption follows a frequency power law of the form

$$\alpha = \alpha_0 \omega^y \ , \tag{2.3}$$

where α is the absorption coefficient in units of $\mathrm{Np\,m^{-1}}$, α_0 is the power law prefactor in $\mathrm{Np\,(rad/s)^{-y}\,m^{-1}}$, and y is the power law exponent. Absorption of this form is observed in a number of different materials including marine sediments and biological tissue [6, 7]. This type of absorption model is accurate for situations in which the shear modulus is negligible, such as is often the case in soft biological tissue.

When acoustic absorption and heterogeneities in the material parameters are included, the system of coupled first-order partial differential equations becomes [8, 9]

$$\begin{aligned}
\frac{\partial \mathbf{u}}{\partial t} &= -\frac{1}{\rho_0}\nabla p \ , && \text{(momentum conservation)} \\
\frac{\partial \rho}{\partial t} &= -\rho_0 \nabla \cdot \mathbf{u} - \mathbf{u}\cdot\nabla\rho_0 \ , && \text{(mass conservation)} \\
p &= c_0^2\left(\rho + \mathbf{d}\cdot\nabla\rho_0 - \mathrm{L}\rho\right) \ , && \text{(pressure-density relation)}
\end{aligned} \tag{2.4}$$

where $\mathbf{d}$ is the acoustic particle displacement. If the mass conservation equation and the pressure-density relation are solved together, the additional $\nabla\rho_0$ terms cancel each other, so they are not included in the discrete equations solved in k-Wave to improve computational efficiency.

The operator L in the pressure-density relation is a linear integro-differential operator that accounts for acoustic absorption and dispersion that follows a frequency power law. The presence of acoustic absorption must physically be accompanied by dispersion (a dependence of the sound speed on frequency) to obey causality [10]. The operator used in k-Wave has two terms both dependent on a fractional Laplacian and is given by [11, 12]

$$\mathrm{L} = \tau \frac{\partial}{\partial t}\left(-\nabla^2\right)^{\frac{y}{2}-1} + \eta\left(-\nabla^2\right)^{\frac{y+1}{2}-1} \ . \tag{2.5}$$

Here τ and η are absorption and dispersion proportionality coefficients

$$\tau = -2\alpha_0 c_0^{y-1}, \qquad \eta = 2\alpha_0 c_0^{y}\tan\left(\pi y/2\right), \tag{2.6}$$

where α_0 is the power law prefactor in Np $\mathrm{(rad/s)^{-y}\,m^{-1}}$, and y is the power law exponent. The two terms in L separately account for power law absorption and dispersion for

$0 < y < 3$ and $y \neq 1$ under particular smallness conditions [12, 13]. These conditions are generally satisfied for the range of attenuation parameters observed in soft biological tissue (for very high values of absorption and frequency the behaviour of the loss operator deviates from a power law due to second-order effects [14, 13]).

In many situations in biomedical ultrasonics, the magnitude of the acoustic waves is high enough that the wave propagation is no longer linear. In this case, additional nonlinear terms also need to be included in the governing equations [15]. k-Wave doesn't model all the possible nonlinear effects that might occur in a fluid; it is not a computational fluid dynamics (CFD) solver. Instead, it currently includes two additional nonlinear terms that account for cumulative nonlinear effects to second-order in the acoustic variables. This is an accurate model for many situations in biomedical ultrasound. When the nonlinearity parameter `medium.BonA` is defined by the user, the system of coupled first-order equations solved by k-Wave becomes [2, 16]

$$
\begin{aligned}
\frac{\partial \mathbf{u}}{\partial t} &= -\frac{1}{\rho_0}\nabla p \ , && \text{(momentum conservation)}\\
\frac{\partial \rho}{\partial t} &= -\left(2\rho + \rho_0\right)\nabla\cdot\mathbf{u} - \mathbf{u}\cdot\nabla\rho_0 \ , && \text{(mass conservation)}\\
p &= c_0^2\left(\rho + \mathbf{d}\cdot\nabla\rho_0 + \frac{B}{2A}\frac{\rho^2}{\rho_0} - \mathrm{L}\rho\right) \ . && \text{(pressure-density relation)}
\end{aligned}
\tag{2.7}
$$

Here B/A is the nonlinearity parameter which characterises the relative contribution of finite-amplitude effects to the sound speed [17]. Compared to the linear case, the mass conservation equation includes an additional term which accounts for a convective nonlinearity in which the particle velocity contributes to the wave velocity [18]. The additional term is written as a spatial (rather than temporal) gradient to make it efficient to numerically encode [2]. The four terms within the bracket in the pressure-density relation separately account for linear wave propagation, heterogeneities in the ambient density, material nonlinearity, and power law absorption and dispersion (the sound speed c_0 and the nonlinearity parameter B/A can also be heterogeneous). Again, the additional $\nabla\rho_0$ terms cancel each other when these equations are solved together, so they are not included in the discrete equations solved in k-Wave. If the three coupled equations are combined, they give a generalised form of the Westervelt equation [16, 19, 20].

2.2 Acoustic Source Terms

The equations given in the previous section describe how acoustic waves propagate under various conditions, but they don't describe how these waves are generated or added to the medium. Theoretically, linear sources could be realised by adding a source term to any of the equations of mass, momentum, or energy conservation [21]. (There are also nonlinear acoustics sources, such as the emission of sound by turbulence, but these aren't considered here). For example, adding a source term to the momentum and mass conservation

equations describing linear wave propagation in a homogeneous medium gives

$$\frac{\partial \mathbf{u}}{\partial t} = -\frac{1}{\rho_0}\nabla p + \mathbf{S}_{\mathrm{F}} \ , \qquad \text{(momentum conservation)}$$
$$\frac{\partial \rho}{\partial t} = -\rho_0 \nabla \cdot \mathbf{u} + \mathrm{S}_{\mathrm{M}} \ , \qquad \text{(mass conservation)}$$
$$p = c_0^2 \rho \ . \qquad \text{(pressure-density relation)} \tag{2.8}$$

Here $\mathbf{S}_{\mathrm{F}}$ is a force source term and represents the input of body forces per unit mass in units of $\mathrm{N\,kg^{-1}}$ or $\mathrm{m\,s^{-2}}$. S_{M} is a mass source term and represents the time rate of the input of mass per unit volume in units of $\mathrm{kg\,m^{-3}\,s^{-1}}$ (the term $\mathrm{S}_{\mathrm{M}}/\rho_0$ in units of $\mathrm{s^{-1}}$ is sometimes called the volume velocity). In the corresponding second-order wave equation, the source terms appear as

$$\nabla^2 p - \frac{1}{c_0^2}\frac{\partial^2 p}{\partial t^2} = \rho_0 \nabla \cdot \mathbf{S}_{\mathrm{F}} - \frac{\partial}{\partial t}\mathrm{S}_{\mathrm{M}} \ . \tag{2.9}$$

This illustrates that it is actually the spatial gradient of the applied force, and the time rate of change of the rate of mass injection (volumetric acceleration) that give rise to sound [22].

Classical examples of mass or volume velocity sources are vibrating pistons and radially oscillating spheres (in general, bodies whose volume is oscillating). An example of a force source is a sideways oscillating rigid object, such as a wire or rigid sphere. The primary difference between mass and force sources is the directivity of the generated sound fields. As force is a vector, a force source has an inherent direction associated with it. A point force source acting in one-direction will thus produce a dipole field. In contrast, a pressure source will radiate in all directions (although it's possible for the shape of a pressure transducer to focus the field more strongly in one direction than another). A point mass source will thus produce a monopole field. Within k-Wave, force and mass sources are applied as velocity and pressure (or density) sources, respectively.

It's also possible to define a source term S_{H} associated with the energy conservation equation [21]. This corresponds to the injection of heat per unit volume per unit time, for example, due to the absorption of energy from a modulated laser beam. If the rate of heat input is sufficiently rapid that thermal diffusion can be neglected, heat sources can be treated as mass sources, where

$$\mathrm{S}_{\mathrm{M}} = \mathrm{S}_{\mathrm{H}}\beta / C_p \ . \tag{2.10}$$

Here, β is the volume thermal expansivity in units of $\mathrm{K^{-1}}$, and C_p is the constant pressure specific heat capacity in $\mathrm{J\,kg^{-1}\,K^{-1}}$. In the case of photoacoustic tomography, the heating pulse typically occurs on a timescale much shorter than the characteristic acoustic travel time (a condition called stress confinement), and so the source can also be modelled as an initial value problem for the acoustic pressure [23].

2.3 Overview of the k-space pseudospectral method

There are a wide variety of different numerical methods available for the solution of partial differential equations. There are an even greater variety if you consider the different

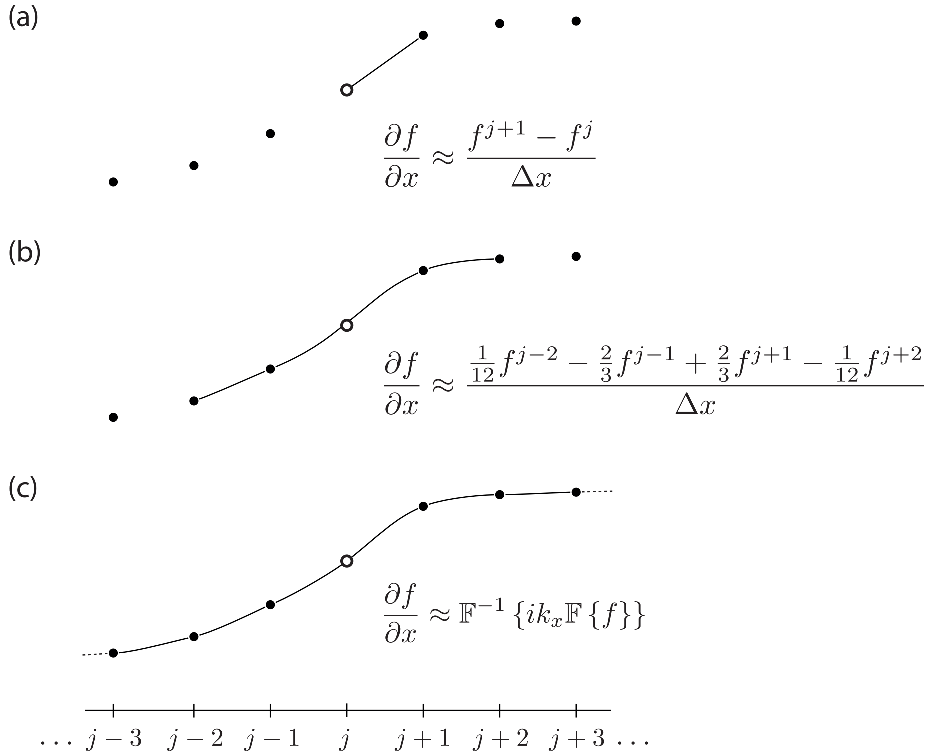

Figure 2.1: Calculation of spatial gradients using local and global methods. (a) First-order accurate forward difference. (b) Fourth-order accurate central difference. (c) Fourier collocation spectral method.

possible permutations for each method. The "best" approach for discretising a particular problem depends on many factors. For example, the size of the computational domain, the number of frequencies of interest, the properties of the medium, the types of boundary conditions, and so on. Here, we are interested in the time domain solution of the wave equation for broadband acoustic waves in heterogeneous media. The drawback with classical finite difference and finite element approaches for solving this type of problem is that at least 10 grid points per acoustic wavelength are generally required to achieve a useful level of accuracy (a level of accuracy on a par with the uncertainty in the user-defined inputs). This often results in computational grids that are simply to big to solve using normal computers. To take an example, a diagnostic ultrasound image formed using a 3 MHz curvilinear transducer has a depth penetration around 15 cm. This distance is on the order of 300 acoustic wavelengths at the fundamental frequency, and 600 wavelengths at the second harmonic. If the acoustic parameters need to be discretised using 10 grid points per wavelength, this translates into a 3D computational domain with more than 10^{11} grid elements. Even storing one matrix of this size in single-precision requires more than 400 GB of computer memory! This problem is confounded further by the requirement for small time steps to keep the simulation stable and to minimise unwanted numerical errors.

To reduce the memory and number of time steps required for accurate simulations, k-Wave solves the system of coupled acoustic equations described in the previous sections using

the k-space pseudospectral method (or k-space method) [24, 25, 26, 27]. This combines the spectral calculation of spatial derivatives (in this case using the Fourier collocation method) with a temporal propagator expressed in the spatial frequency domain or k-space. In a standard finite difference scheme, spatial gradients are computed locally based on the function values at neighbouring grid points. In the simplest case, the gradient of the field can be estimated using linear interpolation (see Fig. 2.1). A better estimate of the gradient can be obtained by fitting a higher-order polynomial to a greater number of grid points and calculating the derivative of the polynomial [28]. The more points used, the higher the degree of polynomial required, and the more accurate the estimate of the derivative. The Fourier collocation spectral method takes this idea further and fits a Fourier series to all of the data [29]. It is therefore sometimes referred to as a global, rather than local, method. There are two significant advantages to using Fourier series. First, the amplitudes of the Fourier components can be calculated efficiently using the fast Fourier transform (FFT). Second, the basis functions are sinusoidal, so only two grid points (or nodes) per wavelength are theoretically required, rather than the six to ten required in other methods.

While the Fourier collocation spectral method improves efficiency in the spatial domain, conventional finite difference schemes are still needed to calculate the gradients in the time domain. For example, using the second-order wave equation for homogeneous and lossless media

$$\nabla^2 p\left(\mathbf{x},t\right) - \frac{1}{c_0^2}\frac{\partial^2}{\partial t^2} p\left(\mathbf{x},t\right) = 0 \ , \qquad (2.11)$$

a simple pseudospectral solution can be derived by taking the spatial Fourier transform and then discretising the time derivative using a second-order accurate central difference[1]

$$\frac{p\left(\mathbf{k},t+\Delta t\right) - 2p\left(\mathbf{k},t\right) + p\left(\mathbf{k},t-\Delta t\right)}{\Delta t^2} = -\left(c_0 k\right)^2 p\left(\mathbf{k},t\right) \ . \qquad (2.12)$$

Here $k^2 = \mathbf{k}\cdot\mathbf{k} = k_x^2 + k_y^2 + k_z^2$, where $\mathbf{k}$ is the wavevector, Δt is the spacing between time points, and we have used the relationship for the Fourier transform of the derivative of a bounded function

$$\mathcal{F}\left\{\frac{\partial}{\partial x} f(x)\right\} = -\frac{1}{2\pi}\int f(x)(-ik_x)e^{-ik_x x}\,dx = ik_x\,\mathcal{F}\left\{f(x)\right\} \ , \qquad (2.13)$$

where $\mathcal{F}$ is the spatial Fourier transform. Unfortunately, the finite difference approximation of the temporal derivative introduces errors into the numerical solution that can only be controlled by limiting the size of the time-step. The techniques broadly classed as k-space methods attempt to relax this limitation in order to allow larger time-steps to be used without compromising accuracy. Using an exact solution to the homogeneous and lossless wave equation valid for an initial pressure distribution [27, 23, 14]

$$p\left(\mathbf{k},t\right) = \cos\left(c_0 k t\right) p\left(\mathbf{k},0\right) \ , \qquad (2.14)$$

[1] This is the general approach for Fourier pseudospectral and k-space methods; by taking the spatial Fourier transform of the equations, time dependent partial differential equations are reduced to ordinary differential equations that can be integrated forward in time using implicit or explicit methods.

an exact pseudospectral scheme for Eq. (2.11) can be derived by substituting Eq. (2.14) into the leapfrog finite difference $p(\mathbf{k}, t + \Delta t) - 2p(\mathbf{k}, t) + p(\mathbf{k}, t - \Delta t)$. After some rearrangement, this yields the relationship [27]

$$\frac{p(\mathbf{k}, t + \Delta t) - 2p(\mathbf{k}, t) + p(\mathbf{k}, t - \Delta t)}{\Delta t^2 \operatorname{sinc}^2(c_0 k \Delta t/2)} = -(c_0 k)^2 p(\mathbf{k}, t) \quad . \tag{2.15}$$

By comparing the two pseudospectral schemes, we can see that the Δt^2 term in Eq. (2.12) has been replaced with $\Delta t^2 \operatorname{sinc}^2(c_0 k \Delta t/2)$ in Eq. (2.15). For small Δt, these are approximately the same. However, for larger time steps, the additional sinc term provides an exact solution, free from numerical dispersion. By extension, an exact pseudospectral scheme for solving the acoustic equations expressed as coupled first-order partial differential equations can be obtained by replacing Δt in a first-order accurate forward difference with $\Delta t \operatorname{sinc}(c_0 k \Delta t/2)$ [27, 30]. The operator

$$\kappa = \operatorname{sinc}(c_{\text{ref}} k \Delta t/2) \quad , \tag{2.16}$$

is known as the k-space operator, where c_{ref} is a scalar reference sound speed.

For large-scale acoustic simulations where the waves propagate over distances of hundreds or thousands of wavelengths, this seemingly small correction becomes critically important. Without this term, the finite difference approximation of the temporal derivative introduces phase errors which accumulate as the simulation runs. For small simulations, this accumulation is generally not a problem. However, to retain the same level of accuracy as the size of the simulation is increased, the size of the time steps must be continually reduced. This can significantly increase compute times, particularly in comparison to the k-space method which remains dispersion free, regardless of the simulation size. When nonlinearity, heterogeneous material parameters, or acoustic absorption are included in the governing equations, the temporal discretisation using the k-space operator is no longer exact. However, if these perturbations are small, the inclusion of this operator can still significantly reduce the unwanted numerical dispersion [26, 27, 2].

As well as the use of the k-space operator, additional accuracy and stability can also be obtained when computing odd-order derivatives by using staggered spatial and temporal grids [31]. For the Fourier collocation spectral method, spatial shifts can be easily obtained using the shift property of the Fourier transform, where $\mathcal{F}_x\{f(x + \Delta x)\} = e^{ik_x \Delta x} \mathcal{F}_x\{f(x)\}$. Details of the staggered grid scheme used in k-Wave are given in the following section.

Rather than using a Fourier basis to calculate the spatial gradients, it is also possible to use an alternative form of the pseudospectral method that uses Chebyshev polynomials [32]. There are several reasons why the Fourier method, rather than the Chebyshev method, is used in k-Wave. First, it is straightforward to calculate the k-space operator when the gradients are computed using a Fourier basis, giving improved accuracy for large time steps as mentioned above. Second, the time step required for stability when using explicit time stepping schemes scales with N^{-2} for the Chebyshev method, and N^{-1} for the Fourier method, where N is the number of grid points in each Cartesian direction given a fixed domain size [33, 34]. This makes the Fourier spectral method significantly cheaper, particularly for large-scale problems. Third, when using Chebyshev polynomials, the grid points must be clustered closer together near the boundaries to avoid the Runge phenomenon [32, 35]. This means for the same maximum frequency, more grid points

are needed. For example, a common choice is cosine-spaced points [35]. Compared to the Fourier method, this would require $(\pi/2)^N$ more grid points for an N-dimensional simulation. For 3D simulations, this increases the memory consumption by almost four times. Fourth, (although perhaps less importantly), using a Fourier basis is more intuitive to acousticians who often think in the wavenumber-frequency domain. The main argument in favour of using Chebyshev polynomials is that they do not make the assumption of periodicity, and are therefore compatible with a range of boundary conditions. However, for simulations in infinite domains, it is straightforward to counteract the periodicity assumed by the Fourier method using a perfectly matched layer (see discussion in Sec. 2.6).

2.4 Discrete k-space Equations

Starting with the linear case, the mass and momentum conservation equations in Eq. (2.4) written in discrete form using the k-space pseudospectral method become

$$\frac{\partial}{\partial \xi} p^n = \mathcal{F}^{-1}\left\{ i k_\xi \, \kappa \, e^{i k_\xi \Delta\xi/2} \mathcal{F}\left\{ p^n \right\} \right\} \ , \tag{2.17a}$$

$$u_\xi^{n+\frac{1}{2}} = u_\xi^{n-\frac{1}{2}} - \frac{\Delta t}{\rho_0} \frac{\partial}{\partial \xi} p^n + \Delta t \, \mathrm{S}_{\mathrm{F}_\xi}^{\mathrm{n}} \ , \tag{2.17b}$$

$$\frac{\partial}{\partial \xi} u_\xi^{n+\frac{1}{2}} = \mathcal{F}^{-1}\left\{ i k_\xi \, \kappa \, e^{-i k_\xi \Delta\xi/2} \mathcal{F}\left\{ u_\xi^{n+\frac{1}{2}} \right\} \right\} \ , \tag{2.17c}$$

$$\rho_\xi^{n+1} = \rho_\xi^n - \Delta t \rho_0 \frac{\partial}{\partial \xi} u_\xi^{n+\frac{1}{2}} + \Delta t \, \mathrm{S}_{\mathrm{M}_\xi}^{\mathrm{n}+\frac{1}{2}} \ . \tag{2.17d}$$

Equations (2.17a) and (2.17c) are spatial gradient calculations based on the Fourier collocation spectral method, while (2.17b) and (2.17d) are update steps based on a k-space corrected first-order accurate forward difference. These equations are repeated for each Cartesian direction in $\mathbb{R}^N$ where $\xi = x$ in $\mathbb{R}^1$, $\xi = x, y$ in $\mathbb{R}^2$, and $\xi = x, y, z$ in $\mathbb{R}^3$ (N is the number of spatial dimensions). Here, $\mathcal{F}$ and $\mathcal{F}^{-1}$ denote the forward and inverse spatial Fourier transform, i is the imaginary unit, k_ξ represents the wavenumbers in the ξ direction, $\Delta\xi$ is the grid spacing in the ξ direction, Δt is the time step, and κ is the k-space operator defined in Eq. (2.16). The discrete wavenumbers are defined according to

$$k_\xi = \begin{cases} \left[-\frac{\mathrm{N}_\xi}{2}, -\frac{\mathrm{N}_\xi}{2}+1, \ldots, \frac{\mathrm{N}_\xi}{2}-1\right] \frac{2\pi}{\Delta\xi \, \mathrm{N}_\xi} & \text{if } \mathrm{N}_\xi \text{ is even} \\ \left[-\frac{(\mathrm{N}_\xi-1)}{2}, -\frac{(\mathrm{N}_\xi-1)}{2}+1, \ldots, \frac{(\mathrm{N}_\xi-1)}{2}\right] \frac{2\pi}{\Delta\xi \, \mathrm{N}_\xi} & \text{if } \mathrm{N}_\xi \text{ is odd} \end{cases} \tag{2.17e}$$

where N_ξ is the number of grid points in the ξ direction (this is discussed further in Sec. 3.2). The acoustic density (which is physically a scalar quantity) is artificially divided into Cartesian components to allow an anisotropic perfectly matched layer to be applied (this is discussed in Sec. 2.6). The exponential terms $e^{\pm i k_\xi \Delta\xi/2}$ within Eqs. (2.17a) and (2.17c) are spatial shift operators that translate the result of the gradient calculations by half the grid point spacing in the ξ-direction. This allows the components of the particle velocity to be evaluated on a staggered grid. An illustration of the staggered grid scheme is shown

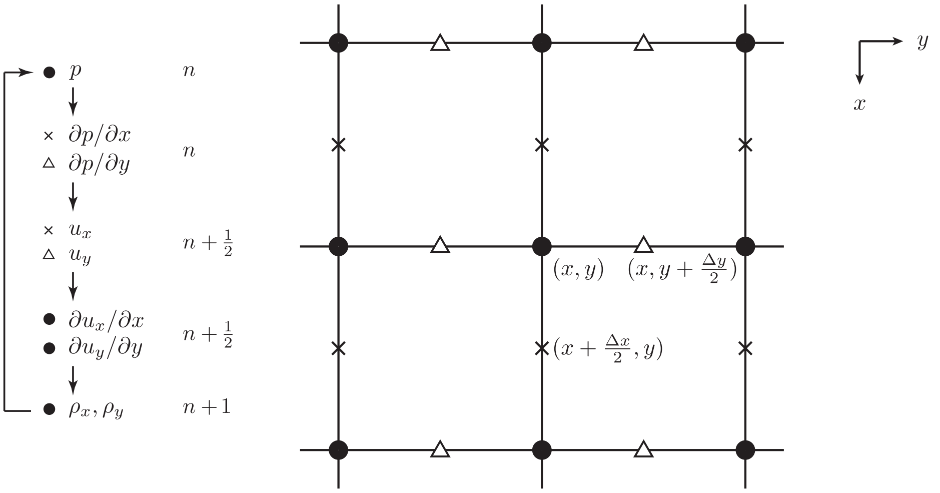

Figure 2.2: Schematic showing the computational steps in the solution of the coupled first-order equations using a staggered spatial and temporal grid in 2D. Here $\partial p/\partial x$ and u_x are evaluated at grid points staggered in the x-direction (crosses), while $\partial p/\partial y$ and u_y evaluated at grid points staggered in the y-direction (triangles). The remaining variables are evaluated on the regular grid (dots). The time staggering is denoted using n, $n+\frac{1}{2}$, and $n+1$.

in Fig. 2.2. Note, the density ρ_0 in Eq. (2.17b) is understood to be the ambient density defined at the staggered grid points.

The corresponding pressure-density relation is given by

$$p^{n+1} = c_0^2 \left(\rho^{n+1} - \mathrm{L_d}\right) \quad , \tag{2.17f}$$

where the total acoustic density is given by $\rho^{n+1} = \sum_\xi \rho_\xi^{n+1}$. Here $\mathrm{L_d}$ is the discrete form of the power law absorption term which is discussed in Sec. 2.5. In all the equations above, the superscripts n and $n+1$ denote the function values at current and next time points and $n-\frac{1}{2}$ and $n+\frac{1}{2}$ at the time staggered points. This time-staggering arises because the update steps, Eqs. (2.17b) and (2.17d), are interleaved with the gradient calculations, Eqs. (2.17a) and (2.17c).

The acoustic source terms defined in Eqs. (2.17b) and (2.17d) represent the input of body forces per unit mass, and the time rate of input of mass per unit volume (see Sec. 2.2). However, within k-Wave, the source terms defined by the user are given in units of acoustic pressure and velocity. (These inputs are called `source.p` and `source.ux`, `source.uy`, `source.uz`. Further discussion is given in Sec. 3.4). These terms are used because the available measurements of acoustic sources are typically either measurements of acoustic pressure or particle velocity. Consequently, the user inputs are scaled by k-Wave so they are in the correct units before they are added to the discrete equations.

The Cartesian components of the force source term $\mathrm{S}_{\mathrm{F}_\xi}$ are calculated from the user inputs `source.ux`, `source.uy`, `source.uz` by multiplying by $c_0/\Delta\xi$ (in units of s^{-1}) to convert from units of velocity ($\mathrm{m\,s^{-1}}$) to units of acceleration ($\mathrm{m\,s^{-2}}$). The components of the mass source term $\mathrm{S}_{\mathrm{M}_\xi}$ are calculated from the user input `source.p` by multiplying by $1/(Nc_0^2)$

Table 2.1: Effect of the staggered grid scheme on the input and output pressure and particle velocity values in 3D.

Parameter	Position	Time
x-direction velocity input	$x+\Delta x/2,\ y,\ z$	t
x-direction velocity output	$x+\Delta x/2,\ y,\ z$	$t+\Delta t/2$
y-direction velocity input	$x,\ y+\Delta y/2,\ z$	t
y-direction velocity output	$x,\ y+\Delta y/2,\ z$	$t+\Delta t/2$
z-direction velocity input	$x,\ y,\ z+\Delta z/2$	t
z-direction velocity output	$x,\ y,\ z+\Delta z/2$	$t+\Delta t/2$
pressure input	$x,\ y,\ z$	$t+\Delta t/2$
pressure output	$x,\ y,\ z$	$t+\Delta t$

to convert from units of pressure to units of density, and by $c_0/\Delta\xi$ to convert from units of density to the time rate of density. The $1/N$ term divides the input between the split density components, where N is the number of dimensions. Using the x-direction as an example, the final source scaling factors used in k-Wave are

$$\mathrm{S}_{\mathrm{F}_x} = \texttt{source.ux}\frac{2c_0}{\Delta x} \ , \tag{2.18}$$

$$\mathrm{S}_{\mathrm{M}_x} = \frac{\texttt{source.p}}{c_0^2 N}\frac{2c_0}{\Delta x} \ . \tag{2.19}$$

When the sound speed is heterogeneous, the values of the sound speed at the source positions are used.

One disadvantage of the staggered grid scheme used in k-Wave is that user inputs and outputs must also follow this scheme. This means inputs and outputs for the particle velocity are defined on staggered grid points, while inputs and outputs for the pressure are defined on regular grid points. This is further complicated by the staggered time scheme, as the outputs for both pressure and velocity are offset by $\Delta t/2$ relative to the inputs. However, with a little care, it is possible to compensate for these offsets. The effect of the staggered grid scheme on the inputs and outputs is summarised in Table 2.1.

The time staggering also affects how the initial conditions are defined for an initial value problem (IVP). For example, when modelling an IVP for the pressure for which the particle velocity is zero at time $t = 0$ (this is the case in photoacoustic imaging), it is not possible to directly impose $u_\xi^0 = 0$. Instead, it is necessary to impose odd symmetry by setting $u_\xi^{-1/2} = -u_\xi^{1/2}$. This is done automatically within the simulation functions when the user sets a value for `source.p0` (a discussion of the source terms is given in Sec. 3.4).

Returning to the discrete equations, in the nonlinear case, the mass conservation equation also includes a convective nonlinearity term, and thus Eq. (2.17d) becomes

$$\rho_\xi^{n+1} = \frac{\rho_\xi^n - \Delta t \rho_0 \frac{\partial}{\partial \xi} u_\xi^{n+\frac{1}{2}}}{1 + 2\Delta t \frac{\partial}{\partial \xi} u_\xi^{n+\frac{1}{2}}} + \frac{\Delta t\, \mathrm{S}_{\mathrm{M}_\xi}^{\mathrm{n}+\frac{1}{2}}}{1 + 2\Delta t \frac{\partial}{\partial \xi} u_\xi^{n+\frac{1}{2}}} \ . \tag{2.20}$$

The nonlinear correction to the mass source term arises because the temporal gradient in the mass conversation equation from Eq. (2.7) is solved using an implicit finite difference scheme (the acoustic density term on the right hand side is taken to be ρ^{n+1} rather than ρ^n). Because the effect of the nonlinear term on the source is small, it is neglected in the discrete equations implemented in k-Wave. The corresponding pressure-density relation includes a material nonlinearity term and is given by

$$p^{n+1} = c_0^2 \left(\rho^{n+1} + \frac{B}{2A} \frac{1}{\rho_0} \left(\rho^{n+1}\right)^2 - \mathrm{L_d} \right) \ , \tag{2.21}$$

where the total acoustic density is again given by $\rho^{n+1} = \sum_\xi \rho_\xi^{n+1}$.

The calculation of first-order gradients using the Fourier collocation spectral method normally requires a Fourier transform over only one dimension. For example, to compute the gradient in the x-direction, the Fourier transform is performed over the x-dimension, the result is multiplied by ik_x (the wavenumbers in the x-direction), and the inverse Fourier transform is then performed. A penalty of including the k-space operator κ in the discrete equations is that the Fourier transform must be performed over $\mathbb{R}^N$ rather than $\mathbb{R}^1$. In other words, for a 3D simulation, the Fourier transforms must be three dimensional. This is because the k-space operator depends on the scalar wavenumber k, given by

$$k = \sqrt{\mathbf{k} \cdot \mathbf{k}} = \sqrt{k_x^2 + k_y^2 + k_z^2} \ , \tag{2.22}$$

which varies in all three dimensions. The major advantage is that for homogeneous media, the inclusion of the k-space operator makes the temporal discretisation exact. This means the time steps can be made arbitrarily large to compensate for this penalty. In the heterogeneous case, for small simulations a rough rule of thumb is that the operator allows the time steps to be three times larger for a similar level of accuracy (although this is very problem dependent [27, 36, 2]). For most simulations, the calculation of Fourier transforms accounts for about 60% of the total compute time [37]. Thus, even after accounting for the increase in time to calculate the Fourier transforms, the k-space approach still reduces the overall compute time on the order of 50% in 2D, and 25% in 3D. The advantage of the k-space method becomes more marked as the size of the simulation is increased because of the accumulation of phase error (see discussion in Sec. 2.3).

2.5 Modelling Power Law Acoustic Absorption

The acoustic absorption in most biological tissues over the MHz frequency range has been experimentally observed to follow a frequency power law [38]. As mentioned in Sec. 2.1, k-Wave uses an absorption term based on the fractional Laplacian to account for this behaviour [11, 12]. Compared to absorption operators based on temporal fractional derivatives [39, 40, 41, 42, 43, 44], the advantage of this form of the absorption term is that it can be computed efficiently using Fourier spectral methods [12, 2]. The principal alternative is to include a sum of relaxation absorption terms [45, 27]. However, this is more memory intensive and requires the relaxation parameters to be obtained using a fitting procedure for each value of absorption and range of frequencies under consideration.

Returning to the discretised equations, the spatial Fourier transform of the negative fractional Laplacian has the simple form [46, 11]

$$\mathcal{F}\left\{\left(-\nabla^2\right)^a \rho\right\} = k^{2a}\mathcal{F}\left\{\rho\right\} \ ,$$

which allows the discrete form of the power law absorption term to be written as [12]

$$\mathrm{L_d} = \tau\,\mathcal{F}^{-1}\left\{k^{y-2}\,\mathcal{F}\left\{\frac{\partial \rho^n}{\partial t}\right\}\right\} + \eta\,\mathcal{F}^{-1}\left\{k^{y-1}\,\mathcal{F}\left\{\rho^{n+1}\right\}\right\} \ . \tag{2.23}$$

To avoid needing to explicitly calculate the time derivative of the acoustic density (which would require storing a copy of at least ρ^n and ρ^{n-1} in memory), the temporal derivative of the acoustic density is replaced using the linearized mass conservation equation $\partial\rho/\partial t = -\rho_0\nabla\cdot\mathbf{u}$, which gives

$$\mathrm{L_d} = -\tau\,\mathcal{F}^{-1}\left\{k^{y-2}\,\mathcal{F}\left\{\rho_0 \sum\nolimits_\xi \frac{\partial}{\partial \xi} u_\xi^{n+\frac{1}{2}}\right\}\right\} + \eta\,\mathcal{F}^{-1}\left\{k^{y-1}\,\mathcal{F}\left\{\rho^{n+1}\right\}\right\} \ . \tag{2.24}$$

It is clear from the notation used here that the numerical values for the acoustic density and particle velocity are temporally offset by $dt/2$. This introduces an additional phase offset between the acoustic density and the pressure, which causes a small error in the modelled values of absorption and dispersion (using a simple finite difference approximation to $\partial\rho/\partial t$ also results in a similar phase error). For most simulations, the accuracy of the modelled acoustic absorption and dispersion should be sufficient. If increased numerical precision is required, the size of the time step can be reduced.

2.6 Perfectly Matched Layer

In Fourier pseudospectral and k-space numerical models, the use of the FFT to calculate spatial gradients implies that the wave field is periodic. This causes waves leaving one side of the domain to reappear at the opposite side. (In the 1D case, imagine a wave on a closed loop of string; in 2D think of a wave propagating on the surface of a torus; in 3D it is harder to imagine!) Often we want to model the propagation of acoustic waves in free space. This could be achieved by increasing the size of the computational grid so that the waves never reach the boundaries. However, this approach carries a significant computational penalty. Instead, we want the waves reaching the edge of the domain to disappear, as if they were continuing off to infinity, rather than “wrapping round” and re-appearing on the opposite side of the domain.

The wave wrapping caused by the FFT can be largely eliminated by the use a perfectly matched layer (PML) [47, 48]. This is a thin absorbing layer that encloses the computational domain and is governed by a nonphysical set of equations that cause anisotropic absorption. In pseudospectral models there are two requirements that such a layer must meet: (1) the layer must provide sufficient absorption so the outgoing waves are significantly attenuated, and (2) the layer must not reflect any waves back into the medium.

k-Wave uses Berenger’s original split-field formulation of the PML [47, 49]. This requires the acoustic density or pressure to be artificially divided into Cartesian components, where

$\rho = \rho_x + \rho_y + \rho_z$. The absorption is then defined such that only components of the wave field travelling within the PML and normal to the boundary are absorbed. Using the homogeneous linear case to illustrate, the first-order coupled equations including the PML become

$$\frac{\partial u_\xi}{\partial t} = -\frac{1}{\rho_0}\frac{\partial p}{\partial \xi} - \alpha_\xi u_\xi \ , \qquad \text{(momentum conservation)} \tag{2.25a}$$

$$\frac{\partial \rho_\xi}{\partial t} = -\rho_0 \frac{\partial u_\xi}{\partial \xi} - \alpha_\xi \rho_\xi \ , \qquad \text{(mass conservation)} \tag{2.25b}$$

$$p = c_0^2 \sum_\xi \rho_\xi \ . \qquad \text{(pressure-density relation)} \tag{2.25c}$$

Here $\boldsymbol{\alpha} = \{\alpha_x, \alpha_y, \alpha_z\}$ is the anisotropic absorption in Nepers per second. All three components are zero outside the PML, and inside the PML they are zero everywhere except within a PML layer perpendicular to their associated direction. In other words, for a PML perpendicular to the x-axis, $\boldsymbol{\alpha} = \{\alpha_x, 0, 0\}$. The fact that the absorption coefficient is anisotropic in this way, and that the same absorption coefficient acts on both the density and particle velocity, is sufficient for there to be no reflections from the edge of the PML (in the continuous homogeneous case).

Following [50, 27], Eqs. (2.25a) and (2.25b) are transformed using the relationship

$$\left(\frac{\partial}{\partial t} + \alpha\right) f + Q \ \rightarrow \ \frac{\partial}{\partial t}\left(e^{\alpha t} f\right) + e^{\alpha t} Q \ , \tag{2.26}$$

into the form

$$\frac{\partial}{\partial t}(e^{\alpha_\xi t} u_\xi) = -e^{\alpha_\xi t}\frac{1}{\rho_0}\frac{\partial p}{\partial \xi} \ , \qquad \frac{\partial}{\partial t}(e^{\alpha_\xi t} \rho_\xi) = -\rho_0 e^{\alpha_\xi t}\frac{\partial u_\xi}{\partial \xi} \ .$$

Using first-order accurate forward differences to discretise the time derivatives, the discrete equations given in Eq. (2.17b) and (2.17d) including a PML can then be written as

$$u_\xi^{n+\frac{1}{2}} = e^{-\alpha_\xi \Delta t/2}\left(e^{-\alpha_\xi \Delta t/2}\, u_\xi^{n-\frac{1}{2}} - \frac{\Delta t}{\rho_0}\frac{\partial}{\partial \xi} p^n\right) \ ,$$

$$\rho_\xi^{n+1} = e^{-\alpha_\xi \Delta t/2}\left(e^{-\alpha_\xi \Delta t/2}\, \rho_\xi^{n} - \Delta t \rho_0 \frac{\partial}{\partial \xi} u_\xi^{n+\frac{1}{2}}\right) \ . \tag{2.27}$$

This is the form of the PML equations implemented in k-Wave.

So far, nothing has been said about the actual values of α_ξ. It would seem from the equations above that large values should be used, as the waves will then be attenuated quickly, and the required thickness of the PML minimised. However, the spatial discretisation must also be taken into account. Consider the case of a wave propagating in the x direction. If α_x is constant, between the edge of the PML and one grid point inside, the wave will be forced to decrease by a factor of $\exp(-\alpha_x \Delta x / c_0)$. If α_x is large then the PML will impose a large gradient across the PML boundary, which will cause a reflection of the incoming wave. One way to reduce this reflection is to set $\alpha_x \ll c_0/\Delta x$. However, then the decay within the PML will be slow, and a very thick PML will be required to avoid significant wave wrapping. A better way is to make α_ξ a function of position within

the PML, where $\alpha_\xi = \alpha_\xi(\xi)$, so that the shape of the decay can be changed to make it smoother at the boundary edge. k-Wave uses the following function [27]

$$\alpha_\xi = \alpha_{\max} \left(\frac{\xi - \xi_0}{\xi_{\max} - \xi_0} \right)^m \quad , \tag{2.28}$$

where ξ_0 is the coordinate at the start of the PML and $\xi_{\max}$ is the coordinate at the end. Following Tabei et al., [27] $m = 4$ is used to give a balance between minimising the amplitude of the wrapped wave and minimising the amplitude of the reflected wave. Using a staggered spatial grid makes a significant improvement to the performance of the PML.

The PML absorption coefficient α_ξ used in the equations above is defined in units of Nepers s^{-1}. Within k-Wave, the absorption parameter `PML_alpha` is instead defined in normalised units of Nepers per grid point, where `PML_alpha` $= (\Delta\xi/c_0)\alpha_\xi$. The corresponding PML thickness `PML_size` is also defined in units of grid points. Figure 2.3 illustrates how the PML transmission and reflection coefficients change with variations in `PML_alpha` and `PML_size` for a normally incident plane wave. By default, k-Wave uses `PML_alpha = 2` and `PML_size = 20` for 1D and 2D simulations, and `PML_alpha = 2` and `PML_size = 10` for 3D simulations (the smaller size is used to save grid real-estate). For `PML_size = 10`, the amplitude of the transmitted wave is reduced by 84 dB, while the reflected coefficient is −65 dB. For `PML_size = 20`, the transmission and reflection coefficients are improved to −100 dB and −80 dB, respectively. This corresponds to around 4 or 5 decimal places of accuracy, which should be sufficient for most simulations (see discussion in Sec. 3.8). It is possible to change the values for `PML_alpha` and `PML_size` using the optional input parameters `'PMLAlpha'` and `'PMLSize'` (see discussion in Sec. 3.6).

Note, the formulation of the PML and the default PML values are based on the assumption of a homogeneous and lossless medium. For media with very strong acoustic absorption, the efficacy of the PML is reduced. The performance of the PML is also dependent on frequency and angle of incidence (see [51]).

2.7 Accuracy, Stability and the CFL Number

In the previous sections, the continuous equations describing the propagation of linear and nonlinear waves in heterogeneous and absorbing media, along with the discretisation of these equations using the *k*-space pseudospectral method have been discussed. Here we consider the question: when will the numerical model derived in Sec. 2.4 give the correct solution to the continuous governing equations discussed in Sec. 2.1? There are three aspects to this:

1. Are the discrete model equations equivalent to the continuous governing equations?
2. Is the numerical model stable?
3. Are the results it generates accurate?

The first question is asking whether the discrete equations are *consistent* or compatible with the continuous equations. In other words, whether they become the continuous

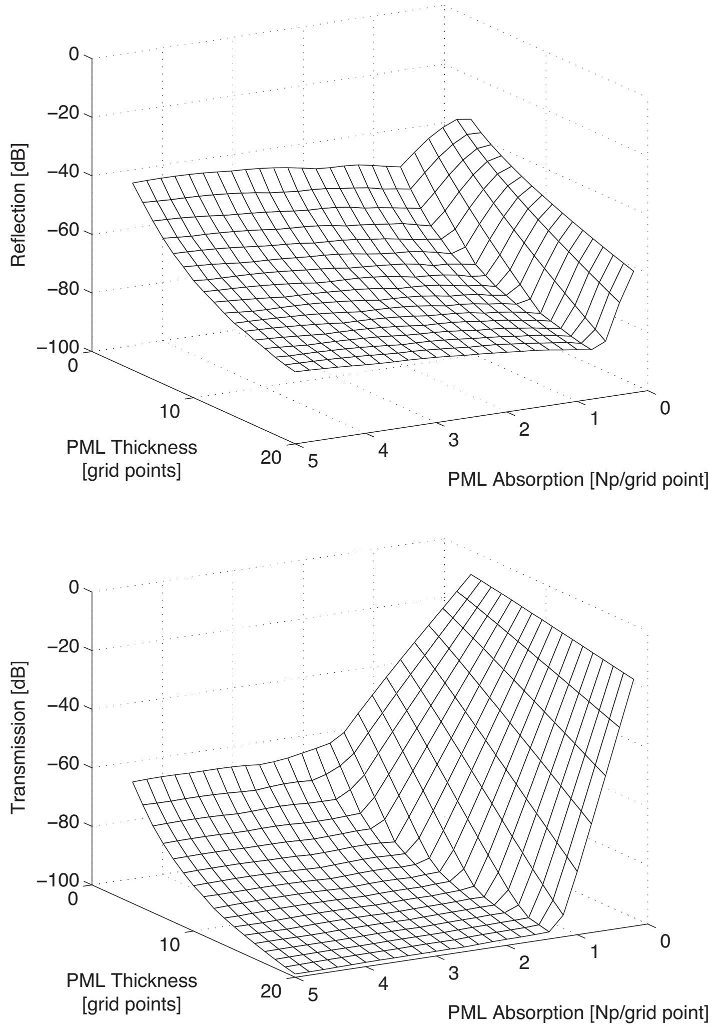

Figure 2.3: Performance of the split-field perfectly matched layer (PML) with variations in the layer thickness and absorption coefficient.

equations in the limit as the spacing between the discrete spatial and temporal points approaches zero, in the same way that the simple finite difference scheme $(p(t + \Delta t) - p(t))/\Delta t \rightarrow \partial p/\partial t$ as $\Delta t \rightarrow 0$. In this case, the discrete equations given in Eq. (2.17) are derived rigorously from the governing equations given in Eq. (2.4), and thus they are consistent with them.

The second question is whether the numerical model based on these discrete equations is *stable* or not. In other words, whether or not the numerical errors grow exponentially as the model steps through time. It is important to note that some consistent schemes are not stable. In other words, there are some numerical schemes derived directly from the continuous equations, and equal to them in the limit, whose output will never be a good approximation to the underlying system of partial differential equations.

Often, the stability or otherwise of a scheme depends on the size of the timestep, Δt. The stability condition for the discrete equations used in k-Wave can be derived straightfor-

wardly in the case of a homogeneous, non-absorbing medium. In this case the discrete equations given in Eq. (2.17) can be written in the simpler form

$$U_{k_\xi}^{n+\frac{1}{2}} = U_{k_\xi}^{n-\frac{1}{2}} - \frac{ik_\xi\,\kappa\,\Delta t}{\rho_0} P^n \quad , \tag{2.29a}$$

$$P^{n+1} = P^n - ik_\xi\,\kappa\,\Delta t \rho_0 c_0^2 U_{k_\xi}^{n+\frac{1}{2}} \quad , \tag{2.29b}$$

where $P^n(\mathbf{k}) = \mathcal{F}\{p^n(\mathbf{x})\}$ and $U_{k_\xi}^n(\mathbf{k}) = \mathcal{F}\{u_\xi^n(\mathbf{x})\}$ are the pressure and particle velocity variables in the spatial frequency or wavenumber domain. Writing the pressure at the previous time step as

$$P^n = P^{n-1} - ik_\xi\,\kappa\,\Delta t \rho_0 c_0^2 U_{k_\xi}^{n-\frac{1}{2}} \quad , \tag{2.30}$$

subtracting Eq. (2.30) from Eq. (2.29b) and substituting in Eq. (2.29a) then gives

$$P^{n+1} - 2P^n + P^{n-1} = -b^2 P^n \quad , \tag{2.31}$$

where $b = k\kappa\Delta t c_0$.

Equation (2.31) is in the form of a simple difference equation, and the range of values of b for which it generates a stable sequence $\ldots, P^{n-1}, P^n, P^{n+1}, \ldots$ can be found by assuming the solution at timestep n has the form $P^n = (A)^n B$, where the n on A indicates a power rather than a timestep index. A denotes the factor that is effectively multiplied to the old P to obtain the new one at every timestep, hence the system is stable so long as $|A| \leq 1$. (This is consistent with our physical understanding of waves in homogeneous media; for plane waves the amplitude will stay constant, while for all other waves the amplitude will decay.) Substituting this equality into Eq. (2.31) leads to the characteristic quadratic equation

$$A^2 + (b^2 - 2)A + 1 = 0 \quad , \tag{2.32}$$

for which the two solutions are

$$A_{1,2} = \frac{-(b^2-2) \pm \sqrt{(b^2-2)^2 - 4}}{2} \quad . \tag{2.33}$$

It can be shown that $|A| \leq 1$ when $|b| \leq 2$. In other words, the numerical model used in k-Wave is stable when

$$|k\kappa\Delta t c_0| \leq 2 \quad \text{for all } k \quad . \tag{2.34}$$

For a pseudospectral time domain model $\kappa = 1$, so the stability criterion is simply $k_{\max}\Delta t c_0 \leq 2$. For the k-space method $\kappa = \operatorname{sinc}(c_{\text{ref}} k\Delta t/2)$ and so the stability criterion becomes

$$|\sin(c_{\text{ref}} k\Delta t/2)| \leq \frac{c_{\text{ref}}}{c_0}. \tag{2.35}$$

In a homogeneous medium the k-space method can be made unconditionally stable (and exact) by choosing $c_{\text{ref}} = c_0$, as sine is never greater than 1.

It is interesting to note that if c_{ref} is chosen so that $(c_{\text{ref}}/c_0) > 1$ then the model will also be unconditionally stable, but the k-space operator κ will now no longer correct the phase exactly, so phase errors will accumulate. As shown in Fig. 2.4, the larger c_{ref}/c_0 is than 1, the greater the phase error will be, and it will grow until the solution is completely

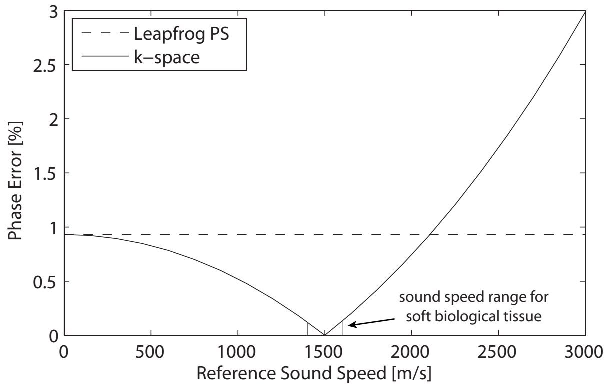

Figure 2.4: Phase error in the propagation of a plane wave after 50 wavelengths against the reference sound speed c_{ref} used in the k-space operator κ for $c_0 = 1500$ m/s [2].

corrupted. So with this choice of c_{ref}, the model is stable (the solution doesn't "blow up") but it is not necessarily accurate.

The remaining option is to choose c_{ref} such that $(c_{\text{ref}}/c_0) < 1$. In this case, the phase errors are guaranteed to be smaller than in the pseudospectral case (the k-space model becomes the pseudospectral model as $c_{\text{ref}} \to 0$ because $\kappa \to 1$), but the model is now only conditionally stable. The criterion for stability is given by

$$\Delta t \leq \frac{2}{c_{\text{ref}} k_{\max}} \sin^{-1}\left(\frac{c_{\text{ref}}}{c_0}\right), \tag{2.36}$$

(Note, here $k_{\max} =$ `max(kgrid.k(:))`). This discussion of the homogeneous case suggests that in the heterogeneous case, when $c_0 = c_0(x)$, there are two options: (1) if the reference sound speed in κ is chosen to be $c_{\text{ref}} = \max(c_0(x))$ then stability is ensured but the timestep must be small enough to ensure the phase error does not corrupt the solution, or (2) if $c_{\text{ref}} = \min(c_0(x))$ is chosen then the phase error is necessarily bounded but the timestep must be small enough to ensure stability. The criterion for this is:

$$\Delta t \leq \frac{2}{c_{\text{ref}} k_{\max}} \sin^{-1}\left(\frac{c_{\text{ref}}}{\max(c_0)}\right). \tag{2.37}$$

A stability analysis for the nonlinear, absorbing model does not lead to such succinct results as these. However, in general, absorption will act to improve the accuracy of the numerical solution as it dampens the high frequencies introduced by the nonlinearity.

A number that is useful when discussing stability is the one-dimensional Courant-Friedrichs-Lewy (CFL) number, which is defined as the ratio of the distance a wave can travel in one time step to the grid spacing:

$$\text{CFL} \equiv c_0 \Delta t / \Delta x \ . \tag{2.38}$$

The CFL number could be thought of as a non-dimensionalised time step, and for that reason it is useful for defining the maximum permissible time step without reference to a specific grid spacing. Note, care must be exercised when comparing particular values for the CFL stability condition between different types of numerical models (e.g., between pseudospectral and finite difference models) as the CFL number is dependent on the grid

spacing. As an example, a value of CFL = 0.3 in a pseudospectral model with 2 grid points per wavelength will equate to a time step 5 times larger than a finite difference model with 10 grid points per wavelength and the same CFL number. Using the definition of the CFL number, Eq. (2.34) can be rewritten as $|\kappa|\mathrm{CFL} \leq 2/\pi$ because $k_{\max}\Delta x = \pi$. Similarly Eq. (2.36) then becomes

$$\mathrm{CFL} \leq \frac{2}{\pi}\left(\frac{c_0}{c_{\mathrm{ref}}}\right)\sin^{-1}\left(\frac{c_{\mathrm{ref}}}{c_0}\right). \tag{2.39}$$

Within k-Wave, the discrete equations in Sec. 2.4 are iteratively solved using a time step based on the CFL number given by the user. The size of the time step is calculated using the formula

$$\Delta t = \frac{\mathrm{CFL}\Delta x}{c_{\max}} \ , \tag{2.40}$$

where $c_{\max}$ is the maximum value of the sound speed in the medium. A CFL number of 0.3 (which is the default value used in the function `makeTime`) typically provides a good balance between accuracy and computational speed for weakly heterogeneous media [27, 36, 2].

With questions (1) and (2) answered, we can be confident that the numerical model is stable and is compatible with the continuous governing equations. However, we still haven't directly answered question (3). How can we be sure that the results are accurate, i.e., the solution calculated from the discrete equations coincides with the solution to the continuous equations? This is essentially a matter of ensuring that the spatial discretisation Δx and the temporal discretisation Δt are small enough for the problem being studied. This is expressed formally in Lax's Equivalence Theorem, which says that a consistent, stable numerical scheme is convergent [52]. This means the numerical solution will converge to the solution of the continuous equations as Δt and $\Delta x \to 0$. In practice, there will be a limit to how small Δx and Δt can be due to the available computing resources. However, this just means there is a limit to the highest frequency that can be modelled. When setting up a simulation it is necessary to ensure that the grid spacing is sufficiently small that the highest frequency of interest can be supported by the grid. The issues of discretisation and frequency content are discussed further in Sec. 3.4.

In general, the choice of the timestep will be governed by several considerations. In the homogeneous case, the model will give accurate results for any timestep, but if a time varying output that contains all the frequencies that the grid can support is required, the timestep must satisfy $\Delta t \leq \Delta x/c_{\max}$, which is the same as saying CFL $\leq (c_0/c_{\max})$. In the heterogeneous case, Δt (or equivalently the CFL number) must not only be chosen small enough for stability, but may need to be even smaller to achieve sufficient accuracy. The principal reason is that decreasing Δt improves the accuracy with which propagation across interfaces between media of different properties are dealt with. Because the discrete system of equations is consistent with the continuous governing equations, there is a simple procedure to ensure the results from the model are accurate: repeat the simulations with decreasing values of Δt until the results do not change significantly within the frequency range of interest. In heterogeneous examples, lower frequencies, which are represented by more points per wavelength on the grid, will typically be modelled more accurately than higher frequencies.

2.8 Smoothing and the Band-Limited Interpolant

The application of the discretised equations discussed in Sec. 2.4 for particular discrete initial conditions can result in oscillations in the numerical solution for the pressure field that are not intuitively expected. These oscillations are a purely numerical effect resulting from the use of the Fourier pseudospectral method, and are not evidence of an instability. They arise because the Fourier collocation spectral method uses an FFT of finite length to calculate spatial gradients, so the field parameters are implicitly represented using a truncated Fourier series. The Fourier coefficients $P(k_m)$ are chosen so that the continuous function $\hat{p}(x)$ given by

$$\hat{p}(x) = \frac{1}{N_x} \sum_{m=-N_x/2}^{N_x/2-1} P(k_m) e^{-\frac{2\pi i}{N_x}\frac{mx}{\Delta x}} \ , \tag{2.41}$$

matches the discretised function $p(x_j)$ at the grid points $x = x_j$. (Matching at a discrete set of points is the defining feature of a collocation method.) The continuous function, $\hat{p}(x)$, is called the band-limited interpolant as it interpolates between the discrete set of grid points x_j using a finite set of Fourier components [53]. It is constructed using the FFT coefficients at the discrete spatial frequencies k_m, where

$$P(k_m) = \sum_{j=-N_x/2}^{N_x/2-1} p(x_j) e^{\frac{2\pi i}{N_x} mj} \ . \tag{2.42}$$

There are two aspects which are key to understanding how this might lead to oscillations appearing in the solution, unless sufficient care is taken. The first is recognising that while $\hat{p}(x)$ may match $p(x_j)$ at the points $x = x_j$, there is no guarantee about how $\hat{p}(x)$ behaves in between these points. If there are large jumps in $p(x_j)$ between adjacent points, i.e., if $p(x_j) - p(x_{j-1})$ is large, then $\hat{p}(x)$ might have to oscillate in between points x_{j-1} and x_j in order to reach $p(x_j)$. The second is realising that it is the band-limited interpolant $\hat{p}$ and not $p(x_j)$ that is propagated during the simulation. Consequently, when $\hat{p}$ is resampled at the discrete grid points x_j at a later timestep, oscillations can appear in the solution. An example of this is shown in Fig. 2.5, where the discrete pressure is shown with a stem plot, and the underlying band limited interpolant is shown as a solid line [14].

If desired, it is possible to reduce the visible oscillations in the solution by making $p(x_j)$ smoother, i.e., by reducing the size of the jumps between consecutive grid points. This is equivalent to reducing the amplitudes of the higher spatial frequency components $P(k_m)$. This is done automatically within the simulation functions when an initial pressure distribution is defined (i.e., `source.p0`) using the k-Wave function `smooth`. This function applies a Blackman window in the spatial frequency domain to reduce the amplitude of the higher spatial frequencies. (The analogy in the purely continuous case is the link between the smoothness of a function and the rate of decay of its Fourier transform. A very sharp function, for example a delta function, has a flat frequency spectrum, whereas the Fourier transform of an analytic function decays very quickly. In between these extremes, the more continuous derivatives that a function has, the more quickly its Fourier transform decays.)

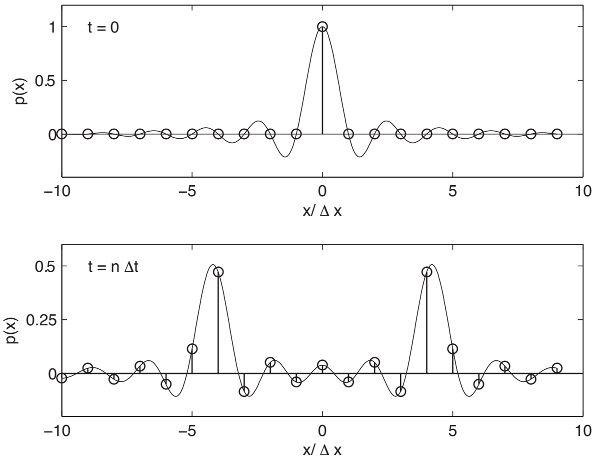

Figure 2.5: Propagation of an initial pressure distribution set to a discrete spatial delta function. Oscillations appear in the solution at $t = n\Delta t$. The discrete pressure distribution is shown with a stem plot, while the band-limited interpolant is shown with a solid line.

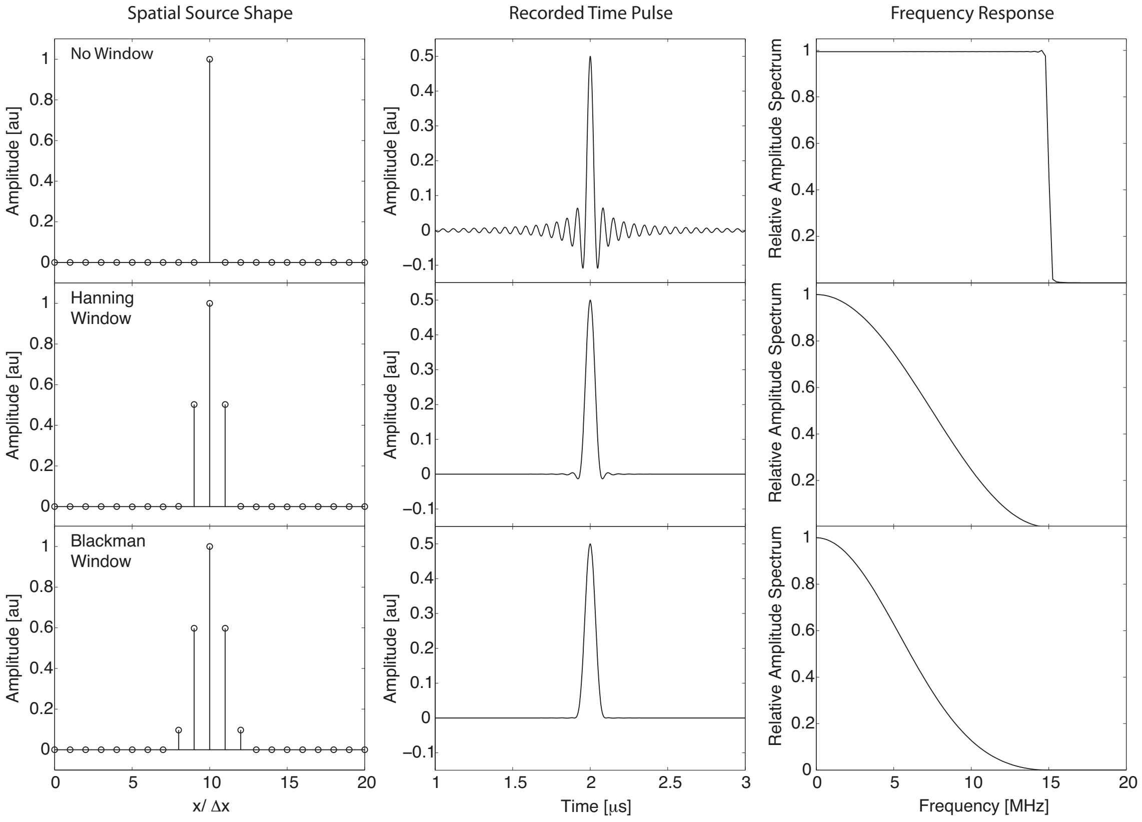

Figure 2.6: Propagation of an initial pressure distribution set to a discrete delta function. If no window is used, oscillations appear in the recorded pressure signal because of the properties of the underlying band-limited interpolant. These oscillations can be reduced by windowing the initial pressure distribution in the spatial frequency domain before the simulation begins [14].

Selecting the most appropriate window or function to force the Fourier coefficients to decay requires a trade off between the level of smoothing and the level of observable oscillations. From a signal processing perspective, the amount of smoothing is related to the main lobe width of the window, while the level of oscillations is related to the side lobe levels. Figure 2.6 illustrates the effect of smoothing a delta function initial pressure distribution using Hanning and Blackman windows. In both cases, the magnitude of the pressure distribution has been corrected by the coherent gain of the window. Note, the default smoothing behaviour used by the simulation functions can be modified using the optional input parameter `'smooth'` (see discussion in Sec. 3.6).

Chapter 3

First-Order Simulation Functions

3.1 Overview

There are three simulation functions in the k-Wave Toolbox that implement the first-order k-space model for fluid media described in the previous chapter. These are named `kspaceFirstOrder1D`, `kspaceFirstOrder2D`, and `kspaceFirstOrder3D` and correspond to simulating wave propagation in one, two, and three dimensions as their names imply. In this case, "first-order" refers to the fact we are solving a system of coupled first-order partial differential equations. It's not related to the order of numerical accuracy of the solution, or to the order of the acoustic variables retained in the governing equations.

The simulation functions are called with four input structures; `kgrid`, `medium`, `source`, and `sensor`. The properties of the simulation are then set as fields for these structures in the form `structure.field`. The four structures respectively define the properties of the computational grid, the material properties of the medium, the properties and locations of any acoustic sources, and the properties and locations of the sensor points used to record the evolution of the pressure and particle velocity fields over time. When the simulation functions are called, the propagation of the wave-field in the medium is then computed step by step, with the acoustic field at the sensor elements stored after each iteration. These values are returned when the time loop has completed.

To illustrate the general structure of the MATLAB code required, a simple example of using k-Wave to model an initial value problem in 2D is shown below. In this example, the domain is divided into 128 by 256 grid points with a grid point spacing of 50 μm. The sound speed is set to be heterogeneous, with a layer of higher speed near the top of the domain. The source is set to be an initial pressure distribution in the shape of a disc, and the sensor is set to be a circular array with 50 sensor points. The four input structures are passed to `kspaceFirstOrder2D` which then calculates and returns the acoustic pressure recorded at each sensor point for each time step.

During the simulation, a visualisation of the propagating wave-field and a status bar are displayed, with frame updates every ten time steps. A snapshot of a 2D simulation of a

focused ultrasound pulse is shown in Fig. 3.1(b). The k-Wave color map displays positive pressures as yellows to reds to black, and negative pressures as light to dark blue-greys. The default plot scale is set to display values from -1 to 1, with zero displayed as white. Most of the default plot settings can be modified using optional input parameters as described in Sec. 3.6.

```
% create the computational grid
Nx = 128;      % number of grid points in the x (row) direction
Ny = 256;      % number of grid points in the y (column) direction
dx = 50e-6;    % grid point spacing in the x direction [m]
dy = 50e-6;    % grid point spacing in the y direction [m]
kgrid = makeGrid(Nx, dx, Ny, dy);

% define the medium properties
medium.sound_speed = 1500*ones(Nx, Ny); % [m/s]
medium.sound_speed(1:50, :) = 1800;     % [m/s]
medium.density = 1040;                  % [kg/m^3]

% define an initial pressure using makeDisc
disc_x_pos = 75;                        % [grid points]
disc_y_pos = 120;                       % [grid points]
disc_radius = 8;                        % [grid points]
disc_mag = 3;                           % [Pa]
source.p0 = disc_mag*makeDisc(Nx, Ny, disc_x_pos, disc_y_pos, disc_radius);

% define a Cartesian sensor mask of a centered circle with 50 sensor elements
sensor_radius = 2.5e-3;                 % [m]
num_sensor_points = 50;
sensor.mask = makeCartCircle(sensor_radius, num_sensor_points);

% run the simulation
sensor_data = kspaceFirstOrder2D(kgrid, medium, source, sensor);
```

A detailed discussion of each of the four input structures is given in the following sections, with a graphical view given in Fig. 3.1(a) for reference. There are also a large number of worked examples included with the toolbox. These can be accessed through the MATLAB documentation as described in Sec. 1.4.

3.2 Defining the Computational Grid

The first input `kgrid` defines the properties of the computational grid. This determines how the continuous medium is divided up into a evenly distributed mesh of grid points (the terms grid *points* and grid *nodes* are used here interchangeably). The grid points represent the discrete positions in space at which the governing equations are solved. This particular input must be created using the function `makeGrid`, which automatically creates and populates the required fields. The syntax for creating a computational grid in 1D,

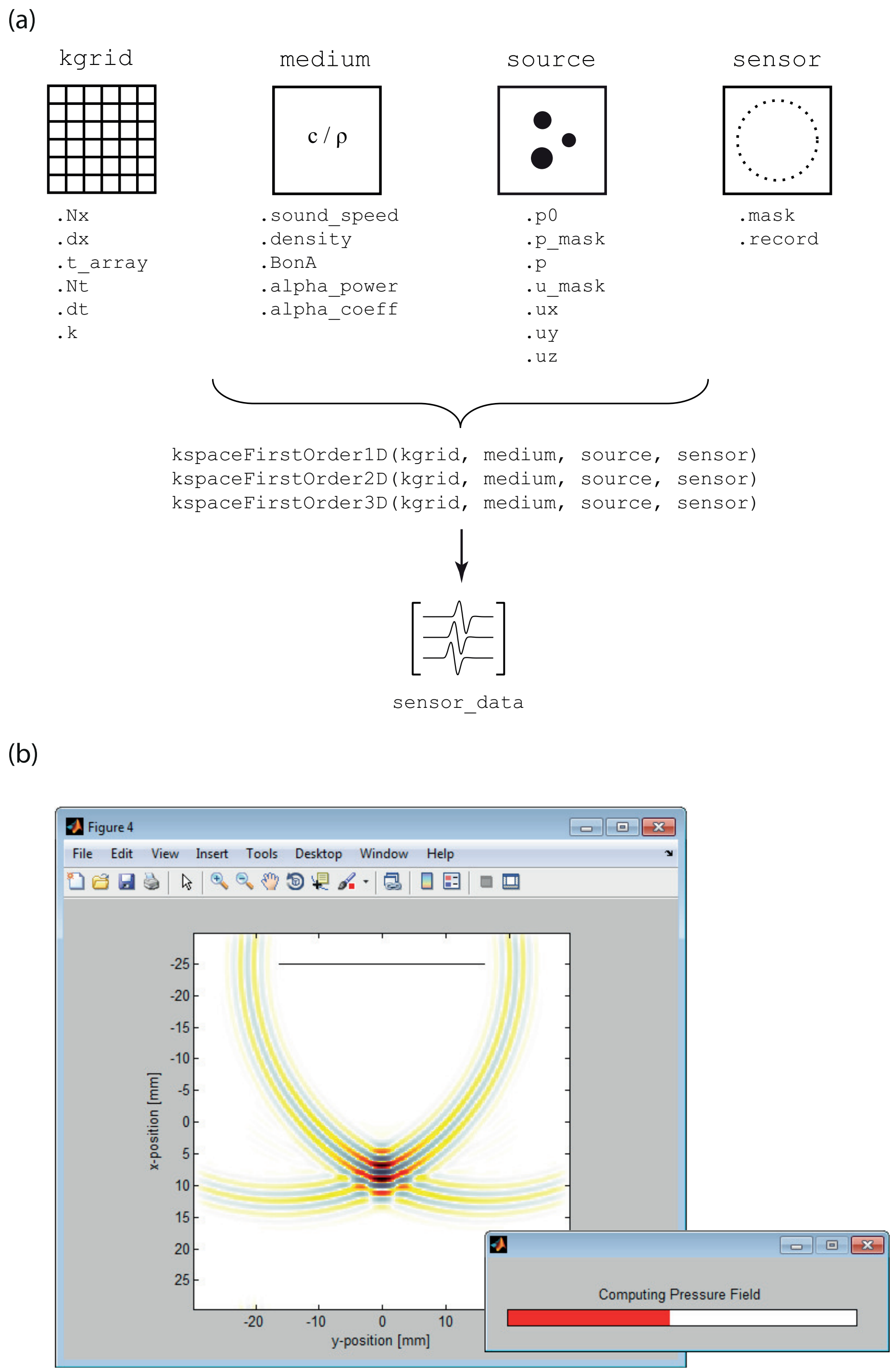

Figure 3.1: (a) Overview of the four inputs structures and the main input fields used for the first-order simulation functions in k-Wave. (b) Snapshot of a 2D simulation of a focused pulse using k-Wave. The source mask is shown as black line, and the progress of the simulation is illustrated by the status bar. The anisotropic absorption within the perfectly matched layer (PML) around the outside of the domain is also visible.

2D, and 3D is shown below. k-Wave uses the convention that 1D variables are stored and indexed as `(x, 1)`, 2D variables as `(x, y)`, and 3D variables as `(x, y, z)`.

```
% create computational grid for a 1D simulation
kgrid = makeGrid(Nx, dx);

% create computational grid for a 2D simulation
kgrid = makeGrid(Nx, dx, Ny, dy);

% create computational grid for a 3D simulation
kgrid = makeGrid(Nx, dx, Ny, dy, Nz, dz);
```

The function `makeGrid` takes pairs of inputs corresponding to the number of grid points (`Nx`, `Ny`, and `Nz`) and the grid point spacing (`dx`, `dy`, and `dz`) in each Cartesian direction. Within `makeGrid`, these variables are used to create matrices of the wavenumbers and Cartesian grid coordinates. An object of the `kWaveGrid` class (called `kgrid` in the examples shown above) is then returned. This object has a number of properties which are used by the simulation and utility functions within k-Wave. A list of these properties is given in Table 3.1. For example, `kgrid.x_size` returns the total size of the computational grid in the x-direction in metres, where `kgrid.x_size = kgrid.Nx * kgrid.dx`. An object orientated approach for defining `kgrid` is used to enable many of the matrices to be created on the fly, rather than being stored in memory.

The discrete wavenumber vectors `kgrid.kx_vec`, `kgrid.ky_vec`, and `kgrid.kz_vec` are defined according to Eq. (2.17e) based on the values for `Nx`, `Ny`, `Nz` and `dx`, `dy`, `dz`. The wavenumbers are used to calculate the spatial gradients of the acoustic field parameters using the Fourier collocation spectral method as described in Sec. 2.4. The maximum spatial frequency that can be represented by a particular computational grid is given by the Nyquist limit of two grid points per wavelength, where `kx_max = pi/dx`. The spatial wavenumber and temporal frequency are related by $k = 2\pi f/c_0$, thus the maximum wavenumber corresponds to a maximum temporal frequency of `f_max = min(c_0)/(2*dx)`. If the grid spacing is not uniform in each Cartesian direction, the maximum frequency supported in all directions will be dictated by the largest grid spacing.

When creating a new simulation, the easiest way to select appropriate values for `Nx` and `dx` (etc) is to start with the desired domain size in metres and the maximum desired frequency in Hz. The required grid spacing and number of grid points can then be calculated. For example:

```
% compute dx and Nx based on a desired x_size and f_max
points_per_wavelength = 3;
dx = c0_min/(points_per_wavelength*f_max);
Nx = round(x_size/dx);
```

Here `c0_min` is the minimum sound speed in the medium, and `points_per_wavelength` is the desired number of points per spatial wavelength at the maximum frequency of interest. For linear simulations in homogeneous media, simulations can be run using close to the Nyquist limit of two points per wavelength (at least three points per wavelength is recommended for the PML to work effectively [51]). However, for heterogeneous media, using four or more points per wavelength is recommended if accurate reflection coefficients

Table 3.1: Properties of the `kWaveGrid` object returned by `makeGrid`. The second group of properties are repeated for each spatial dimension `x`, `y`, `z`. For 1D and 2D grids, the unused properties for `y` and `z` are set to zero.

Fieldname	Description
`kgrid.k`	plaid ND grid of the scalar wavenumber
`kgrid.k_max`	maximum spatial frequency supported by the grid
`kgrid.t_array`	evenly spaced array of time values
`kgrid.Nt`	number of time steps
`kgrid.dt`	time step
`kgrid.dim`	number of spatial dimensions (1, 2, or 3)
`kgrid.total_grid_points`	total number of grid points
`kgrid.Nx`	number of grid points
`kgrid.dx`	grid point spacing [m]
`kgrid.x`	plaid ND grid of the x coordinate centred about 0 [m]
`kgrid.x_vec`	1D vector of the x coordinate [m]
`kgrid.x_size`	length of grid dimension [m]
`kgrid.kx`	plaid ND grid of the x-direction wavenumbers
`kgrid.kx_vec`	1D vector of the x-direction wavenumbers
`kgrid.kx_max`	maximum spatial frequency in the x-direction

close to the maximum frequency are required [26, 27, 36, 2]. For nonlinear simulations, the maximum frequency should be set to the frequency of the highest harmonic that has significant energy [2].

The spatial gradient calculations used in k-Wave make heavy use of the fast Fourier transform (FFT). Depending on the complexity of the simulation, up to fourteen FFTs are calculated for each time step. The time to compute each FFT can be minimised by choosing the total number of grid points in each direction (including the PML) to be a power of two, or to have small prime factors. In many cases, the performance of k-Wave can be improved by slightly modifying the values of `kgrid.Nx`, `kgrid.Ny` (etc) so that the largest prime factor is small. Appropriate values to choose for any given range can be obtained using the function `checkFactors`. This returns the numbers within the specified range that have maximum prime factors of seven or less. An example of finding good grid sizes to choose between 100 and 150 is shown below.

```
>> checkFactors(100, 150)
Numbers with a maximum prime factor of 2
128
Numbers with a maximum prime factor of 3
108  144
Numbers with a maximum prime factor of 5
100  120  125  135  150
Numbers with a maximum prime factor of 7
105  112  126  140  147
```

Using grid sizes of large prime numbers (for example 149) should be avoided if possible. For more information on the performance and implementation of the FFT library used by MATLAB, see FFTW [54].

When the acoustic waves reach the edge of the computational domain, they are absorbed by a special type of anisotropic absorbing boundary layer known as a perfectly matched layer or PML (see discussion in Sec. 2.6). The effects of the layer can be seen by running one of the examples included in the toolbox and watching what happens to the propagating waves as they get close to the edge of the computational domain. By default, the PML occupies a strip of 20 grid points (10 grid points in 3D) around the edge of the domain. It is important to note that the PML is placed *inside* the grid size specified using `makeGrid`. This means users must be careful not to place the source or sensor points inside this layer. Alternatively, the PML can be set to be *outside* the grid size defined by the user by setting the optional input parameter `'PMLInside'` to `false` (see Sec. 3.6 for an overview of how optional input parameters are used). In this case, the grid size and medium inputs are automatically enlarged before the simulation begins.

After the `kWaveGrid` object has been created, the only parameter that can be modified by the user is `kgrid.t_array`. This describes the array of time values over which the simulation is run, and is set to `'auto'` by default. In this case, the time array is automatically calculated within the simulation functions using `makeTime`. This function sets the total time to the time it would take for an acoustic wave to travel across the longest grid diagonal at the minimum sound speed. The time step is based on a Courant-Friedrichs-Lewy (CFL) number of 0.3 and the maximum sound speed in the medium, where `kgrid.dt = CFL*dx_min/c0_max` (see discussion in Sec. 2.7). The time array can also be set manually, either by calling `makeTime`, or by setting the time array explicitly. Several examples are given below. The time array must be evenly spaced and monotonically increasing. After creation, the number of time points and the size of the time step can be queried using `kgrid.Nt` and `kgrid.dt`.

```
% create the time array using makeTime setting the CFL and end time
kgrid.t_array = makeTime(kgrid, medium.sound_speed, CFL, t_end);

% create the time array using makeTime setting the end time
kgrid.t_array = makeTime(kgrid, medium.sound_speed, [], t_end);

% create the time array explicitly
kgrid.t_array = 0:1e-9:1e-6;
```

3.3 Defining the Acoustic Medium

The second input structure `medium` defines the material properties of the medium at each grid point. There are five material properties that can be defined; the isentropic sound speed (`medium.sound_speed`), the ambient mass density (`medium.density`), the nonlinearity parameter (`medium.BonA`), the power law absorption coefficient or prefactor (`medium.alpha_coeff`), and the power law absorption exponent (`medium.alpha_power`). A summary is given in Table 3.2. Except for the power law absorption exponent (which

Table 3.2: Properties of the `medium` input structure. The parameter `medium.sound_speed` must be defined for all simulations. The parameter `medium.density` can be omitted for linear simulations in homogeneous and lossless media, otherwise it must also be defined. All other fields are optional. If the nonlinearity or absorption parameters are not set, the simulation is assumed to be linear and lossless.

Fieldname	Description
`medium.sound_speed`	sound speed distribution within the medium [m/s]
`medium.density`	ambient density distribution within the medium [kg/m^3]
`medium.BonA`	nonlinearity parameter
`medium.alpha_coeff`	power law absorption prefactor [dB/(MHz^{y}cm)]
`medium.alpha_power`	power law absorption exponent
`medium.sound_speed_ref`	reference sound speed used in the k-space operator [m/s]
`medium.alpha_mode`	optional input to force either the absorption or dispersion terms in the equation of state to be excluded; valid inputs are `'no_absorption'` or `'no_dispersion'`
`medium.alpha_sign`	two element array used to control the sign of absorption and dispersion terms in the pressure-density relation
`medium.alpha_filter`	frequency domain filter applied to the absorption and dispersion terms in the pressure-density relation

must be a scalar), each of the material properties can be defined as either a scalar (if the medium property is homogeneous), or a matrix (if the medium property is heterogeneous). If the parameters are heterogeneous, the input matrix must be the same size as the computational grid, for example:

```
% create a heterogeneous sound speed for a layered medium in 3D
medium.sound_speed = 1500*ones(kgrid.Nx, kgrid.Ny, kgrid.Nz);
medium.sound_speed(1:layer_size, :, :) = 1600;
```

The parameter `medium.sound_speed` must be defined by the user for all simulations. The parameter `medium.density` can be omitted for linear simulations in homogeneous and lossless media, otherwise it must also be defined. The remaining medium fields are all optional. If the nonlinearity parameter `medium.BonA` is not set, the simulation is assumed to be linear, and linear governing equations are solved. Similarly, if the absorption parameters `medium.alpha_coeff` and `medium.alpha_power` are not set, the simulation is assumed to be lossless.

The absorption parameters correspond to modelling power law absorption of the form $\alpha = \alpha_0 f^y$, where $\alpha_0 \equiv$ `medium.alpha_coeff` and $y \equiv$ `medium.alpha_power`. The parameter for `medium.alpha_coeff` must be given in units of dB MHz^{-y} cm^{-1}. It is possible to convert to and from units of Np (rad/s)$^{-y}$ m^{-1} using the functions `neper2db` and `db2neper`. The input for the power law absorption exponent `medium.alpha_power` must be between 0 and 3 and not equal to 1. This restriction is due to the way the disper-

sion term is derived via the Kramers-Kronig relations (η in Eq. 2.6 has a singularity for $y = 1$). If modelling power law absorption with `medium.alpha_power = 1` is required and modelling dispersion is not important, the dispersion term can be switched off by setting `medium.alpha_mode = 'no_dispersion'`. Alternatively, a value of `medium.alpha_power` close to 1 can be used (for example 1.05).

There are no other restrictions on the values for the material parameter inputs, except that they must be real numbers. For example, the sound speed and density at each grid point could be derived from a CT scan using the function `hounsfield2density`, or they could be loaded from an image using `loadImage`.

In addition to the material definitions, a number of control parameters can also be set by the user. In particular, the reference sound speed c_{ref} used within the k-space operator κ in Eq. (2.16) can be defined using `medium.sound_speed_ref`. If this is not set, it defaults to the maximum sound speed within the medium (see discussion in Sec. 2.7). The remaining absorption parameters shown in Table 3.2 allow the absorption and dispersion terms within the pressure-density relation to be filtered or reversed. These parameters are primarily used for photoacoustic image reconstruction where the absorption term is reversed during the reconstruction to compensate for the effects of acoustic absorption in the experimental data [55, 56].

3.4 Defining the Acoustic Source Terms

The third input structure `source` defines the properties and location of any acoustic sources in the medium. There are three different types of source that can be used. The first is an initial pressure distribution. This source type is usually the most appropriate for users wanting to simulate pulsed photoacoustic or thermoacoustic tomography [3]. Within k-Wave, an initial pressure distribution is set by assigning a matrix to `source.p0`. There are no restrictions on `source.p0`, except that it must be the same size as the computational grid, and the values must be real. Several functions are included in the toolbox for the creation of simple geometric shapes, for example, `makeDisc` and `makeCircle` in 2D, and `makeBall` and `makeSphere` in 3D. An example of setting the initial pressure distribution to a ball centred within a 3D grid is shown below.

```
% define an initial pressure distribution using makeBall
source.p0 = makeBall(Nx, Ny, Nz, Nx/2, Ny/2, Nz/2, radius);
```

By default, `source.p0` is spatially smoothed within the simulation functions using `smooth` before the simulation begins (the reason behind this is discussed in more detail in Sec. 2.8). The default smoothing behaviour can be modified using the optional input parameter `'smooth'` (see Sec. 3.6).

The second type of source that can be defined in k-Wave is a time varying pressure source. This physically corresponds to a mass source, and appears as a source term in the mass conservation equation (see discussion in Secs. 2.2 and 2.4). This type of source requires two parameters; a source mask that defines which grid points belong to the source, and the actual time varying pressure input. The source mask is defined by assigning a binary matrix (i.e., a matrix of 1's and 0's) to `source.p_mask`. This must be the same size as

the computational grid. The 1's within the matrix represent the grid points within the domain that form part of the source. The time varying input signal is then assigned to `source.p` which is indexed as `(source_point_index, time_index)`. The input signal can be defined either as a single time series (in which case the same time series is applied to all of the source points), or a matrix of time series following the source points using MATLAB's column-wise linear matrix index ordering. For example, if `source.p_mask` is defined as

```
source.p_mask =
     0     1     0
     1     0     1
     1     0     1
     0     1     0
```

then a matrix for `source.p(source_point_index, time_index)` would have six rows, where the time series in each row correspond to the source points within the grid in the following order

```
     0     3     0
     1     0     5
     2     0     6
     0     4     0
```

In 3D, the matrices are indexed first in the x-direction (dimension 1), then the y-direction (dimension 2), and then the z-direction (dimension 3). This indexing follows the order that the matrix elements are physically stored in memory and on disk. (MATLAB uses column-major order to store multidimensional arrays. This is different to C/C++ and other languages which use row-major order.) The column-major matrix ordering can be viewed in MATLAB by typing `>> reshape(1:Nx*Ny*Nz, Nx, Ny, Nz)`. Note, the source can have any number of time points—it doesn't need to be the same length as `kgrid.t_array`.

The third type of source that can be defined is a time varying particle velocity source. This physically corresponds to a force source, and appears as a source term in the momentum conservation equation (see discussion in Secs. 2.2 and 2.4). This is defined in an analogous fashion to a time varying pressure source. A binary matrix (i.e., a matrix of 1's and 0's) is assigned to `source.u_mask` where the 1's represent the grid points that form part of the source. The time varying input signal is then assigned to `source.ux`, `source.uy`, and `source.uz`. These can be defined independently as required, and may be a single time series (in which case the same time series is applied to all of the source points), or a matrix of time series following the source points using MATLAB's column-wise linear index ordering. An example of creating a single time series using `toneBurst` and assigning it to the particle velocity in the x-direction within a 2D simulation is shown below.

```
% define the source mask to be a line across the top of the grid
source.u_mask = zeros(Nx, Ny);
source.u_mask(1, :) = 1;
```

Table 3.3: Properties of the `source` input structure. Pressure inputs are given in units of Pa, while velocity inputs are given in units of $\mathrm{m\,s^{-1}}$.

Fieldname	Description
`source.p0`	initial pressure distribution
`source.p`	time varying pressure at each of the source positions given by `source.p_mask`
`source.p_mask`	binary matrix specifying the positions of the time varying pressure source distribution
`source.p_mode`	optional input to control whether the input pressure is injected as a mass source or enforced as a dirichlet boundary condition; valid inputs are `'additive'` (the default) or `'dirichlet'`
`source.ux`	time varying particle velocity in the x-direction at each of the source positions given by `source.u_mask`
`source.uy`	time varying particle velocity in the y-direction at each of the source positions given by `source.u_mask`
`source.uz`	time varying particle velocity in the z-direction at each of the source positions given by `source.u_mask`
`source.u_mask`	binary matrix specifying the positions of the time varying particle velocity distribution
`source.u_mode`	optional input to control whether the input velocity is applied as a force source or enforced as a dirichlet boundary condition; valid inputs are `'additive'` (the default) or `'dirichlet'`

```
% define a tone burst and assign it to the x-direction particle velocity
sampling_freq = 1/dt;          % [Hz]
tone_burst_freq = 2e5;         % [Hz]
tone_burst_cycles = 3;
source.ux = toneBurst(sampling_freq, tone_burst_freq, tone_burst_cycles);
```

The temporal sampling frequency of the input and output signals is dictated by the size of the time step, `kgrid.dt`. This means the highest frequency that can be represented in a time varying pressure or velocity input is the Nyquist limit of `1/(2*kgrid.dt)`. However, the highest temporal frequency that can be represented on the spatial grid is given by the Nyquist limit of `c0/(2*dx)` or `CFL/(2*kgrid.dt)`. For most simulations, the CFL number will be less than 1 (`makeTime` uses a CFL of 0.3 by default). This means it is possible to define time varying pressure or velocity input signals that contain frequencies that cannot be represented on the spatial grid. This can cause large unwanted errors in the simulation, so care must be taken that maximum frequency supported by the grid is not exceeded! This frequency is reported on the command line at the beginning of each simulation and can be easily calculated using the expressions given above. Input signals can also be automatically restricted to the range of supported frequencies by using the function `filterTimeSeries`. This applies a finite impulse response (FIR) filter designed using the Kaiser windowing method. The filter can be set to either zero or linear phase as required.

By default, the time varying pressure and velocity sources are added to the medium as the injection of mass or force. It is also possible to enforce these values using a Dirichlet-type boundary condition (although they can be enforced anywhere within the domain, not just at the boundary). This is achieved by setting `source.p_mode` and `source.u_mode` to `'dirichlet'`. In this case, at each time step, the input pressure and velocity values are used to replace the existing values at the grid points specified by `source.p_mask` and `source.u_mask`, rather than adding to them. This is useful for enforcing known or measured values within a simulation. For example, the time varying pressure-field measured in a 2D plane by a hydrophone, or the surface pressure values measured in a photoacoustic experiment. A summary of the source input fields is given in Table 3.3.

3.5 Defining the Sensor

The final input structure `sensor` defines the properties and location of the sensor points used to record the acoustic field at each time step during the simulation. The position of the sensor points within the computational domain is set using `sensor.mask`. This can be defined in three different ways: (1) as a binary matrix which directly specifies the grid points that record the data, (2) as the grid coordinates of two opposing corners of a line (in 1D), rectangle (in 2D), or cuboid (in 3D) of grid points that record the data, or (3) as a set of Cartesian coordinates.

A binary sensor mask is defined by assigning a binary matrix the same size as the computational grid to `sensor.mask`, where the 1's represent the grid points within the domain that form part of the sensor. Two examples of creating a binary sensor mask in 2D are given below.

```
% define a 2D binary sensor mask in the shape of a line
x_offset = 25;                  % [grid points]
width = 50;                     % [grid points]
sensor.mask = zeros(Nx, Ny);
sensor.mask(x_offset, Ny/2 - width/2 + 1:Ny/2 + width/2) = 1;

% define a 2D binary sensor mask in the shape of an arc using makeCircle
x_pos = Nx/2;                   % [grid points]
y_pos = Ny/2;                   % [grid points]
radius = 20;                    % [grid points]
arc_angle = pi/2;               % [radians]
sensor.mask = makeCircle(Nx, Ny, x_pos, y_pos, radius, arc_angle);
```

For regular shaped binary sensor masks (defined by a uniform prismatic polytope), an alternate way to specify the position of the sensor points is to define two opposing corners of the sensor region. These are specified as column vectors in the form `[X1; X2]` in 1D, `[X1; Y1; X2; Y2]` in 2D, and `[X1; Y1; Z1; X2; Y2; Z2]` in 3D. The coordinates are given in units of grid points, where `(1, 1)` in 2D defines the upper left corner of the grid (similarly for other dimensions). Multiple sensor regions can be specified by adding additional column vectors to `sensor.mask` as shown below. For simulations using the C++ code (described in Sec. 4), using opposing corners can significantly reduce the size

of the input file, particularly for large simulations.

```
% define a rectangular sensor region in 2D by specifying the x, y location
% of two opposing corners of the rectangle
rect1_start = [25, 31];
rect1_end   = [30, 50];

% define a second rectangular sensor region
rect2_start = [71, 81];
rect2_end   = [80, 90];

% assign the list of opposing corners to the sensor mask
sensor.mask = [rect1_start, rect1_end; rect2_start, rect2_end].';
```

A Cartesian sensor mask is defined by assigning an $N \times M$ matrix of Cartesian coordinates to `sensor.mask`, where N is the number of dimensions (1, 2, or 3) and M is the number of sensor points. An example of creating a Cartesian sensor mask in 2D with 11 sensor points in a diagonal line is given below.

```
% define a 2D Cartesian sensor mask
x = -10:2:10;                   % [m]
y = -10:2:10;                   % [m]
sensor.mask = [x; y];
```

The Cartesian sensor points must always lie within the dimensions of the computational domain. The grid origin is in the centre, offset towards the end of the rows and columns if the number of grid points is even. The Cartesian coordinates of the grid points can be returned using `kgrid.x` and `kgrid.x_vec` (etc). An example of displaying the Cartesian distance of each grid point from the origin is shown below.

```
>> kgrid = makegrid(6, 1, 6, 1); sqrt(kgrid.x.^2 + kgrid.y.^2)

ans =

    4.2426    3.6056    3.1623    3.0000    3.1623    3.6056
    3.6056    2.8284    2.2361    2.0000    2.2361    2.8284
    3.1623    2.2361    1.4142    1.0000    1.4142    2.2361
    3.0000    2.0000    1.0000         0    1.0000    2.0000
    3.1623    2.2361    1.4142    1.0000    1.4142    2.2361
    3.6056    2.8284    2.2361    2.0000    2.2361    2.8284
```

If a Cartesian sensor mask is used, the values of the acoustic field at the sensor points are obtained at each time step using interpolation. By default, linear interpolation is used (this can be changed using the optional input parameter `'CartInterp'`; see discussion in Sec. 3.6). During the simulation, there is only a small performance difference between using Cartesian and binary sensor masks. However, the calculation of the triangulation points needed for interpolation when using a Cartesian mask can significantly lengthen the precomputation time, particularly for 3D simulations. A Cartesian sensor mask can be converted to a binary sensor mask (and vice versa) using the functions `cart2grid` and `grid2cart`. These functions are also useful for plotting Cartesian sensor masks using

imagesc. For example:

```
% plot a 2D source mask and Cartesian sensor mask using imagesc
imagesc(double(source.mask | cart2grid(kgrid, sensor.mask)));
```

After the four input structures have been defined, the simulation functions can be called. The syntax is identical for one, two, and three dimensional simulations. For example:

```
% run 3D simulation
sensor_data = kspaceFirstOrder3D(kgrid, medium, source, sensor);
```

At each time step during the simulation, the values of the acoustic pressure at the sensor points given in sensor.mask are stored. These values are returned after the simulation has completed. If using a binary or Cartesian sensor mask, the output is indexed as sensor_data(sensor_point_index, time_index). If the sensor mask is given as a binary matrix, the sensor data is ordered using MATLAB's column-wise linear index ordering. This is described in Sec. 3.4 in relation to the ordering of points within a binary source mask. If the sensor mask is given as a set of Cartesian coordinates, the computed sensor_data is returned in the same order in which the coordinates were defined.

If the sensor mask is instead defined using a list of opposing corners, the recorded data is indexed as:

```
sensor_data(region_index).p(x_index, time_index)
sensor_data(region_index).p(x_index, y_index, time_index)
sensor_data(region_index).p(x_index, y_index, z_index, time_index)
```

in 1D, 2D, and 3D, respectively. Here x_index, y_index, and z_index correspond to the grid index within the sensor region (e.g., line in 1D, rectangle in 2D, cuboid in 3D), and region_index corresponds to the number of the region if more than one is specified. The recorded data is numerically identical to that recorded using a binary sensor mask covering the same region.

It is possible to control the acoustic variables that are recorded by the sensor mask by setting the value of sensor.record. The desired field parameters are listed as strings within a cell array. For example, to record both the acoustic pressure and the particle velocity, sensor.record should be set to {'p', 'u'}. If a value for sensor.record is set, the output sensor_data returned from the simulation is defined as a structure, with the recorded acoustic variables appended as structure fields. For example, if sensor.record = {'p', 'p_max', 'u'}, then the individual output variables are accessed as sensor_data.p, sensor_data.p_max, sensor_data.ux, sensor_data.uy (etc). A full list of sensor options is given in Table 3.4.

Most of the acoustic parameters are recorded at each time step at the sensor points defined by sensor.mask. The outputs for these parameters are indexed as (sensor_point_index, time_index) in the same way as the acoustic pressure described above. The exceptions are the averaged quantities ('p_max', 'p_rms', 'u_max, 'p_rms, 'I_avg') and the quantities recorded over all the grid points within the domain ('p_max_all', 'p_min_all', 'p_final', 'u_max_all', 'u_min_all', 'u_final'). The averaged quantities return the maximum, average, or root-mean-squared values at each sensor point for the complete simulation and are indexed as (sensor_point_index). The quantities denoted _final and _all return the final, maximum, or minimum pressure and particle velocity fields over

Table 3.4: Properties of the `sensor` input structure.

Fieldname	Description
`sensor.mask`	binary grid, a set of Cartesian points, or a set of opposing corners specifying the positions where the pressure is recorded at each time-step
`sensor.record`	cell array of the acoustic parameters to record; valid inputs are:<br>`'p'` (acoustic pressure)<br>`'p_max'` (maximum pressure)<br>`'p_min'` (minimum pressure)<br>`'p_rms'` (RMS pressure)<br>`'p_final'` (final pressure field)<br>`'p_max_all'` (maximum pressure at all grid points)<br>`'p_min_all'` (minimum pressure at all grid points)<br>`'u'` (particle velocity)<br>`'u_max'` (maximum particle velocity)<br>`'u_min'` (minimum particle velocity)<br>`'u_rms'` (RMS particle velocity)<br>`'u_final'` (final particle velocity field)<br>`'u_max_all'` (maximum velocity at all grid points)<br>`'u_min_all'` (minimum velocity at all grid points)<br>`'u_non_staggered'`<br>(particle velocity on non-staggered grid points)<br>`'I'` (time varying acoustic intensity)<br>`'I_avg'` (average acoustic intensity)
`sensor.record_start_index`	time index at which the sensor should start recording (default = 1)
`sensor.time_reversal_boundary_data`	time varying pressure enforced in time-reversed order as a Dirichlet boundary condition over `sensor.mask`
`sensor.frequency_response`	two element array specifying the center frequency and percentage bandwidth of a frequency domain Gaussian filter applied to the `sensor_data`
`sensor.directivity_angle`[1]	matrix of directivity angles (direction of maximum response) for each sensor element defined in `sensor.mask`. The angles are specified in radians where `0` corresponds to maximum sensitivity in x direction and `pi/2` or `-pi/2` to maximum sensitivity in y direction
`sensor.directivity_size`[1]	equivalent element size [m] (the larger the element size the more directional the response)

[1] Only supported in 2D

the complete computational grid regardless of the sensor points defined by `sensor.mask` and are indexed as `(nx, 1)` in 1D, `(nx, ny)` in 2D, and `(nx, ny, nz)` in 3D.

When defining and using the simulation inputs and outputs, it's important to remember the effect of the staggered grid scheme used by k-Wave (see Sec. 2.4 and Table 2.1 for reference). In particular, the outputs for the particle velocity are both spatially and temporally staggered compared to the output for the pressure. For example, `sensor_data.ux` is obtained at grid points staggered in the x-direction by `+kgrid.dx/2` and in the temporal direction by `-kgrid.dt/2`. If `sensor.record` includes `'u_non_staggered'` or either of the intensity parameters `'I'` or `'I_avg'`, values for the particle velocity at the unstaggered grid points are automatically calculated within the simulation functions using Fourier interpolation.

The sensor points defined by `sensor.mask` do not modify the wave field in any way. Rather, they act as transparent observers recording the numerical values of the pressure and particle velocity within the domain. The response of any given sensor point is also omni-directional, meaning it is equally sensitive to waves from any direction. For simulations in 2D using a binary sensor mask, it is possible to set the directivity of the individual sensor points using `sensor.directivity_angle` and `sensor.directivity_size` [57]. The directivity angle corresponds to the direction of maximum response. It is given as a 2D matrix the same size as the computational grid, with a value for each sensor point specified in `sensor.mask`. The angles are specified in radians where `0` corresponds to maximum sensitivity in x-direction and `pi/2` or `-pi/2` to maximum sensitivity in y-direction. The directivity size sets the equivalent element size in metres. The larger the element size the more directional the response.

3.6 Optional Input Parameters

In addition to the properties defined by the input structures `kgrid`, `medium`, `source`, and `sensor`, a number of other parameters controlling the default behaviour of k-Wave can be set using optional input parameters. These are defined as `'string'` / `value` pairs. The `'string'` identifies the optional input parameter that is being modified, and the `value` is the user setting for this parameter. Several examples are given below.

```
% optional input to change the default plot scale
kspaceFirstOrder2D(kgrid, medium, source, sensor, 'PlotScale', [-10, 10]);

% optional input to change the PML thickness for a 2D simulation
kspaceFirstOrder2D(kgrid, medium, source, sensor, 'PMLSize', [15, 10]);

% optional inputs to hide the PML from display and save a movie
input_args = {'PlotPML', false, 'RecordMovie', true}
kspaceFirstOrder2D(kgrid, medium, source, sensor, input_args{:});
```

There are a large number of parameters that can be tweaked if desired. A complete list is given in Table A.1 in Appendix A.

3.7 Using a Diagnostic Ultrasound Transducer as a Source or Sensor

In principle, simulations using diagnostic ultrasound transducers can be performed by creating the appropriate source and sensor inputs as described in the previous sections. However, assigning the grid points that belong to each physical transducer element, and then assigning the correctly delayed input signals to each point of each element can soon become an indexing nightmare. For this purpose, k-Wave provides a special transducer class which takes care of creating the masks and assigning the input and output signals. Objects of this class can be used to replace the `source` and/or `sensor` inputs of `kspaceFirstOrder3D`.

The transducer is created by calling `makeTransducer` which returns an object of the `kWaveTransducer` class. This function is called with two inputs:

```
% create an object of the kWaveTransducer class using makeTransducer
transducer = makeTransducer(kgrid, input_settings);
```

The first input `kgrid` is an object of the `kWaveGrid` class and describes the properties of the computational grid as discussed in Sec. 3.2. The second input is a structure with user defined input properties appended as fields in the form `structure.field`. The input properties can be broken into two groups and are listed in Table 3.5. Properties belonging to the first group are fixed when the transducer is created (for example the position of the transducer and the number of transducer elements). These properties can be queried, but not modified after the object is returned. Properties belonging to the second group can be modified at any time (for example the steering angle or focus distance). Fields that are not defined by the user are given their default values.

An example of defining a linear diagnostic ultrasound transducer using `makeTransducer` is given on page 42. First, the physical properties of the transducer are defined, including the number of elements and their size. The element sizes are given in units of grid points. This means the physical size of the transducer is dependent on the values of `kgrid.dx`, `kgrid.dy`, and `kgrid.dz`. When the transducer is created, the set of grid points that belong to each each physical transducer element is stored, and is recalled by `kspaceFirstOrder3D` as necessary.

A schematic illustrating the physical properties of the transducer is shown in Fig. 3.2(a). The transducer is always orientated within the computational grid such that the front face is pointing in the positive x-direction. The position of the transducer within the grid is set using the `position` field. This defines the position of the nearest grid point belonging to the transducer relative to the grid origin. For example, if `position` is set to `[1, 1, 1]`, the corner of the transducer will be positioned flush with the grid origin.

The settings for sound speed, focus distance, and steering angle are used to calculate the beamforming delays based on geometric beamforming expressions. For a steered beam, the delays are given by [58]

$$d_n = \text{round}\left(\frac{i_n \times \text{pitch} \times \sin(\pi\theta/180)}{c_0 \times dt}\right) \quad , \tag{3.1}$$

```
% define the physical properties of the transducer
tr.number_elements = 128;     % total number of transducer elements
tr.element_width = 1;         % width of each element [grid points]
tr.element_length = 12;       % length of each element [grid points]
tr.element_spacing = 0;       % spacing between the elements [grid points]
tr.radius = Inf;              % radius of curvature of the transducer [m]

% define the position of the transducer [grid points]
tr.position = [1, 20, 20];

% define the properties used to derive the beamforming delays
tr.sound_speed = 1540;        % sound speed [m/s]
tr.focus_distance = 20e-3;    % focus distance [m]
tr.steering_angle = 10;       % steering angle [degrees]

% define the apodization
tr.transmit_apodization = 'Rectangular';
tr.receive_apodization = 'Hanning';

% define the transducer elements that are currently active
tr.active_elements = zeros(tr.number_elements, 1);
tr.active_elements(1:32) = 1;

% define the input signal used to drive the transducer
tr.input_signal = input_signal;

% create the transducer using the defined settings
transducer = makeTransducer(kgrid, tr);

% display the transducer using a 3D voxel plot
transducer.plot;

% print a list of transducer properties to the command line
transducer.properties;

.
.
.

% run a simulation using the same transducer as both source and sensor
sensor_data = kspaceFirstOrder3D(kgrid, medium, transducer, transducer);

% form the recorded sensor data in a single scan line based on the current
% beamforming and apodization settings
scan_line = transducer.scan_line(sensor_data);
```

where d_n is the delay in time points for the nth transducer element, i_n is the element index, pitch is the element pitch in metres, and θ is the steering angle in degrees. For a beam that is both focussed and steered, the delays are instead given by [58]

$$d_n = \text{round}\left(\frac{F}{c_0 \times dt}\left(1 - \sqrt{1 + \left(\frac{i_n \times \text{pitch}}{F}\right)^2 - 2\sin(\pi\theta/180)\left(\frac{i_n \times \text{pitch}}{F}\right)}\right)\right) . \tag{3.2}$$

The steering angle is defined relative to the transducer axis as shown in Fig. 3.2(b).

The setting for elevation focus distance is used to mimic the focussing behaviour of physical transducers in which an acoustic lens is used to focus the beam in the out-of-plane (x-z) or elevation direction. Within k-Wave, this behaviour is modelled by using an additional set of beamforming delays across the grid points in the z-direction within each element. The current beamforming delays can be queried after the transducer is created using `transducer.beamforming_delays` and `transducer.elevation_beamforming_delays`.

If the transducer is used as an ultrasound source, the input signal used to drive the transducer elements must be defined before the transducer is created. A single input signal is used, with the beamforming delays calculated automatically based on the user settings for `transducer.focus_distance`, `transducer.elevation_focus_distance`, and `transducer.steering_angle`. The input signal can be any 1D vector and is injected as a time varying velocity (or force) source in the x-direction (this is equivalent to defining `source.ux` if the input was being assigned manually). Consequently, the input signal must be scaled to be in units of velocity rather than pressure.

Within `kspaceFirstOrder3D`, the beamforming delays are used in reverse order as an indexing variable. The value of the index at each grid point is used to select which element of the input signal should be applied, with the index incremented after each time step. This is illustrated in Fig. 3.2(c). The calculated beamforming delays are reversed and then offset such that the indices are equal to or greater than zero. If `steering_angle_max` is not set by the user, this offset is calculated automatically. If a value for `steering_angle_max` is defined, a constant offset equal to the minimum beamforming delay at the maximum steering angle is used, regardless of the current steering angle. This behaviour is useful if forming an ultrasound image by steering the beam through a range of angles, as the index of t_0 relative to the central transducer element will stay constant. The value of the offset can be queried using `transducer.beamforming_delays_offset` (this returns `'auto'` if `steering_angle_max` is not set). To account for the range of indices produced by the beamforming delays, the input signal defined by the user is also appended and prepended with zeros. The number of zeros is calculated automatically if `steering_angle_max` is not defined, otherwise, the number of zeros required at the maximum steering angle is used. If `transducer.input_signal` is queried after the transducer is created, the input signal with appended zeros is returned.

The settings for `transmit_apodization` and `receive_apodization` control the relative weights assigned to the signal driving each of the active transducer elements. This is defined as a string corresponding to any valid window type supported by `getWin`, for example, `'Hanning'` or `'Gaussian'`. It can also be manually defined as a 1D vector of relative weights applied to the active transducer elements, or not defined (this defaults

to `'Rectangular'`). The transmit apodization is applied when the transducer is used as a source, while the receive apodization is applied when the transducer is used as a sensor.

While many diagnostic ultrasound transducers have 128 or 256 physical transducer elements, normally only a small subset of these are used to transmit and receive ultrasound signals at any particular time (sector transducers are an exception). The transducer elements that are currently active can be defined using the `active_elements` field, which is assigned as a 1D binary matrix. In the example on page 42, the first 32 elements of a 128 element transducer are set to be active.

Objects of the `kWaveTransducer` class created using `makeTransducer` can also be used to replace the `sensor` input. In this case, the signals recorded at the grid points belonging to each physical transducer element are automatically averaged. For example, if the transducer has 32 active elements, 32 signals will be returned, regardless of the size of each element in grid points. These signals are indexed as `sensor_data(element_index, time_index)`. The way in which the signals across each element are calculated depends on the setting for `transducer.elevation_focus_distance`. If this is set to `Inf`, the signals across the grid points within each sensor element are averaged at each time step, and only the average is stored. If a finite elevation focus distance is defined, a buffer the length of the longest beamforming delay is filled using a FIFO queue (first-in, first-out). The elevation beamforming is then computed on the fly once the buffer is filled. In both cases, computing the average at each time step significantly reduces the memory requirements compared to storing the complete time history at every grid point within the transducer.

After the sensor data is returned, the signals recorded by each transducer element can be formed into a scan line by using the functionality of the `kWaveTransducer` class. The `scan_line` method takes the recorded sensor data and forms it into a scan line based on the current beamforming and receive apodization settings (see example on page 42). A summary of the additional properties and methods that can be accessed by objects of the `kWaveTransducer` class is given in Table 3.6.

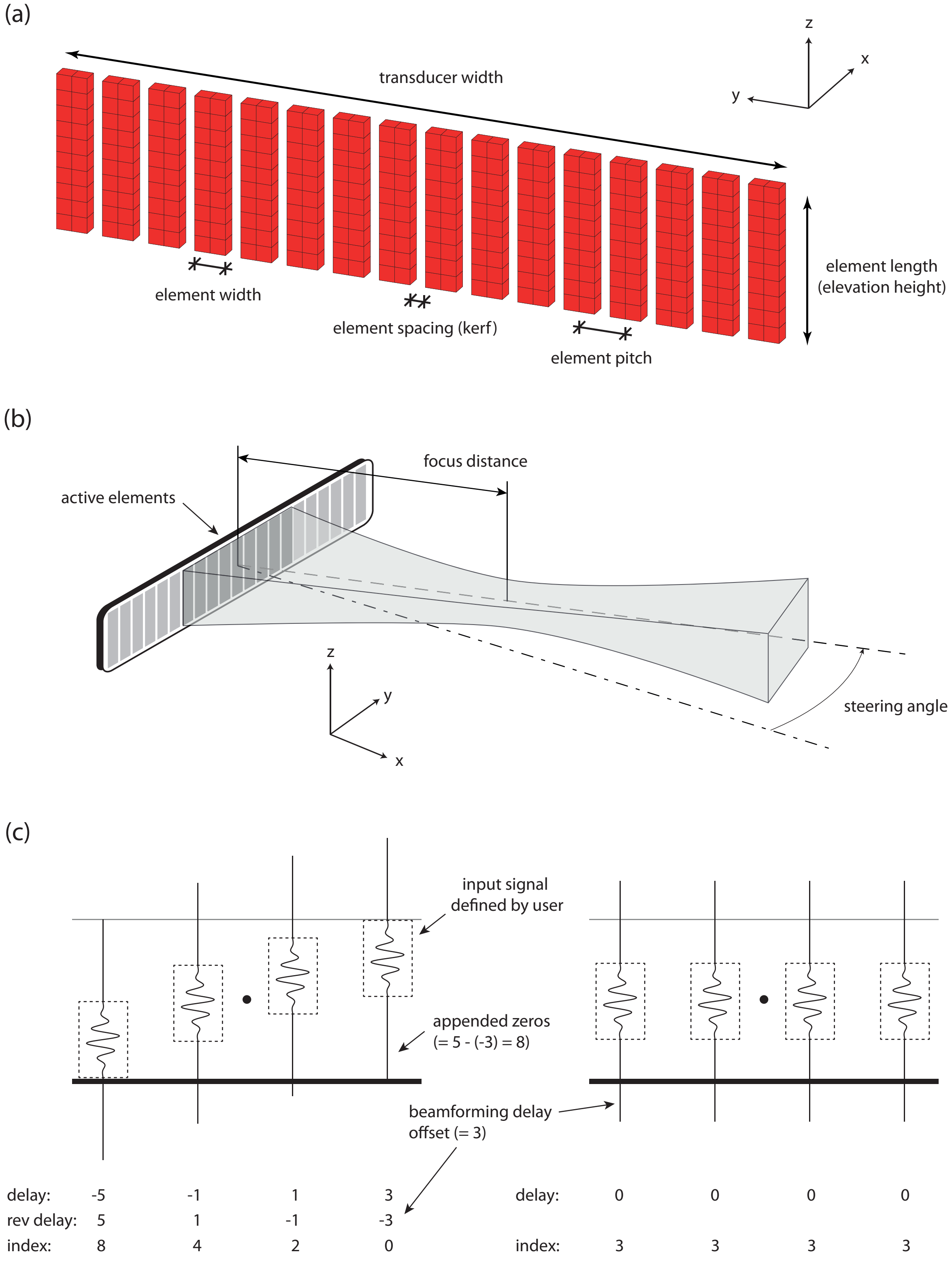

Figure 3.2: Schematic of the (a) physical and (b) dynamic properties used to define objects of the `kWaveTransducer` class. (c) Illustration of how the beamforming delays are used as an index to select which value from the input signal is used at each transducer element.

Table 3.5: Input fields used to create objects of the `kWaveTransducer` class using `makeTransducer`. After the transducer has been created, the first group of properties cannot be modified. The second group of properties can be defined or modified at any time. Properties not defined by the user are given their default values.

Fieldname	Default	Description
`tr.number_elements`	`128`	total number of transducer elements
`tr.element_width`	`1`	width of each element [grid points]
`tr.element_length`	`20`	length of each element [grid points]
`tr.element_spacing`	`0`	spacing (kerf width) between elements [grid points]
`tr.position`	`[1, 1, 1]`	position of the top corner of the transducer within the grid [grid points]
`tr.radius`	`Inf`	radius of curvature of the transducer face—currently only `inf` is supported [m]
`tr.input_signal`	`[]`	signal used to drive the ultrasound transducer if used as a source
`tr.active_elements`	*all elements*	binary vector indicating which transducer elements are currently active
`tr.focus_distance`	`Inf`	focus distance used to calculate beamforming delays [m]
`tr.steering_angle`	`0`	steering angle used to calculate the beamforming delays [deg]
`tr.steering_angle_max`	`'auto'`	used to set a fixed offset for the beamforming delays [deg]
`tr.elevation_focus_distance`	`Inf`	fixed elevation focus distance [m]
`tr.transmit_apodization`	`'Rectangular'`	apodization used on transmit
`tr.receive_apodization`	`'Rectangular'`	apodization used on receive
`tr.sound_speed`	`1540`	sound speed used to calculate beam forming delays [m/s]
`tr.record_start_index`	`1`	time index at which the transducer should start recording if used as a sensor

Table 3.6: Additional properties and methods for objects of the `kWaveTransducer` class.

Property / Method	Description
`tr.transducer_width`	total width of the transducer in grid points
`tr.number_active_elements`	current number of active transducer elements
`tr.mask`	binary mask of the active transducer elements
`tr.active_elements_mask`	binary mask of the active transducer elements (identical to `tr.mask`)
`tr.indexed_active_elements_mask`	indexed mask of the active transducer elements, where the index indicates the transducer element that each grid point belongs to
`tr.all_elements_mask`	binary mask of all the transducer elements (both active and inactive)
`tr.indexed_elements_mask`	indexed mask of all the transducer elements (both active and inactive)
`tr.beamforming_delays`	vector of the azimuthal beamforming delays (in units of time samples) for each active element based on the focus and steering angle settings
`tr.elevation_beamforming_delays`	vector of length `tr.element_length` containing the beamforming delays in the elevation direction (in units of time samples) based on the `tr.elevation_focus_distance`
`tr.beamforming_delays_offset`	offset used to make the the beamforming delays positive based on `tr.steering_angle_max`
`tr.appended_zeros`	number of zeros added to the start and end of the input signal based on `tr.steering_angle_max`
`tr.scan_line`	method to create a scan line based on a user input for `sensor_data` and the current apodization and beamforming settings
`tr.plot`	method to produce a 3D plot of the active transducer elements using `voxelPlot`
`tr.properties`	method to print a list of the current transducer properties to the command line

3.8 Improving Performance using the 'DataCast' Option

By default, numbers in MATLAB are stored in double precision. This means that 8 bytes (or 64 bits) of memory are used to store each number. Accounting for the sign and the exponent, 53 of the 64 bits are used to store the actual digits, which corresponds to roughly 16 decimal places of precision (the MATLAB function `eps` gives the exact value of the smallest possible difference between two double precision numbers). However, in almost all cases, k-Wave does not require this level of precision. In particular, the performance of the PML generally limits the accuracy to around 4 or 5 decimal places at best. For example, using a PML thickness of 20 grid points gives a normal incidence transmission coefficient of -100 dB (see discussion in Sec. 2.6). This corresponds to a reduction in signal level of 1×10^{-5}, which is significantly less than double precision. In many cases, there will also be uncertainties in the definition of the material properties, source inputs, etc.

It is possible to reduce the memory consumption and improve the speed of k-Wave by performing simulations in single precision instead of double precision. This means only 4 bytes (or 32 bits) of memory are used to store each number, giving a precision of roughly 8 decimal places. The data type used within the time loop can be set using the optional input parameter `'DataCast'`. By default, this is set to `'off'` (which is equivalent to `'double'`). To run simulations in single precision, this should be set to `'single'`. For example:

```
% perform a 3D simulation in single precision
kspaceFirstOrder3D(kgrid, medium, source, sensor, 'DataCast', 'single');
```

In this case, the user inputs for the medium and source parameters are cast to single precision before the simulation begins. The output variables are also allocated and returned in single precision. In addition to the memory saving, this has a direct impact on the total compute time, as all the operations within the time loop (including the FFTs) are performed using single precision arithmetic. A plot of the compute times per time step for a range of grid sizes in 2D and 3D are shown in Fig. 3.3. Simulations performed using single precision are roughly 1.5× to 1.6× faster than those performed in double precision.

The compute times can be further reduced by using other data types, in particular those which force program execution on a graphics processing unit (GPU). MATLAB 2012a and later allows computations on NVIDIA CUDA-enabled GPUs via the Parallel Computing Toolbox. This contains overloaded MATLAB functions (such as `fft`) which allow code to be executed on the GPU simply by casting the variables to the required GPU data type. The syntax for running a k-Wave simulation in single precision on the GPU is illustrated below.

```
% run simulation on the GPU using the MATLAB parallel computing toolbox
kspaceFirstOrder3D(kgrid, medium, source, sensor, ...
  'DataCast', 'gpuArray-single');
```

A plot of the corresponding compute times per time step for a range of grid sizes is shown in Fig. 3.3. For larger grid sizes, the use of a GPU can give an order of magnitude speed-

up[1] compared to using double precision on a 8-core CPU. Note, that the output variables are also returned in the format specified by `'DataCast'`. For GPU simulations, this means the output results will still be stored on the GPU. These can be manually recast as CPU variables as required, or automatically returned in double precision by setting the optional input parameter `'DataRecast'` to `true`.

The primary limitation of running simulations on a GPU is the amount of available memory. Current high-end NVIDIA cards have 12 GB of memory (slightly less if ECC is enabled), which is sufficient to run simulations in single precision with around 67×10^6 grid points ($512 \times 512 \times 256$). For larger simulations, the optimised C++ code (discussed in Chapter 4) can be used to reduce compute times. In 3D, it is also possible to run simulations on a GPU using a native CUDA code that has been heavily optimised (see Chapter 4).

[1] Don't be fooled by the gigantic GPU speed-ups sometimes reported in the literature—in many cases, this just means the CPU code was single threaded or not very well optimised! [59, 60]

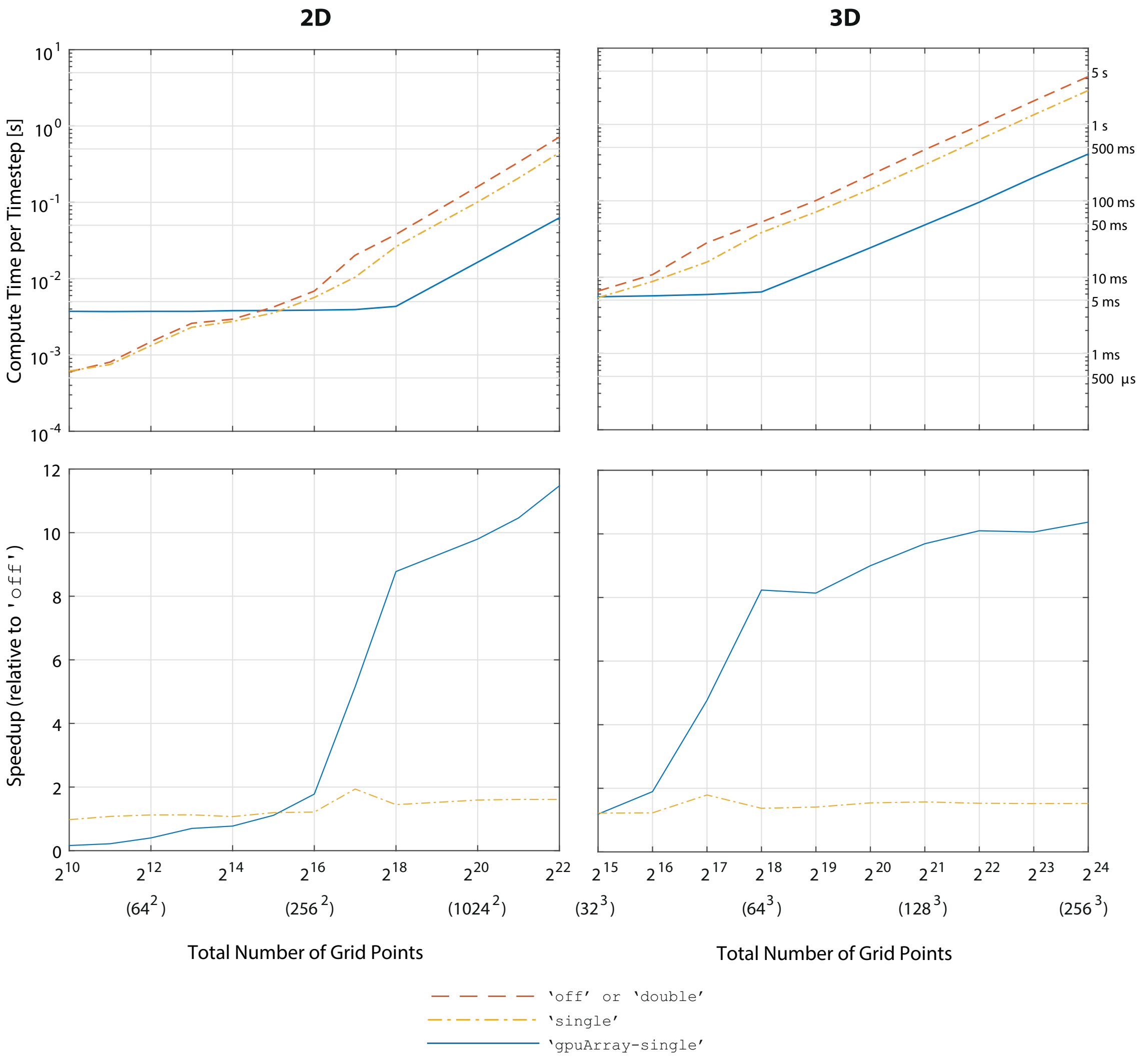

Figure 3.3: Compute times per time-step for different `'DataCast'` options. The tests were performed using `benchmark` on a desktop computer with an eight-core Intel Xeon E5-1660 v3 @ 3.00 GHz processor, 64 GB of 2133MHz DDR4 RAM, and an NVIDIA Tesla C2070 GPU with 448 CUDA cores and 6 GB of GDDR5 RAM. The computer was installed with 64-bit Windows 10, NVIDIA DRIVER 354.42, MATLAB 2015a, Parallel Computing Toolbox 2015a, and k-Wave 1.1. The speedups are shown relative to `'DataCast', 'off'` running on the CPU.

Chapter 4

Using optimised CPU and GPU Codes

4.1 Overview

MATLAB is a very powerful environment for scientific computing. It interfaces with a number of highly-optimised matrix and maths libraries, and can be programmed using relatively simple syntax. User written MATLAB code is also highly portable across operating systems and computer platforms. The portability comes from MATLAB being an interpreted language. This means the code is analysed and executed line by line as the program runs, without needing to be compiled into machine code in advance. In most situations, this means the k-Wave Toolbox works straight out of the (virtual) box on any computer installed with MATLAB.

The downside of MATLAB being an interpreted language is that it can be difficult to optimise user code. For example, within the main time loop of the simulation functions, the code consists primarily of FFTs and element-wise matrix operations like multiplication and addition. Executing these tasks involves a large number of memory operations for relatively few compute operations. For example, to multiply two matrices together element by element, the individual matrix elements are transferred from main memory to cache in blocks, multiplied together using the CPU, and then the results transferred back to main memory (see Fig. 4.1). If the matrix elements were already stored in cache, the multiplication would be an order of magnitude faster. However, because the lines of code in MATLAB are executed independently, there is no way in MATLAB to fuse together multiple operations to maximise the temporal and spatial locality of the data.

For simulations using small grid sizes, the compute times are short enough that the drawbacks of MATLAB being an interpreted language are not a concern. However, for simulations using large grid sizes (for example, 1024 × 1024 × 1024), or large batches of moderate grid sizes (for example, 256 × 256 × 256), the compute times in MATLAB can stretch into tens of hours. To reduce these compute times, k-Wave also includes two highly optimised versions of `kspaceFirstOrder3D`. The first, called `kspaceFirstOrder3D-OMP` is written in C++ for shared memory computer architectures, including machines with mul-

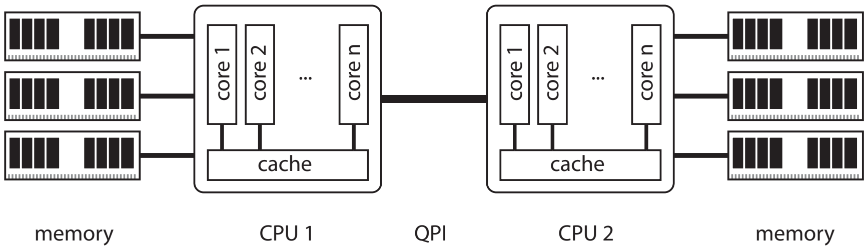

Figure 4.1: Schematic of a two-socket computer based on the Non-Uniform Memory Access (NUMA) architecture. Non-local memory attached to a different CPU can be accessed via the Quick Path Interconnect (QPI). However, this is considerably slower than accessing local memory. The CPU code implements policies to bind threads to cores and to allocate memory to nearby memory locality domains wherever possible to maximum performance.

tiple CPUs based on a non-uniform memory access (NUMA) design. The second, called `kspaceFirstOrder3D-CUDA`, is written using CUDA for NVIDIA GPUs.

The CPU code is written in C++11 and is parallelised using OpenMP with the use of either Streaming SIMD Extensions (SSE) or Advanced Vector Extensions (AVX/AVX2), depending on the processor architecture [2]. Performance is optimised by fusing multiple operations together to maximise temporal and spatial data locality. For NUMA (multiple socket) architectures, it is necessary to bind threads to cores and to allocate memory to nearby memory locality domains wherever possible (see Fig. 4.1). Thread binding can be enabled by setting the environmental variable `OMP_PROC_BIND=true`. The thread placement can be adjusted by setting the environment variable `OMP_PLACES=threads` for systems with Intel HyperThreading, or `OMP_PLACES=cores` for systems without Intel HyperThreading. The GPU code is written in C++11 and CUDA for NVIDIA graphics cards (GPU cards by AMD are not supported). Only a single GPU is supported.

Compiled binaries of the CPU and GPU codes for x86-64 architectures are available from `http://www.k-wave.org/download.php`. Both 64-bit Linux (Ubuntu / Debian) and 64-bit Windows versions are provided. The CPU code is compiled using the Intel C++ 2016 compiler, the Intel Math Kernel Library (MKL) (for the FFT), and the HDF5 library (for handling the input and output files). The GPU code is compiled using either the GNU 4.8.5 compiler (Linux) or the MS Visual Studio 2013 compiler (Windows), CUDA 7.5, and the HDF5 library. All operations are performed in single precision. Although we will happily provide support for Windows users, our experience is largely with Linux-based operating systems. Questions should be directed to `http://www.k-wave.org/forum`.

4.2 Running Simulations using the Optimised Codes

The optimised CPU and GPU codes require a single input file saved in HDF5 format (a detailed discussion of the input and output file format is given in Sec. 4.6). This file defines the properties of the grid, medium, source, and sensor in the same way as described in Chapter 3. Although the optimised codes are written to be run independently of MATLAB, for most users it is easiest to use the MATLAB function `kspaceFirstOrder3D`

to create the input matrices and save them to disk in the required format (this requires MATLAB 2011a or later). The HDF5 input file is automatically generated by adding the flag `'SaveToDisk'` and a filename (or pathname and filename) to the optional input arguments as shown below. When this flag is given, the MATLAB code runs the pre-processing steps, saves the input parameters to disk, and then aborts without running the actual simulation.

```
% save input files to disk
filename = 'kwave_input_data.h5';
kspaceFirstOrder3D(kgrid, medium, source, sensor, 'SaveToDisk', filename);
```

After saving the input data, the optimised codes can be called from a terminal window (Linux) or the command prompt (Windows). The codes require two mandatory parameters, in addition to a number of optional parameters and flags. A full list is given in Table 4.1.

The mandatory parameters `-i` and `-o` specify the input and output file. The file names respect the path conventions for the particular operating system. If any of the files are not specified, cannot be found, created, or opened (etc), the code is terminated. Similarly, any misdefined input parameters, corrupt or incomplete simulation files, or runtime errors such as out-of-memory problems, will lead to an exception followed by an error message and the termination of the code.

The `-h` and `--help` parameters print information regarding the various code inputs and parameters, while the `--version` parameter reports detailed information about the code useful for debugging and bug reports. This includes the internal code version, the build date and time, the git hash (allowing us to track the version of the source code), the operating system, the compiler name and version, and the instruction set used.

The `-r` parameter specifies how often information about the simulation progress is printed out to the command line. By default, the CUDA/C++ codes print out the progress of the simulation, the elapsed time, and the estimated time of completion in intervals corresponding to 5% of the total number of times steps.

The `--verbose` parameter allows the user to select between three levels of verbosity for the output printed to the command line. For routine simulations, a verbose level of 0 (the default) is usually sufficient. For more information about the simulation, input parameters, code version, GPU used, file paths, or debugging running scripts, verbose levels 1 and 2 may also be useful.

The `-t` parameter sets the number of threads used, which defaults to the system maximum. On CPUs with Intel Hyper-Threading (HT), performance will generally be better if HT is disabled in the BIOS settings. If HT is switched on, the default will be to create one thread per virtual core (usually, every physical core consists of two virtual cores). In this case, some hardware resources are shared between the two threads which may reduce performance. Therefore, if HT is on, try specifying the number of threads manually for best performance (e.g., 4 for a quad core Intel Core i7). We recommend experimenting with this parameter to find the best configuration for a given simulation, in addition to trying different OpenMP placement strategies using the environmental variables `OMP_PLACES` and `OMP_PROC_BIND`. Note, if there are other tasks being executed on the system, it might be

Table 4.1: List of input and output parameters for the optimised CPU and GPU codes.

Mandatory parameters	
-i <file_name>	name of HDF5 input file
-o <file_name>	name of HDF5 output file
Optional parameters	
-h, --help	print help
--version	print version and build info
-r <interval_in_\%>	progress print interval (default = 5%)
--verbose <level>	level of verbosity $< 0, 2 >$ (default = 0)
-t <num_threads>	number of CPU threads (default = MAX)
-c <compression_level>	output file compression level $< 0, 9 >$ (default = 0)
-s <time_step>	time step when data collection begins (default = 1)
-g <device_number>	GPU device to run on (default is first free)
--benchmark <time_steps>	run only a specified number of time steps
--checkpoint_interval <sec>	checkpoint after a given number of seconds
--checkpoint_file <file_name>	name of HDF5 checkpoint file
Output flags	
-p, --p_raw	store time varying acoustic pressure (default)
--p_rms	store rms of p
--p_max	store max of p
--p_min	store min of p
--p_max_all	store max of p (whole domain)
--p_min_all	store min of p (whole domain)
--p_final	store final pressure field
-u, --u_raw	store time varying particle velocity (ux, uy, uz)
--u_non_staggered_raw	store ux, uy, uz on non-staggered spatial grid
--u_rms	store rms of ux, uy, uz
--u_max	store max of ux, uy, uz
--u_min	store min of ux, uy, uz
--u_max_all	store max of ux, uy, uz (whole domain)
--u_min_all	store min of ux, uy, uz (whole domain)
--u_final	store final particle velocity field
--copy_sensor_mask	copy sensor mask to output file

useful to further limit the number of threads. This is because the CPU code does not implement any kind of dynamic load balancing, thus sharing even a single core with other compute intensive tasks will slow the simulation down significantly. For the GPU code, it is usually better to set the number of threads to 1 since only the pre-processing and data collection are performed on the CPU. On Multi-GPU systems, this also leads to better CPU sharing among several instances of the GPU code.

The `-c` parameter specifies the compression level used by the ZIP library to reduce the size of the HDF5 output file. The actual compression rate is highly dependent on the shape of the sensor mask and the range of stored quantities. In general, the output data is very hard to compress, and using higher compression levels can greatly increase the time to save data while not having a large impact on the final file size. By default, compression is disabled.

The `-g` parameter (`kspaceFirstOrder3D-CUDA` only) allows the user to explicitly select a GPU to use on multi-GPU systems. A list of CUDA capable GPUs can be displayed using the system command `nvidia-smi` (on Windows this is in the folder `C:\Program Files\NVIDIA Corporation\NVSMI\` and can be run using the command prompt). If the `-g` parameter is not specified, the code uses the first free GPU. However, if the GPUs are set in `DEFAULT` compute mode, this means the first CUDA device is always selected, even if another simulation is already running on this GPU. In order to use automatic round-robin GPU selection (e.g., to automatically execute multiple instances of the code on different GPUs), the GPUs should be set into `EXCLUSIVE_PROCESS` compute mode. This can be set using `nvidia-smi -c 3`. On clusters with a PBS scheduler, this is usually done automatically, so there is generally no need to specify the GPU or set the GPU compute mode.

The `--benchmark` parameter enables the total length of simulation (i.e., the number of time steps) to be overwritten by setting a new number of time steps to simulate. This is particularly useful for performance evaluation and benchmarking. As the code performance is relatively stable, 50-100 time steps is usually enough to predict the simulation duration. This parameter can also be used to quickly find the ideal number of CPU threads to use, the best domain size to cover the simulation space, etc.

For jobs that are expected to run for a very long time, it may be useful to checkpoint and restart the execution. One motivation is the wall clock limit per task on clusters where jobs must fit within a given time span (e.g., 24 hours). The second motivation is a level of fault-tolerance where the state of the simulation can be backed up after a predefined period. To enable checkpoint-restart, the user must specify the period in seconds after which the simulation will be interrupted for checkpointing, and a HDF5 file in which the state of the simulation is stored. These are specified using the parameters `--checkpoint_interval` and `--checkpoint_file`, respectively. When running on a cluster, it is important to allocate enough time for the checkpoint procedure. This can take a non-negligible amount of time (7 matrices have to be stored in the checkpoint file and all aggregated quantities flushed into the output file). Please note, the checkpoint file name and path is not checked at the beginning of the simulation, but at the time the code starts checkpointing. Thus, it is important to make sure the file path is correctly specified (otherwise the simulation will crash after the specified checkpoint interval).

When controlling a multi-leg simulation using a loop in a bash script, the parameters of the code remain the same in all simulation legs. The first simulation leg creates a checkpoint file while the last one deletes it. If the checkpoint file is not found, the simulation starts from the beginning. To find out how many steps have been completed, open the output file and read the variable `t_index` and compare it with `Nt` (e.g., using the `h5dump` command).

The remaining flags specify the output quantities to be recorded during the simulation and stored on disk analogous to the `sensor.record` input discussed in Sec. 3.5. If the `-p` or `--p_raw` flags are set (these are equivalent), a time series of the acoustic pressure at the grid points specified by the sensor mask is recorded. If the `--p_rms`, `--p_max` and `--p_min` flags are set, the root mean square, maximum, and minimum values of the pressure at the grid points specified by the sensor mask are recorded, respectively. If the `--p_final` flag is set, the values for the entire acoustic pressure field after the final time step of the simulation is stored (this will always include the PML, regardless of the setting for `'PMLInside'`). The flags `--p_max_all` and `--p_min_all` allow the maximum and minimum values over the entire acoustic pressure field to be recorded, regardless on the shape of the sensor mask. Flags to record the acoustic particle velocity are defined in an analogous fashion. The particle velocity on the non-staggered spatial grid (see Sec. 3.5) can be recorded using the `--u_non_staggered_raw` flag. Note, since the shift operation requires additional FFTs, the impact on the simulation time may be significant.

Any combination of `-p` and `-u` flags is admissible. If no output flag is set, a time-series for the acoustic pressure is recorded. If it is not necessary to collect the output quantities over the entire simulation, the starting time step when the collection begins can be specified using the `-s` parameter. Note, the index for the first time step is 1 (this follows the MATLAB indexing convention).

The `--copy_sensor_mask` flag will copy the sensor from the input file to the output one at the end of the simulation. This helps in post-processing and visualisation of the outputs.

Note, not all simulation options are currently supported by the CPU/GPU code. The sensor mask must be given as either a binary matrix or a cuboid-corner sensor mask (Cartesian sensor masks are not supported). All display parameters are ignored (the C++/CUDA codes do not have a graphical output).

4.3 Reloading the Output Data into MATLAB

After the C++/CUDA code has executed, the output files can be reloaded into MATLAB using the syntax `h5read(filename, datasetname)`. The variable fields stored in the HDF5 output file have the identical names to the MATLAB code discussed in Sec. 3.5. For example, for a simulation run with the `-p` and `-u` flags (equivalent to `sensor.record = {'p', 'u'}`) and a binary sensor mask, the output fields can be loaded into MATLAB as shown below.

```
% load output data from a CPU/GPU simulation
sensor_data.p  = h5read('output_filename.h5', '/p');
```

```
sensor_data.ux = h5read('output_filename.h5', '/ux');
sensor_data.uy = h5read('output_filename.h5', '/uy');
sensor_data.uz = h5read('output_filename.h5', '/uz');
```

For simulations using a sensor mask defined using opposing corners of a cuboid (see Sec. 3.5), the region index must also be specified. For example, for a sensor mask with two sensor regions specified by cuboid corners, the output can be loaded into MATLAB as shown below.

```
% load output data from first cuboid
sensor_data(1).p = h5read('output_filename.h5', '/p/1');

% load output data from second cuboid
sensor_data(2).p = h5read('output_filename.h5', '/p/2');
```

4.4 Running the Code using a Bash Script

For Linux users, it may be useful to use a bash script to run simulations, particularly when many simulations must be performed, or when the code is executed on a remote machine with a job submission system. A simple example of running a k-Wave simulation using a bash script is given below (save the script as `filename.sh`).

```
#!/bin/bash

# Use the k-Wave MATLAB toolbox to save the input data to disk
matlab -nojvm -r "addpath('k-Wave'); my_script; exit;"

# Bind threads to CPU cores and forbid thread migration
export OMP_PLACES=cores
export OMP_PROC_BIND=true

# Run simulation using the CPU code
./kspaceFirstOrder3D-OMP -i input_data.h5 -o output_data.h5

# Exit the script
exit
```

The MATLAB startup option `-nojvm` switches off the graphical display, and `-r` executes the statements in batch mode. The files must be within the same directory as the bash script, or `cd` can be used. The `exit` command is always required as the last statement otherwise the call to `matlab` will never return.

4.5 Running the Code from MATLAB

It is also possible to run the optimised CPU/GPU code directly from MATLAB (rather than from a terminal or command window). To run the code blindly, calls to

kspaceFirstOrder3D can be directly substituted with calls to kspaceFirstOrder3DC (CPU code) or kspaceFirstOrder3DG (GPU code) without any other changes. This automatically adds the 'SaveToDisk' flag, calls kspaceFirstOrder3D to create the input variables, calls the CPU/GPU code using the system command, reloads the output variables from disk using h5read, then deletes the input and output files. This is useful when running MATLAB interactively. The disadvantage of running the CPU/GPU code from within MATLAB is the additional memory footprint of having many variables allocated in main memory twice, as well as the overhead of running MATLAB. To test whether the CPU/GPU codes are working correctly, open the "Diagnostic Ultrasound Simultion, Simulating Ultrasound Beam Patterns Example", replace kspaceFirstOrder3D with kspaceFirstOrder3DC or kspaceFirstOrder3DG, then run the simulation and verify the outputs are the same.

4.6 Format of the HDF5 Input and Output files

The CPU/GPU codes have been designed as standalone applications not dependent on MATLAB libraries or a MEX interface. This is of particular importance when using servers and supercomputers without MATLAB support. For this reason, simulation data must be transferred between the optimised CPU/GPU code and MATLAB using external input and output files. These files are stored using the Hierarchical Data Format HDF5 (http://www.hdfgroup.org/HDF5/). This is a data model, library, and file format for storing and managing data. It supports a variety of datatypes, and is designed for flexible and efficient I/O and for high volume and complex data. The HDF5 technology suite includes tools and applications for managing, manipulating, viewing, and analysing data in the HDF5 format.

Each HDF5 file is a container for storing a variety of scientific data and is composed of two primary types of objects: groups and datasets. A HDF5 group is a structure containing zero or more HDF5 objects, together with supporting metadata, and can be thought of as a disk folder. A HDF5 dataset is a multidimensional array of data elements, together with supporting metadata, and can be thought of as a disk file. Any HDF5 group or dataset may also have an associated attribute list. A HDF5 attribute is a user-defined HDF5 structure that provides extra information about a HDF5 object. More information can be obtained from the HDF5 documentation (http://www.hdfgroup.org/HDF5/doc/index.html).

Note, the CPU and GPU code for k-Wave V1.0 and V1.1 use slightly formats for the HDF5 input and output files. The code for k-Wave V1.1 will run input files for both versions (it is backwards compatible), however, when working with an input file from V1.0, some features are not supported, namely the cuboid sensor mask, and u_non_staggered_raw. When running from the k-Wave MATLAB Toolbox V1.1, the generated input files will also be V1.1.

The HDF5 input file for the CPU/GPU simulation code contains a file header with a brief description of the simulation stored in string attributes (see Table 4.2), and the root group '/' which stores all the simulation properties in the form of 3D datasets (a complete list of input datasets is given in Table B.1 in Appendix B). The HDF5 checkpoint file contains the

Table 4.2: The input HDF5 file header

Attribute	Description
`'created_by'`	short description of the tool that created this file
`'creation_date'`	date when the file was created
`'file_description'`	short description of the content of the file (e.g. simulation name)
`'file_type'`	type of the file (`'input'`)
`'major_version'`	major version of the file definition (`'1'`)
`'minor_version'`	minor version of the file definition (`'1 '`)

Table 4.3: The output HDF5 file header

Attribute	Description
`'created_by'`	short description of the tool that created this file
`'creation_date'`	date when the file was created
`'file_description'`	short description of the content of the file (eg. simulation name)
`'file_type'`	type of the file (`'output'`)
`'major_version'`	major version of the file definition (`'1'`)
`'minor_version'`	minor version of the file definition (`'1'`)
`'host_names'`	list of hosts (computer names) the simulation was executed on
`'number_of_cpu_cores'`	number of CPU cores used for the simulation
`'data_loading_phase_execution_time'`	time taken to load data from the file
`'pre-processing_phase_execution_time'`	time taken to pre-process data
`'simulation_phase_execution_time'`	time taken to run the simulation
`'post-processing_phase_execution_time'`	time taken to complete the post-processing phase
`'total_execution_time'`	total execution time
`'peak_core_memory_in_use'`	peak memory per core required during the simulation
`'total_memory_in_use'`	total peak memory in use

same file header as the input file and the root group `'/'` with several additional datasets which store the current simulation state. The HDF5 output file contains a file header with the simulation description as well as performance statistics, such as the simulation time and memory consumption, stored in string attributes (see Table 4.3).

The results of the simulation are stored in the root group `'/'` in the form of 3D/4D datasets. If a binary sensor mask is used, all output quantities are stored as datasets in the root group. If a cuboid corners sensor mask is used, the sampled quantities form private groups containing 4D datasets on a per cuboid basis. A complete list of output datasets is given in Table B.3 in Appendix B.

The input, checkpoint, and output files (for a binary sensor mask) store all quantities as three dimensional datasets with dimension sizes defined by `(Nx, Ny, Nz)`. In order to support scalars and 1D and 2D arrays, the unused dimensions are set to 1. For example, scalar variables are stored with a dimension size of `(1, 1, 1)`, 1D vectors oriented in y-direction are stored with a dimension size of `(1, Ny, 1)`, time varying inputs and outputs are stored with a dimension size of `(Ns, Nt, 1)`, and so on. If the dataset stores a complex variable, the real and imaginary parts are stored in an interleaved layout and the lowest used dimension size is doubled (i.e., `Nx` for a 3D matrix, `Ny` for a 1D vector oriented in the y-direction). The datasets are physically stored in row-major order (in contrast to column-major order used by MATLAB) using either the `'H5T_IEEE_F32LE'` data type for floating point datasets or `'H5T_STD_U64LE'` for integer based datasets. All the datasets are stored under the root group.

For a sensor mask defined by opposing corners of a cuboid, the time varying quantities in the output file are laid out as four dimensional datasets stored under separate groups corresponding to each cuboid. The dimension sizes are defined by `(Nx, Ny, Nz, Nt)`. Every sampled cuboid is stored as a distinct dataset and the datasets are grouped under a group named by the quantity stored. This makes the file clearly readable and easy to parse.

In order to enable compression and more efficient data processing, large datasets are not stored as monolithic blocks but broken into chunks that may be compressed by the ZIP library and stored separately. The chunk size is defined as follows:

- `(1M elements, 1, 1)` in the case of 1D variables - index sensor mask (8MB blocks).
- `(Nx, Ny, 1)` in the case of 3D variables (one 2D slab).
- `(Nx, Ny, Nz, 1)` in the case of 4D variables (one time step).
- `(N_sensor_points, 1, 1)` in the case of the output time series (one time step of the simulation).

All datasets have two attributes that specify the content of the dataset. The `'data_type'` attribute specifies the data type of the dataset. The admissible values are either `'float'` or `'long'`. The `'domain_type'` attribute specifies the domain of the dataset. The admissible values are either `'real'` for the real domain or `'complex'` for the complex domain. The CPU/GPU code reads these attributes and checks their values.

4.7 Compiling the CPU/GPU Source Code in Linux

The source codes of `kpsaceFirstOrder3D-OMP` are written using the C++11 standard and the OpenMP 4.0 library. There are variety of different C++ compilers that can be used to compile the source codes. We recommend using either the GNU C++ compiler (gcc/g++) version 4.9.1 and higher, or the Intel C++ compiler version 15.0 and higher. The codes can be compiled on 64-bit Linux and Windows. 32-bit systems are not supported. This section describes the compilation procedure using GNU and Intel compilers on Linux.

The source codes of `kpsaceFirstOrder3D-CUDA` are written using the C++11 standard, (optional OpenMP 2.0 library) and NVIDIA CUDA 7.5. We recommend using either the GNU C++ compiler (gcc/g++) version 4.8 or 4.9, or the Intel C++ compiler version 15.0. The codes can be compiled on 64-bit Linux and Windows. On Windows, you can only use Visual Studio 2013 (CUDA 7.5 does not support other versions of Visual Studio).

Before compiling the codes, it is necessary to install a C++ compiler and several libraries. The open-source GNU compiler is usually included as part of Linux distributions. It can be downloaded from `http://gcc.gnu.org/` if necessary (to check for the GNU C++ compiler enter `g++ --version` in a terminal window). The Intel compiler can be downloaded from `http://software.intel.com/en-us/intel-composer-xe/` (to check for the Intel C++ compiler enter `icpc --version` in a terminal window). This package also includes the Intel MKL (Math Kernel Library) library that contains the FFT. The Intel compiler is only free for non-commercial use. The GPU code also requires a CUDA compiler, runtime, and library which can be downloaded as a single package from `https://developer.nvidia.com/cuda-toolkit-archive`. The supported versions are 7.0 and 7.5. We cannot guarantee the code can be compiled and work with later versions of CUDA.

The code also relies on several libraries that must be installed before compiling:

1. HDF5 library - version 1.8 or higher (`http://www.hdfgroup.org/HDF5/`). We recommend version 1.8.16. Please do not install any version of 1.10.x, as these are not directly supported by MATLAB yet.

The CPU code also requires an FFT library:

2. FFTW library - version 3.0 or higher (`http://www.fftw.org/`).

and / or

3. MKL library - version 11.0 or higher (`http://software.intel.com/en-us/intel-composer-xe/`).

Although it is possible to use any combination of FFT libraries and compilers for the CPU version, the best performance is observed when using the GNU compiler and FFTW, or the Intel Compiler and Intel MKL. Note, the HDF5 library uses the ZIP library to compress datasets. If this library is not present on your system, it can be installed from the linux repository (e.g., using `sudo apt-get install libz-dev`) and is usually stored under `/usr/lib`.

The HDF5 library installation procedure

1. Download the HDF5 source code for your platform (`http://www.hdfgroup.org/HDF5/release/obtain5.html`).
2. Configure the HDF5 distribution. Enable the high-level library and specify an installation folder by typing:

   ```
   ./configure --enable-hl --prefix=folder_to_install
   ```

3. Make the HDF5 library by typing:

   ```
   make -j
   ```

4. Install the HDF5 library by typing:

   ```
   make install
   ```

The FFTW library installation procedure

1. Download the FFTW library package for your platform (`http://www.fftw.org/download.html`).
2. Configure the FFTW distribution. Enable OpenMP support, SIMD instruction sets, single precision data type, and specify an installation folder:

   ```
   ./configure --enable-single --enable-sse2 --enable-openmp \
               --enable-shared --prefix=folder_to_install
   ```

   If you intend to use the FFTW library (and the CPU code) on the same machine on which the code is being compiled, and want to get the best possible performance, you may also add processor specific optimisations and the AVX/AVX2 instruction set. Note, the compiled binary code is then not likely to be portable on different CPUs (AVX2 will work only on Haswell and later, AVX on Sandy Bridge and later, SSE2 will work on any machine).

   ```
   ./configure --enable-single --enable-avx --enable-openmp \
               --enable-shared --with-gcc-arch=<arch> \
               --prefix=folder_to_install
   ```

   More information about the installation and customization can be found at `http://www.fftw.org/fftw3_doc/Installation-and-Customization.html`.

3. Make the FFTW library by typing:

   ```
   make -j
   ```

4. Install the FFTW library by typing:

   ```
   make install
   ```

The Intel Compiler and MKL installation procedure

1. Download the Intel Composer XE package for your platform (`http://software.intel.com/en-us/intel-compilers`).
2. Run the installation script and follow the procedure by typing:

```
./install.sh
```

The CUDA installation procedure

1. Download CUDA version 7.5 (`https://developer.nvidia.com/cuda-toolkit-archive`)
2. Follow the NVIDIA official installation guide for Windows and Linux (available from `http://docs.nvidia.com/cuda/`)

Compiling the CPU and GPU codes

When the libraries and the compiler have been installed, you are ready to compile the `kspaceFirstOrder3D-OMP` code.

1. Download the `kspaceFirstOrder3D-OMP` source codes from `http://www.k-wave.org/download.php`.
2. Open the `Makefile` file. The Makefile supports code compilation under GNU compiler and FFTW, or Intel compiler with MKL. Uncomment the desired compiler by removing the `'#'` character.

   ```
   #COMPILER = GNU
   #COMPILER = Intel
   ```

   Select how to link the libraries. Static linking is preferred, however, on some systems (e.g., HPC clusters) it may be better to use dynamic linking and use system specific libraries at runtime.

   ```
   #LINKING = STATIC
   #LINKING = DYNAMIC
   ```

   Set the installation paths of the libraries (an example is shown below).

   ```
   FFT_DIR=/usr/local
   MKL_DIR=/opt/intel/composer_xe_2011_sp1/mkl
   HDF5_DIR=/usr/local/hdf5-1.8.16
   ```

   To maximise performance, the code will be built to work only on the same processor architecture as the machine it is being compiled. If you want to create a more general binary, change the CPU architecture according to the compiler documentation.

3. Compile the source code by typing:

   ```
   make -j
   ```

   The compiled binary is called `kspaceFirstOrder3D-OMP`.

4. If you want to clean (delete) the distribution, type:

   ```
   make clean
   ```

The compilation procedure for the GPU code (`kspaceFirstOrder3D-CUDA`) is analogous. In this case, there is only a single compiler possibility (gcc) and the FFTW library is not required.

4.8 Compiling the CPU/GPU Source Code in Windows

The Windows source codes for `kspaceFirstOrder3D-OMP` and `kspaceFirstOrder3D-CUDA` can be compiled using Microsoft Visual Studio 2013. For this purpose, the source code zip file contains a Visual Studio 2013 project. In the case of the CPU code, it is necessary to download and install the Intel C++ Compiler and Intel MKL library. Both can be downloaded as a single package as part of Intel Composer XE from `http://software.intel.com/en-us/Intel-composer-XE-2013-evaluation-options/`. Note, the Intel tools for Windows are not free software, although there is a 30-day evaluation copy.

After installing the Intel Compiler, download and install the HDF5 library from `http://www.hdfgroup.org/HDF5/release/obtain5.html`. The most suitable version is "Windows (64-bit) Compilers: CMake VS 2013 C, C++, IVF 15". After installing HDF5, open the Visual studio solution and change the path to directory where the HDF5 library is installed. Then you're ready to compile the code. Before doing so, check that you're using the Intel Compiler (not the Microsoft Compiler) and switch the build mode to 64-bit mode. The GPU code can be compiled with the default Microsoft Compiler.

In order to run the code, you have to copy all the necessary libraries from the Visual Studio redistribute package to the same folder as the compiled binary. You also need to add the Intel redistributable libraries as well as the HDF5 libraries. These can be found under the installation folders of each particular tool. If you have also downloaded the compiled executable and library files, you can use this to check exactly which library files are required.

4.9 Performance and Memory Usage

Depending on the exact properties of your system, how finely tuned the compiled binaries are, and the simulation domain sizes chosen, the compiled CPU code will typically outperform the MATLAB code on the order of 7 to 12 times. The compiled GPU code typically outperforms the MATLAB code accelerated by GPU (using the Parallel Computing Toolbox) by a factor of 3 to 4.

The execution times per time step for domain sizes growing from 64^3 to 512^3 grid points are shown in Fig. 4.2. The benchmark simulation consisted of p_0 source in a fully heterogeneous and absorbing medium accounting for nonlinear propagation, with the time varying acoustic pressure recorded over a single xy 2D plane. The domain sizes grow with multiples of 32 in order to preserve good memory alignment and keep the domain sizes favourable for the FFT libraries (FFT performance is very sensitive to the domain sizes). This is noticeable in Fig. 4.2, where several drops in execution time can be seen (execution is fasted than expected) for domain sizes that are powers of two or with very low prime factors (2, 3, 5, 7).

The CPU code was benchmarked on five different processors with three different architectures (Sandy Bridge, Ivy Bridge and Haswell). The raw data and details of the system configuration are given in Tables C.1 and C.3 in Appendix C. The CPU binaries for Sandy and Ivy Bridge CPUs were compiled with the AVX instruction set while the binary for

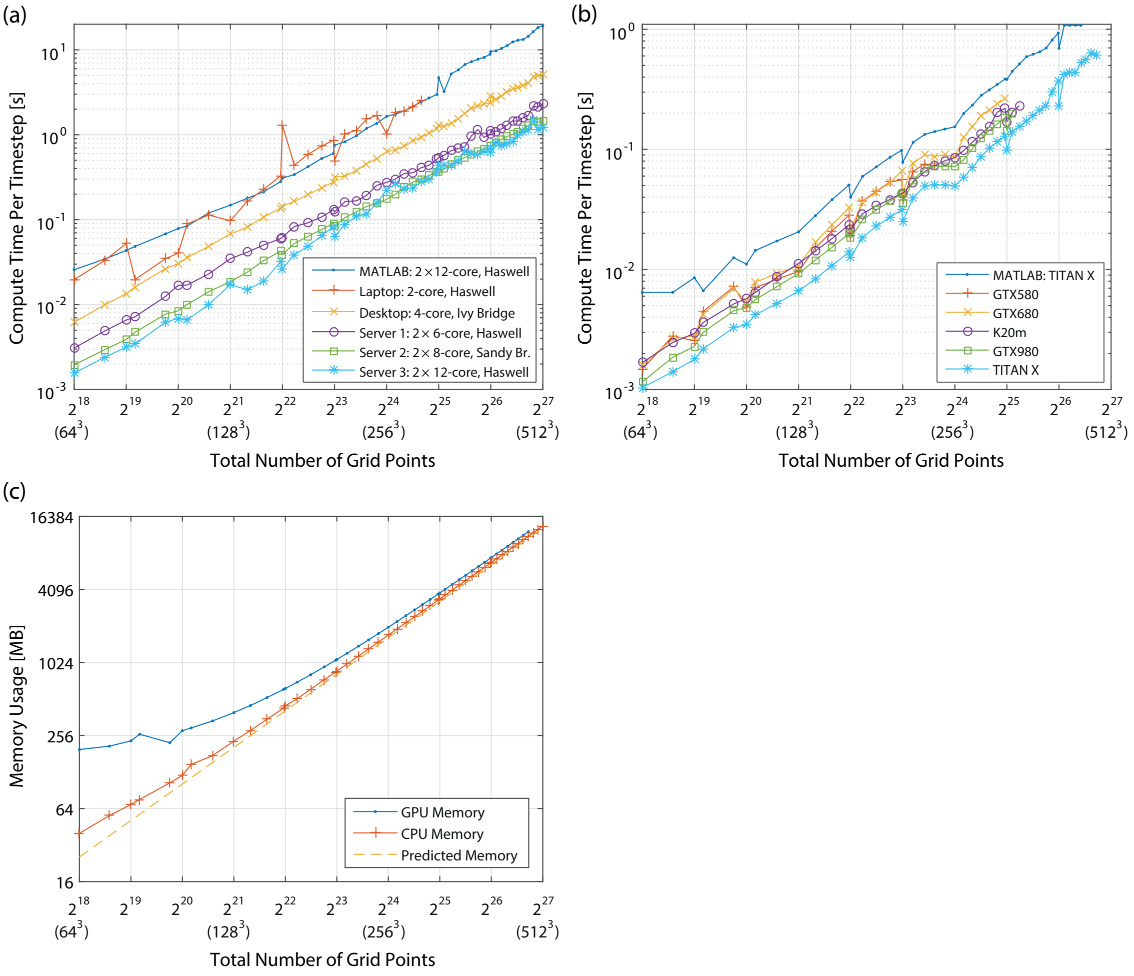

Figure 4.2: Compute times per times step for the nonlinear, heterogeneous absorbing simulation for different 3D grid sizes. (a) The comparison of the CPU code and the MATLAB version on several computers including a laptop, desktop and three servers. (b) The comparison of the GPU code and the GPU-accelerated MATLAB code on a set of high-end Graphics cards. (c) Memory consumption of the CPU and GPU code for the nonlinear, heterogeneous absorbing simulations for different 3D grid sizes.

Haswell was compiled with the AVX2 instruction set. All codes were compiled using Ubuntu 14.04 with the Intel C++ Compiler 2016, MKL 11.3.1, and the platform specific optimisations `-xhost` and `-fast`.

Typical personal computers are represented by a laptop with a dual-core Intel i5 ultra low voltage processor based on the Haswell architecture, and a desktop PC with a quad-core Intel i5 based on the Ivy Bridge architecture. Typical server machines are represented by a dual-socket 6-core Haswell server (Server 1), dual-socket 8-core Sandy Bridge server (Server 2), and a dual-socket 12-core Haswell server (Server 3). The plots are supplemented with the MATLAB version running on the fastest system. Analysis of Fig. 4.2(a) illustrates that the CPU code is memory bound (the laptop features a single memory channel, the desktop has two channels, and the servers are equipped with 2×4 channels at different

speeds). The difference between tested systems is clearly visible and can be used for execution time prediction on similar systems.

To benchmark the GPU code, we used five different high-performance GPUs covering the currently supported GPU architectures, see Fig. 4.2(b). The raw data and details of the system configuration are given in Tables C.2 and C.4 in Appendix C. The code was compiled using Ubuntu 14.04 with NVIDIA CUDA 7.5 and GNU C++ 4.8.5. GTX 580 is the flagship GPU of the Fermi architecture equipped with 1.5 GB of RAM. Although released in 2010, it can still be found in many supercomputer installations. GTX 680 and K20m represent high-end desktop and server models of the Kepler architecture, with 4 and 5 GB of RAM, respectively. The Maxwell architecture is represented by the GTX 980 (a high-end desktop GPU) and a server card TITAN X with 8 and 12 GB of RAM, respectively. From Fig. 4.2(b), it can be seen that the GPU code is also memory bound. The differences in execution time among the tested GPUs is determined primarily by the differences in memory bandwidth. For example, comparing GTX 580 and Maxwell TITAN X, there is an increase in the theoretical memory bandwidth of about 1.7x (192 vs 336 GB/s), while the raw compute power increases by a factor of 7 (1.5 TFLOPS vs 11 TFLOPS). In comparison, the experimental measurements reveal that the TITAN X is usually $1.4 - 2.1$ times faster than the GTX 580, which is on the order of the increase in memory bandwidth. Thus, reasonable performance can be obtained, even on older generation GPUs. The most important parameters are the memory bandwidth and the amount of on-board RAM, which limits the size of simulation domain. More info about the GPUs used can be found at `http://www.geforce.com/hardware/desktop-gpus`.

The approximate memory usage for a particular grid size Nx $\times$ Ny $\times$ Nz (not including variables that are 1D vectors) can be estimated using the formula

$$\text{memory usage [GB]} \approx \frac{(13+A)\,\mathrm{Nx\,Ny\,Nz} + (7+B)\,\frac{\mathrm{Nx}}{2}\,\mathrm{Ny\,Nz}}{1024^3/4} + \text{input} + \text{output}. \quad (4.1)$$

Here the constants $A = [0, 9]$ and $B = [0, 2]$ depend on which material properties are heterogeneous. The parameter "input" is the size of the user defined input data in single precision (for example, the number of elements in `source.p0` or `source.p` times 4 bytes per element). Similarly, "output" is the size of the active elements in the sensor mask (in double precision), plus the number of active elements in the sensor mask times the number of aggregated output variables (in single precision), where the aggregated variables are `p_max`, `p_rms`, etc (not `p` and `u`).

The number 13 in the first term accounts for storing p, ρ_x, ρ_y, ρ_z, u_x, u_y, u_z, $\partial u_x/\partial x$, $\partial u_y/\partial y$, and $\partial u_z/\partial z$ in addition to 3 temporary matrices. A varies from 0 (if the medium is completely homogeneous and `u_non_staggered_raw` is not stored) to 9 (if the simulation is nonlinear, absorbing, completely heterogeneous, and `u_non_staggered_raw` is stored). This accounts for storing c_0 (if `medium.sound_speed` is heterogeneous), ρ_0, $\rho_{0,\mathrm{sgx}}$, $\rho_{0,\mathrm{sgy}}$, $\rho_{0,\mathrm{sgz}}$ (if `medium.density` is heterogeneous and the staggered grid scheme is used), B/A (if `medium.BonA` is given and is heterogeneous), τ, η (if the medium is absorbing and either `medium.alpha_coeff` or `medium.sound_speed` are heterogeneous), and an additional temporary matrix required to perform spatial shifts (if `u_non_staggered_raw` is stored).

The number 7 in the second term accounts for storing κ (which is real) and three temporary

complex matrices. The divisor accounts for the fact that only half the data is stored in the spatial Fourier domain because the real-to-complex FFT in FFTW is used. B is either 0 (if the medium is lossless) or 2 (if the medium is absorbing).

When using the GPU code, CUDA FFTs allocate additional memory to perform FFTs efficiently. The amount of memory used strongly depends on the simulation domain size. For powers of two, it is usually Nx $\times$ Ny $\times$ Nz, meaning one additional matrix is needed. On the contrary, for domain sizes with large prime factors or prime numbers, the additional memory may reach 3 or 4 multiples of the domain size. The reason is that to run the FFTs in a highly parallel manner (using up to millions of threads), some data has to be replicated.

The actual memory consumption for the benchmark CPU and GPU simulations is shown in Fig. 4.2(c), along with the memory consumption predicted using Eq. (4.1). Note that on desktop systems, CPU and GPU memory may be shared with other applications. For example, Firefox can consume up to 1 GB of GPU memory, which can cause simulations to crash due to an out-of-memory exception. Running the GPU code on desktops while being used for other interactive work can also lead to very long input lags and stuttering. This is caused by the fact that GPUs do not support simultaneous multitasking and the CUDA code and the windows manager have to alternate.

Appendix A

List of Optional Input Parameters

Table A.1: List of optional input parameters.

Parameter	Settings	Default	Description
`‘CartInterp’`	`‘linear’`<br>`‘nearest’`	`‘linear’`	Interpolation mode used to extract the pressure when a Cartesian sensor mask is given.
`‘CreateLog’`	*boolean*	`false`	Boolean controlling whether the command line output is saved using the diary function with a date and time stamped filename.
`‘DataCast’`	*string*	`‘off’`	String input of the data type that variables are cast to before computation. Valid inputs are `‘off’`, `‘single’`, `‘gpuArray-single’`, and `‘gpuArray-double’`. GPU inputs require the MATLAB Parallel Computing Toolbox R2012a or later.
`‘DataRecast’`	*boolean*	`false`	Boolean controlling whether the output data is cast back to double precision. If set to false, `sensor_data` will be returned in the data format set using the `‘DataCast’` option.
`‘DisplayMask’`	*binary matrix*<br>`‘off’`	`sensor.mask`	Binary matrix overlayed onto the animated simulation display. Elements set to 1 within the display mask are set to black within the display.

Table A.1: List of optional input parameters continued ...

Parameter	Settings	Default	Description
`'LogScale'`	*boolean scalar*	`false`	Boolean controlling whether the pressure field is log compressed before display.
`'MeshPlot'`[1]	*boolean*	`false`	Boolean controlling whether `mesh` is used in place of `imagesc` to plot the pressure field. When `'MeshPlot'` is set to `true`, the default display mask is set to `'off'`.
`'MovieArgs'`	*cell array*	`{}`	Settings for `movie2avi`. Parameters must be given as `{param, value, ...}` pairs within a cell array.
`'MovieName'`	*string*	`'date-time'`	Name of the movie produced when `'RecordMovie'` is set to true.
`'MovieType'`[1]	`'frame'` `'image'`	`'frame'`	Parameter controlling whether the image frames are captured using `getframe` (`'frame'`) or `im2frame` (`'image'`). If set to `'image'`, the size of the movie will depend on the size of the simulation grid.
`'PlotFreq'`	*integer*	10	The number of iterations which must pass before the simulation plot is updated
`'PlotLayout'`	*boolean*	`false`	Boolean controlling whether plots are produced of the initial simulation layout (initial pressure, sound speed, density).
`'PlotPML'`	*boolean*	`true`	Boolean controlling whether the perfectly matched layer is shown in the simulation plots. If set to false, the PML is not displayed.
`'PlotScale'`	*matrix* `'auto'`	`[-1, 1]`	`[min, max]` values used to control the scaling for imagesc (visualisation). If set to 'auto', a symmetric plot scale is chosen automatically for each plot frame.
`'PlotSim'`	*boolean*	`true`	Boolean controlling whether the simulation iterations are progressively plotted.
`'PMLAlpha'`	*scalar*	2	Absorption within the perfectly matched layer in Nepers per metre.

[1] 2D Simulations Only

Table A.1: List of optional input parameters continued ...

Parameter	Settings	Default	Description
`'PMLInside'`	*boolean*	`true`	Boolean controlling whether the perfectly matched layer is inside or outside the grid. If set to false, the input grids are enlarged by PMLSize before running the simulation.
`'PMLSize'`	*scalar*	`20` (1D, 2D) `10` (3D)	Size of the perfectly matched layer in grid points. By default, the PML is added evenly to all sides of the grid, however, both `PMLSize` and `PMLAlpha` can be given as N element arrays to specify the properties in each Cartesian direction. To remove the PML, set the appropriate `PMLAlpha` to zero rather than forcing the PML to be of zero size.
`'RecordMovie'`	*boolean*	`false`	Boolean controlling whether the displayed image frames are captured and stored as a movie using `movie2avi`.
`'SaveToDisk'`[2]	*string*	`''`	String containing a filename (including pathname if required). If set, after the precomputation phase, the input variables used in the time loop are saved the specified location in HDF5 format. The simulation then exits. The saved variables can be used to run simulations using the C++ code.
`'Smooth'`	*boolean*	`[true, false, false]`	Boolean controlling whether `source.p0`, `medium.sound_speed`, and `medium.density` are smoothed using `smooth` before computation. `'Smooth'` can either be given as a single boolean value, or as a 3 element array to control the smoothing of `source.p0`, `medium.sound_speed`, and `medium.density`, independently.
`'StreamToDisk'`[5]	*boolean* *scalar*	`false`	Boolean controlling whether `sensor_data` is periodically saved to disk to avoid storing the complete matrix in memory. `StreamToDisk` may also be given as an integer which specifies the number of times steps that are taken before the data is saved to disk (default = 200).

[2] 3D Simulations Only

Appendix B

Format of the C++ HDF5 Files

Table B.1: List of datasets that may be present in the input HDF5 file. The dimension sizes are given following the MATLAB indexing convention. Note, MATLAB automatically converts HDF5 files from column-major to row-major ordering. If creating files outside MATLAB, dataset dimensions should be given as `(Nz, Ny, Nx)`.

Name	Size `(Nx, Ny, Nz)`	Data Type	Domain Type	Conditions
1. Simulation Flags				
`ux_source_flag`	`(1, 1, 1)`	`long`	`real`	
`uy_source_flag`	`(1, 1, 1)`	`long`	`real`	
`uz_source_flag`	`(1, 1, 1)`	`long`	`real`	
`p_source_flag`	`(1, 1, 1)`	`long`	`real`	
`p0_source_flag`	`(1, 1, 1)`	`long`	`real`	
`transducer_source_flag`	`(1, 1, 1)`	`long`	`real`	
`nonuniform_grid_flag`	`(1, 1, 1)`	`long`	`real`	must be set to 0
`nonlinear_flag`	`(1, 1, 1)`	`long`	`real`	
`absorbing_flag`	`(1, 1, 1)`	`long`	`real`	
2. Grid Properties				
`Nx`	`(1, 1, 1)`	`long`	`real`	
`Ny`	`(1, 1, 1)`	`long`	`real`	
`Nz`	`(1, 1, 1)`	`long`	`real`	
`Nt`	`(1, 1, 1)`	`long`	`real`	
`dt`	`(1, 1, 1)`	`float`	`real`	
`dx`	`(1, 1, 1)`	`float`	`real`	
`dy`	`(1, 1, 1)`	`float`	`real`	
`dz`	`(1, 1, 1)`	`float`	`real`	

Table B.1: List of datasets that may be present in the input HDF5 file continued ...

Name	Size (Nx, Ny, Nz)	Data Type	Domain Type	Conditions
3. Medium Properties				
3.1. Regular Medium Properties				
rho0	(Nx, Ny, Nz)	float	real	heterogeneous
	(1, 1, 1)	float	real	homogeneous
rho0_sgx	(Nx, Ny, Nz)	float	real	heterogeneous
	(1, 1, 1)	float	real	homogeneous
rho0_sgy	(Nx, Ny, Nz)	float	real	heterogeneous
	(1, 1, 1)	float	real	homogeneous
rho0_sgz	(Nx, Ny, Nz)	float	real	heterogeneous
	(1, 1, 1)	float	real	homogeneous
c0	(Nx, Ny, Nz)	float	real	heterogeneous
	(1, 1, 1)	float	real	homogeneous
c_ref	(1, 1, 1)	float	real	
3.2. Nonlinear Medium Properties (defined if 'nonlinear_flag = 1')				
BonA	(Nx, Ny, Nz)	float	real	heterogeneous
	(1, 1, 1)	float	real	homogeneous
3.3. Absorbing Medium Properties (defined if 'absorbing_flag = 1')				
alpha_coeff	(Nx, Ny, Nz)	float	real	heterogeneous
	(1, 1, 1)	float	real	homogeneous
alpha_power	(1, 1, 1)	float	real	
4. Sensor Properties				
sensor_mask_type	(1, 1, 1)	long	real	
sensor_mask_index	(Nsens, 1, 1)	long	real	sensor_mask_type = 0
sensor_mask_corners	(Ncubes, 6, 1)	long	real	sensor_mask_type = 1
5. Source Properties				
5.1 Velocity Source Terms (defined if ux_source_flag = 1 or uy_source_flag = 1 uz_source_flag = 1)				
u_source_mode	(1, 1, 1)	long	real	
u_source_many	(1, 1, 1)	long	real	
u_source_index	(Nsrc, 1, 1)	long	real	

Table B.1: List of datasets that may be present in the input HDF5 file continued ...

Name	Size (Nx, Ny, Nz)	Data type	Domain Type	Conditions
ux_source_input	(1, Nt_src, 1)	float	real	u_source_many = 0
	(Nsrc, Nt_src, 1)	float	real	u_source_many = 1
uy_source_input	(1, Nt_src, 1)	float	real	u_source_many = 0
	(Nsrc, Nt_src, 1)	float	real	u_source_many = 1
uy_source_input	(1, Nt_src, 1)	float	real	u_source_many = 0
	(Nsrc, Nt_src, 1)	float	real	u_source_many = 1
5.2 Pressure Source Terms (defined if p_source_flag = 1)				
p_source_mode	(1, 1, 1)	long	real	
p_source_many	(1, 1, 1)	long	real	
p_source_index	(Nsrc, 1, 1)	long	real	
p_source_input	(1, Nt_src, 1)	float	real	p_source_many = 0
	(Nsrc, Nt_src, 1)	float	real	p_source_many = 1
5.3 Transducer Source Terms (defined if transducer_source_flag = 1)				
u_source_index	(Nsrc, 1, 1)	long	real	
transducer_source_input	(Nt_src, 1, 1)	float	real	
delay_mask	(Nsrc, 1, 1)	float	real	
5.4 IVP Source Terms (defined if p0_source_flag = 1)				
p0_source_input	(Nx, Ny, Nz)	float	real	
6. k-space and Shift Variables				
ddx_k_shift_pos_r	(Nx/2 + 1, 1, 1)	float	complex	
ddx_k_shift_neg_r	(Nx/2 + 1, 1, 1)	float	complex	
ddy_k_shift_pos	(1, Ny, 1)	float	complex	
ddy_k_shift_neg	(1, Ny, 1)	float	complex	
ddz_k_shift_pos	(1, 1, Nz)	float	complex	
ddz_k_shift_neg	(1, 1, Nz)	float	complex	
x_shift_neg_r	(Nx/2 + 1, 1, 1)	float	complex	
y_shift_neg_r	(1, Ny/2 + 1, 1)	float	complex	
z_shift_neg_r	(1, 1, Nz/2 + 1)	float	complex	

Table B.1: List of datasets that may be present in the input HDF5 file continued ...

Name	Size (Nx, Ny, Nz)	Data type	Domain Type	Conditions
7. PML Variables				
pml_x_size	(1, 1, 1)	long	real	
pml_y_size	(1, 1, 1)	long	real	
pml_z_size	(1, 1, 1)	long	real	
pml_x_alpha	(1, 1, 1)	float	real	
pml_y_alpha	(1, 1, 1)	float	real	
pml_z_alpha	(1, 1, 1)	float	real	
pml_x	(Nx, 1, 1)	float	real	
pml_x_sgx	(Nx, 1, 1)	float	real	
pml_y	(1, Ny, 1)	float	real	
pml_y_sgy	(1, Ny, 1)	float	real	
pml_z	(1, 1, Nz)	float	real	
pml_z_sgz	(1, 1, Nz)	float	real	

Table B.2: List of datasets present in the checkpoint HDF5 file. The dimension sizes are given following the MATLAB indexing convention. Note, MATLAB automatically converts HDF5 files from column-major to row-major ordering. If creating files outside MATLAB, dataset dimensions should be given as (Nz, Ny, Nx).

Name	Size (Nx, Ny, Nz)	Data Type	Domain Type	Conditions
1. Grid Properties				
Nx	(1, 1, 1)	long	real	
Ny	(1, 1, 1)	long	real	
Nz	(1, 1, 1)	long	real	
Nt	(1, 1, 1)	long	real	
t_index	(1, 1, 1)	long	real	
2. Simulation State				
p	(Nx, Ny, Nz)	float	real	
ux_sgx	(Nx, Ny, Nz)	float	real	
uy_sgy	(Nx, Ny, Nz)	float	real	
uz_sgz	(Nx, Ny, Nz)	float	real	
rhox	(Nx, Ny, Nz)	float	real	
rhoy	(Nx, Ny, Nz)	float	real	
rhoz	(Nx, Ny, Nz)	float	real	

Table B.3: List of datasets present in the output HDF5 file. The dimension sizes are given following the MATLAB indexing convention. Note, MATLAB automatically converts HDF5 files from column-major to row-major ordering. If reading files outside MATLAB, dataset dimensions are given as `(Nz, Ny, Nx)`.

Name	Size (Nx, Ny, Nz)	Data type	Domain Type	Conditions
1. Simulation Flags				
ux_source_flag	(1, 1, 1)	long	real	
uy_source_flag	(1, 1, 1)	long	real	
uz_source_flag	(1, 1, 1)	long	real	
p_source_flag	(1, 1, 1)	long	real	
p0_source_flag	(1, 1, 1)	long	real	
transducer_source_flag	(1, 1, 1)	long	real	
nonuniform_grid_flag	(1, 1, 1)	long	real	
nonlinear_flag	(1, 1, 1)	long	real	
absorbing_flag	(1, 1, 1)	long	real	
u_source_mode	(1, 1, 1)	long	real	if u_source
u_source_many	(1, 1, 1)	long	real	if u_source
p_source_mode	(1, 1, 1)	long	real	if p_source
p_source_many	(1, 1, 1)	long	real	if p_source
2. Grid Properties				
Nx	(1, 1, 1)	long	real	
Ny	(1, 1, 1)	long	real	
Nz	(1, 1, 1)	long	real	
Nt	(1, 1, 1)	long	real	
dt	(1, 1, 1)	float	real	
dx	(1, 1, 1)	float	real	
dy	(1, 1, 1)	float	real	
dz	(1, 1, 1)	float	real	
3. PML Variables				
pml_x_size	(1, 1, 1)	long	real	
pml_y_size	(1, 1, 1)	long	real	
pml_z_size	(1, 1, 1)	long	real	
pml_x_alpha	(1, 1, 1)	float	real	
pml_y_alpha	(1, 1, 1)	float	real	
pml_z_alpha	(1, 1, 1)	float	real	
pml_x	(Nx, 1, 1)	float	real	
pml_x_sgx	(Nx, 1, 1)	float	real	
pml_y	(1, Ny, 1)	float	real	
pml_y_sgy	(1, Ny, 1)	float	real	
pml_z	(1, 1, Nz)	float	real	
pml_z_sgz	(1, 1, Nz)	float	real	

Table B.3: List of datasets that may be present in the output HDF5 file continued ...

Name	Size (Nx, Ny, Nz)	Data type	Domain Type	Conditions
4. Sensor Variables (defined if `--copy_sensor_mask`)				
`sensor_mask_type`	`(1, 1, 1)`	`long`	`real`	
`sensor_mask_index`	`(Nsens, 1, 1)`	`long`	`real`	`sensor_mask_type = 0`
`sensor_mask_corners`	`(Ncubes, 6, 1)`	`long`	`real`	`sensor_mask_type = 1`
5. Simulation Results				
5.1 Binary Sensor Mask (defined if `sensor_mask_type = 0`)				
`p`	`(Nsens, Nt-s+1, 1)`	`float`	`real`	`-p` or `--p_raw`
`p_rms`	`(Nsens, 1, 1)`	`float`	`real`	`--p_rms`
`p_max`	`(Nsens, 1, 1)`	`float`	`real`	`--p_max`
`p_min`	`(Nsens, 1, 1)`	`float`	`real`	`--p_min`
`p_max_all`	`(Nx, Ny, Nz)`	`float`	`real`	`--p_max_all`
`p_min_all`	`(Nx, Ny, Nz)`	`float`	`real`	`--p_min_all`
`p_final`	`(Nx, Ny, Nz)`	`float`	`real`	`--p_final`
`ux`	`(Nsens, Nt-s+1, 1)`	`float`	`real`	`-u` or `--u_raw`
`uy`	`(Nsens, Nt-s+1, 1)`	`float`	`real`	`-u` or `--u_raw`
`uz`	`(Nsens, Nt-s+1, 1)`	`float`	`real`	`-u` or `--u_raw`
`ux_non_staggered`	`(Nsens, Nt-s+1, 1)`	`float`	`real`	`--u_non_staggered`
`uy_non_staggered`	`(Nsens, Nt-s+1, 1)`	`float`	`real`	`--u_non_staggered`
`uz_non_staggered`	`(Nsens, Nt-s+1, 1)`	`float`	`real`	`--u_non_staggered`
`ux_rms`	`(Nsens, 1, 1)`	`float`	`real`	`--u_rms`
`uy_rms`	`(Nsens, 1, 1)`	`float`	`real`	`--u_rms`
`uz_rms`	`(Nsens, 1, 1)`	`float`	`real`	`--u_rms`
`ux_max`	`(Nsens, 1, 1)`	`float`	`real`	`--u_max`
`uy_max`	`(Nsens, 1, 1)`	`float`	`real`	`--u_max`
`uz_max`	`(Nsens, 1, 1)`	`float`	`real`	`--u_max`
`ux_min`	`(Nsens, 1, 1)`	`float`	`real`	`--u_min`
`uy_min`	`(Nsens, 1, 1)`	`float`	`real`	`--u_min`
`uz_min`	`(Nsens, 1, 1)`	`float`	`real`	`--u_min`

Table B.3: List of datasets that may be present in the output HDF5 file continued . . .

Name	Size (Nx, Ny, Nz)	Data type	Domain Type	Conditions
ux_max_all	(Nx, Ny, Nz)	float	real	--u_max_all
uy_max_all	(Nx, Ny, Nz)	float	real	--u_max_all
uz_max_all	(Nx, Ny, Nz)	float	real	--u_max_all
ux_min_all	(Nx, Ny, Nz)	float	real	--u_min_all
uy_min_all	(Nx, Ny, Nz)	float	real	--u_min_all
uz_min_all	(Nx, Ny, Nz)	float	real	--u_min_all
ux_final	(Nx, Ny, Nz)	float	real	--u_final
uy_final	(Nx, Ny, Nz)	float	real	--u_final
uz_final	(Nx, Ny, Nz)	float	real	--u_final
5.2 Opposing Cuboid Corners Sensor Mask (defined if sensor_mask_type = 1)				
Note, each output group (e.g., /p) contains a dataset for each cuboid defined in sensor_mask_corners, where /1 indicates the first dataset, /2 indicates the second dataset, and so on up to Ncubes.				
p/1	(Cx, Cy, Cz, Nt-s+1)	float	real	-p or --p_raw
p_rms/1	(Cx, Cy, Cz, Nt-s+1)	float	real	--p_rms
p_max/1	(Cx, Cy, Cz, Nt-s+1)	float	real	--p_max
p_min/1	(Cx, Cy, Cz, Nt-s+1)	float	real	--p_min
p_max_all	(Nx, Ny, Nz)	float	real	--p_max_all
p_min_all	(Nx, Ny, Nz)	float	real	--p_min_all
p_final	(Nx, Ny, Nz)	float	real	--p_final
ux/1	(Cx, Cy, Cz, Nt-s+1)	float	real	-u or--u_raw
uy/1	(Cx, Cy, Cz, Nt-s+1)	float	real	-u or--u_raw
uz/1	(Cx, Cy, Cz, Nt-s+1)	float	rea	-u or--u_raw
ux_non_staggered/1	(Cx, Cy, Cz, Nt-s+1)	float	real	--u_non_staggered_raw
uy_non_staggered/1	(Cx, Cy, Cz, Nt-s+1)	float	real	--u_non_staggered_raw
uz_non_staggered/1	(Cx, Cy, Cz, Nt-s+1)	float	real	--u_non_staggered_raw
ux_rms/1	(Cx, Cy, Cz, Nt-s+1)	float	real	--u_rms
uy_rms/1	(Cx, Cy, Cz, Nt-s+1)	float	real	--u_rms
uz_rms/1	(Cx, Cy, Cz, Nt-s+1)	float	real	--u_rms

Table B.3: List of datasets that may be present in the output HDF5 file continued ...

Name	Size (Nx, Ny, Nz)	Data type	Domain Type	Conditions
ux_max/1	(Cx, Cy, Cz, Nt-s+1)	float	real	--u_max
uy_max/1	(Cx, Cy, Cz, Nt-s+1)	float	real	--u_max
uz_max/1	(Cx, Cy, Cz, Nt-s+1)	float	real	--u_max
ux_min/1	(Cx, Cy, Cz, Nt-s+1)	float	real	--u_min
uy_min/1	(Cx, Cy, Cz, Nt-s+1)	float	real	--u_min
uz_min/1	(Cx, Cy, Cz, Nt-s+1)	float	real	--u_min
ux_max_all	(Nx, Ny, Nz)	float	real	--u_max_all
uy_max_all	(Nx, Ny, Nz)	float	real	--u_max_all
uz_max_all	(Nx, Ny, Nz)	float	real	--u_max_all
ux_min_all	(Nx, Ny, Nz)	float	real	--u_min_all
uy_min_all	(Nx, Ny, Nz)	float	real	--u_min_all
uz_min_all	(Nx, Ny, Nz)	float	real	--u_min_all
ux_final	(Nx, Ny, Nz)	float	real	--u_final
uy_final	(Nx, Ny, Nz)	float	real	--u_final
uz_final	(Nx, Ny, Nz)	float	real	--u_final

Appendix C

Performance Evaluation of the CPU and GPU code

Table C.1: Hardware configuration of systems used to benchmark the CPU code.

	Processor			Memory	
System	CPU name	Cores	Frequency	Capacity	Speed
Laptop	i5-4200U	2	1.6 GHz	1×4 GB	1.60 GHz
Desktop	i5-3570	4	3.4 GHz	2×16 GB	1.60 GHz
Server 1	2xE5-2620v3	2x6	2.4 GHz	8×8 GB	2.13 GHz
Server 2	2xE5-2665	2x8	2.4 GHz	8×8 GB	1.60 GHz
Server 3	2xE5-2680v3	2x12	2.5 GHz	8×16 GB	2.13 GHz

Table C.2: Hardware configuration of graphics cards used to benchmark the GPU code.

	Processor			Memory		
GPU Name	Architecture	Cores	Frequency	Capacity	Width	Speed
GTX 580	Fermi	512	1560 MHz	1.5 GB	384 b	2 GHz
GTX 680	Kepler	1535	1005 MHz	4 GB	256 b	6 GHz
K20m	Kepler	2496	706 MHz	5.1 GB	320 b	5.2 GHz
GTX 980	Maxwell	2048	1126 MHz	8 GB	256 b	7 GHz
TITAN X	Maxwell	3072	1000 MHz	12 GB	384 b	7 GHz

Table C.3: Execution time per time step [ms] and memory requirements [MB] of the CPU code for nonlinear heterogeneous absorbing simulations for different 3D grid sizes ranging from 64^3 to 512^3. System configuration is shown in Table C.1.

Domain Size	MATLAB 24 cores	Laptop 2 cores	Desktop 4 cores	Server 1 12 cores	Server 2 16 cores	Server 3 24 cores	Memory [MB]
$64 \times 64 \times 64$	25.76	19.70	6.20	3.08	1.95	1.56	40
$96 \times 64 \times 64$	34.39	33.00	9.90	4.88	2.94	2.40	56
$128 \times 64 \times 64$	43.44	52.40	13.50	6.58	3.89	3.15	69
$96 \times 96 \times 64$	48.45	19.70	16.10	7.19	4.73	3.44	76
$96 \times 96 \times 96$	68.14	34.80	26.60	12.50	7.72	6.21	104
$128 \times 128 \times 64$	78.71	40.40	30.30	16.92	8.47	6.91	120
$128 \times 96 \times 96$	84.46	88.90	35.70	17.02	10.01	6.61	148
$128 \times 128 \times 96$	119.9	113.5	48.60	22.99	14.20	10.04	174
$128 \times 128 \times 128$	148.5	97.30	68.00	34.72	18.70	17.17	230
$160 \times 128 \times 128$	178.5	164.0	81.10	41.35	24.02	15.01	281
$160 \times 160 \times 128$	214.1	228.7	107.6	50.53	33.11	18.90	348
$160 \times 160 \times 160$	281.6	327.9	136.6	59.87	42.61	31.96	430
$256 \times 128 \times 128$	308.9	1296	144.1	62.17	38.86	26.57	450
$192 \times 160 \times 160$	339.7	439.1	164.0	83.23	52.49	38.73	513
$192 \times 192 \times 160$	421.5	589.1	197.9	92.17	63.71	49.05	615
$192 \times 192 \times 192$	525.8	733.3	237.9	108.0	76.63	64.73	737
$224 \times 192 \times 192$	602.0	864.8	276.3	131.0	91.09	83.22	854
$256 \times 256 \times 128$	753.0	493.2	317.9	122.6	85.11	63.99	868
$224 \times 224 \times 192$	826.0	1022	323.9	163.6	105.8	87.04	992
$224 \times 224 \times 224$	975.0	1130	380.5	166.9	124.3	110.6	1157
$256 \times 224 \times 224$	1187	1524	456.0	193.4	142.9	115.2	1319
$256 \times 256 \times 224$	1357	1690	523.4	253.2	155.1	156.1	1506
$256 \times 256 \times 256$	1646	1023	631.0	274.7	174.0	224.5	1717
$288 \times 256 \times 256$	1748	1850	667.5	302.2	199.4	264.4	1931
$288 \times 288 \times 256$	1911	1913	730.0	350.2	239.8	232.0	2168
$288 \times 288 \times 288$	2166	2120	846.5	358.1	278.2	237.2	2436
$320 \times 288 \times 288$	2431	2538	940.9	409.6	302.4	278.7	2704
$320 \times 320 \times 288$	2710		1042	438.2	332.1	300.9	3005
$320 \times 320 \times 320$	2980		1166	514.1	362.7	368.2	3337
$512 \times 256 \times 256$	4697		1297	536.9	364.2	435.8	3416
$352 \times 320 \times 320$	3225		1245	573.5	394.6	431.1	3667
$352 \times 352 \times 320$	5212		1383	649.8	452.4	437.9	4034
$352 \times 352 \times 352$	5814		1542	690.7	500.2	494.8	4435
$384 \times 352 \times 352$	6720		1775	725.6	539.8	632.1	4836
$384 \times 384 \times 352$	7246		2045	971.3	586.3	607.7	5274
$384 \times 384 \times 384$	7738		2191	1154	638.4	582.6	5750
$416 \times 384 \times 384$	8136		2300	928.4	684.5	642.9	6229
$416 \times 416 \times 384$	8877		2386	1011	748.4	704.9	6745
$512 \times 512 \times 256$	9526		2844	1103	732.2	627.7	6811
$416 \times 416 \times 416$	9740		2619	1097	818.5	805.2	7308
$448 \times 416 \times 416$	10392		2859	1177	889.6	749.4	7868
$448 \times 448 \times 416$	11232		3207	1304	952.0	790.4	8469
$448 \times 448 \times 448$	12383		3457	1462	1016	839.1	9121
$480 \times 448 \times 448$	12969		3558	1442	1072	1007	9771
$480 \times 480 \times 448$	13233		3808	1591	1168	1071	10468
$480 \times 480 \times 480$	14403		4098	1694	1279	1158	11214
$512 \times 480 \times 480$	16324		4834	2164	1446	1483	11960
$512 \times 512 \times 480$	18293		4985	2119	1404	1145	12754
$512 \times 512 \times 512$	19265		5160	2326	1462	1207	13605

Table C.4: Execution time per time step [ms] and memory requirements [MB] of the GPU code for nonlinear heterogeneous absorbing simulations for different 3D grid sizes ranging from 64^3 to 480^3. System configuraiton is shown in Table C.2.

Domain size	MATLAB[1]	GTX580	GTX680	K20m	GTX980	TITAN X	Memory
$64 \times 64 \times 64$	6.44	1.48	1.61	1.68	1.16	1.04	196
$96 \times 64 \times 64$	6.46	2.80	2.65	2.47	1.86	1.41	209
$128 \times 64 \times 64$	8.54	2.55	2.81	2.94	2.29	1.79	231
$96 \times 96 \times 64$	6.65	4.48	4.26	3.62	3.00	2.19	262
$96 \times 96 \times 96$	12.53	7.17	6.93	5.17	4.54	3.28	223
$128 \times 128 \times 64$	11.09	4.88	5.41	5.72	4.81	3.48	280
$128 \times 96 \times 96$	14.42	7.06	7.84	6.44	5.62	4.19	295
$128 \times 128 \times 96$	17.28	8.30	9.29	8.63	7.21	5.23	337
$128 \times 128 \times 128$	20.60	9.65	10.70	11.23	9.26	6.62	395
$160 \times 128 \times 128$	28.05	15.11	16.88	14.22	12.00	8.36	452
$160 \times 160 \times 128$	38.19	20.86	23.62	18.13	15.33	10.83	525
$160 \times 160 \times 160$	50.52	28.29	32.57	23.49	19.87	13.91	614
$256 \times 128 \times 128$	40.00	18.96	21.44	21.68	18.22	12.68	624
$192 \times 160 \times 160$	59.55	37.29	36.77	29.14	25.90	18.47	703
$192 \times 192 \times 160$	71.51	44.80	44.21	33.95	31.20	22.89	810
$192 \times 192 \times 192$	85.98	53.58	52.99	38.27	37.02	27.35	940
$224 \times 192 \times 192$	98.18	55.84	65.82	43.31	42.61	31.29	1068
$256 \times 256 \times 128$	78.06	37.93	43.10	43.05	35.97	25.03	1081
$224 \times 224 \times 192$	114.5	65.64	76.71	53.21	54.77	39.52	1218
$224 \times 224 \times 224$	133.3	75.77	89.87	65.48	69.91	49.47	1394
$256 \times 224 \times 224$	140.9	73.36	87.62	73.32	72.21	50.80	1568
$256 \times 256 \times 224$	147.6		90.14	80.36	72.36	50.48	1768
$256 \times 256 \times 256$	154.3		86.48	85.59	71.81	49.72	1996
$288 \times 256 \times 256$	199.0		126.7	97.63	82.08	58.41	2225
$288 \times 288 \times 256$	234.1		154.6	115.0	101.7	70.98	2483
$288 \times 288 \times 288$	282.6		191.7	134.9	123.8	87.31	2772
$320 \times 288 \times 288$	313.0		213.9	153.7	144.4	102.5	3061
$320 \times 320 \times 288$	347.4		239.8	204.5	162.8	115.3	3382
$320 \times 320 \times 320$	385.8		264.1	219.5	184.0	129.7	3740
$512 \times 256 \times 256$	382.7			169.3	142.3	98.71	3824
$352 \times 320 \times 320$	446.7		315.3	203.6	199.2	140.8	4097
$352 \times 352 \times 320$	514.7			232.0		155.5	4489
$352 \times 352 \times 352$	591.4					172.3	4922
$384 \times 352 \times 352$	620.0					190.3	5354
$384 \times 384 \times 352$	649.1					211.5	5826
$384 \times 384 \times 384$	698.9					230.8	6340
$416 \times 384 \times 384$	815.5					304.4	6854
$416 \times 416 \times 384$	929.7					374.2	7411
$512 \times 512 \times 256$	694.3					231.7	7483
$416 \times 416 \times 416$	1079					418.8	8015
$448 \times 416 \times 416$	1079					435.9	8619
$448 \times 448 \times 416$	1082					433.9	9269
$448 \times 448 \times 448$	1079					530.3	9969
$480 \times 448 \times 448$						563.0	10669
$480 \times 480 \times 448$						631.6	11419
$480 \times 480 \times 480$						605.5	12223

[1] Measured on TITAN X

Bibliography

[1] B. E. Treeby and B. T. Cox, “k-Wave: MATLAB toolbox for the simulation and reconstruction of photoacoustic wave fields,” *J. Biomed. Opt.*, vol. 15, no. 2, p. 021314, 2010.

[2] B. E. Treeby, J. Jaros, A. P. Rendell, and B. T. Cox, “Modeling nonlinear ultrasound propagation in heterogeneous media with power law absorption using a k-space pseudospectral method,” *J. Acoust. Soc. Am.*, vol. 131, no. 6, pp. 4324–4336, 2012.

[3] P. Beard, “Biomedical photoacoustic imaging,” *Interface Focus*, vol. 1, no. 4, pp. 602–631, 2011.

[4] B. E. Treeby, J. Jaros, D. Rohrbach, and B. T. Cox, “Modelling Elastic Wave Propagation Using the k-Wave MATLAB Toolbox,” in *IEEE Int. Ultrasonics Symposium*, pp. 146–149, 2014.

[5] A. D. Pierce, *Acoustics: An Introduction to its Physical Principles and Applications.* New York: Acoustical Society of America, 1989.

[6] M. J. Buckingham, “Theory of acoustic attenuation, dispersion, and pulse propagation in unconsolidated granular materials including marine sediments,” *The Journal of the Acoustical Society of America*, vol. 102, no. 5, p. 2579, 1997.

[7] J. C. Bamber, “Attenuation and Absorption,” in *Physical Principles of Medical Ultrasound* (C. R. Hill, J. C. Bamber, and G. R. ter Haar, eds.), pp. 93–166, Chichester: John Wiley, 2004.

[8] M. F. Insana and D. G. Brown, “Acoustic scattering theory applied to soft biological tissues,” in *Ultrasonics Scattering in Biological Tissue* (K. K. Shung and G. A. Thieme, eds.), pp. 75–124, Boca Raton: CRC Press, 1993.

[9] A. Pierce, “Mathematical Theory Of Wave Propagation,” in *Handbook of Acoustics* (M. Crocker, ed.), ch. 2, pp. 21–37, Wiley-IEEE, 1998.

[10] K. Waters, J. Mobley, and J. Miller, “Causality-imposed (Kramers-Kronig) relationships between attenuation and dispersion,” *IEEE Transactions on Ultrasonics, Ferroelectrics and Frequency Control*, vol. 52, no. 5, pp. 822–833, 2005.

[11] W. Chen and S. Holm, “Fractional Laplacian time-space models for linear and nonlinear lossy media exhibiting arbitrary frequency power-law dependency,” *The Journal of the Acoustical Society of America*, vol. 115, no. 4, p. 1424, 2004.

[12] B. E. Treeby and B. T. Cox, “Modeling power law absorption and dispersion for acoustic propagation using the fractional Laplacian,” *J. Acoust. Soc. Am.*, vol. 127, no. 5, pp. 2741–2748, 2010.

[13] B. E. Treeby and B. T. Cox, “Modeling power law absorption and dispersion in viscoelastic solids using a split-field and the fractional Laplacian,” *J. Acoust. Soc. Am.*, vol. 136, no. 4, pp. 1499–1510, 2014.

[14] B. E. Treeby and B. T. Cox, "A k-space Green's function solution for acoustic initial value problems in homogeneous media with power law absorption," *J. Acoust. Soc. Am.*, vol. 129, no. 6, pp. 3652–3660, 2011.

[15] M. F. Hamilton and D. T. Blackstock, eds., *Nonlinear Acoustics.* Melville: Acoustical Society of America, 2008.

[16] B. E. Treeby, M. Tumen, and B. T. Cox, "Time domain simulation of harmonic ultrasound images and beam patterns in 3D using the k-space pseudospectral method," in *Medical Image Computing and Computer-Assisted Intervention, Part I*, vol. 6891, pp. 363–370, Springer, Heidelberg, 2011.

[17] R. T. Beyer, "The parameter B/A," in *Nonlinear Acoustics* (M. F. Hamilton and D. T. Blackstock, eds.), pp. 25–39, Melville: Acoustical Society of America, 2008.

[18] M. F. Hamilton and D. T. Blackstock, "On the coefficient of nonlinearity beta in nonlinear acoustics," *J. Acoust. Soc. Am.*, vol. 83, no. 1, pp. 74–77, 1988.

[19] P. Westervelt, "Parametric acoustic array," *The Journal of the acoustical society of America*, vol. 35, no. 4, pp. 535–537, 1963.

[20] G. Taraldsen, "A generalized Westervelt equation for nonlinear medical ultrasound," *The Journal of the Acoustical Society of America*, vol. 109, no. 4, p. 1329, 2001.

[21] P. Filippi, D. Habault, J.-P. Lefebvre, and A. Bergassoli, *Acoustics: Basic Physics, Theory and Methods.* London: Academic Press, 1999.

[22] F. J. Fahy, *Sound Intensity.* Barking, Essex: Elsevier Applied Science, 1989.

[23] B. T. Cox and P. C. Beard, "Fast calculation of pulsed photoacoustic fields in fluids using k-space methods," *J. Acoust. Soc. Am.*, vol. 117, no. 6, pp. 3616–3627, 2005.

[24] N. N. Bojarski, "The k-space formulation of the scattering problem in the time domain," *J. Acoust. Soc. Am.*, vol. 72, no. 2, pp. 570–584, 1982.

[25] N. N. Bojarski, "The k-space formulation of the scattering problem in the time domain: An improved single propagator formulation," *The Journal of the Acoustical Society of America*, vol. 77, no. 3, p. 826, 1985.

[26] T. D. Mast, L. P. Souriau, D. L. Liu, M. Tabei, a. I. Nachman, and R. C. Waag, "A k-space method for large-scale models of wave propagation in tissue.," *IEEE Transactions on Ultrasonics, Ferroelectrics and Frequency Control*, vol. 48, no. 2, pp. 341–54, 2001.

[27] M. Tabei, T. D. Mast, and R. C. Waag, "A k-space method for coupled first-order acoustic propagation equations," *J. Acoust. Soc. Am.*, vol. 111, no. 1, pp. 53–63, 2002.

[28] B. Fornberg, "Generation of finite difference formulas on arbitrarily spaced grids," *Math. Comput*, vol. 51, no. 184, pp. 699–706, 1988.

[29] B. Fornberg, "The pseudospectral method: Comparisons with finite differences for the elastic wave equation," *Geophysics*, vol. 52, no. 4, pp. 483–501, 1987.

[30] B. T. Cox, S. Kara, S. R. Arridge, and P. C. Beard, "k-space propagation models for acoustically heterogeneous media: Application to biomedical photoacoustics," *J. Acoust. Soc. Am.*, vol. 121, no. 6, pp. 3453–3464, 2007.

[31] B. Fornberg, "High-Order Finite Differences and the Pseudospectral Method on Staggered Grids," *SIAM Journal on Numerical Analysis*, vol. 27, no. 4, pp. 904–918, 1990.

[32] L. N. Trefethen, *Spectral Methods in Matlab.* Philadelphia: SIAM, 2000.

[33] D. Gottlieb and E. Tadmor, "The CFL condition for spectral approximations to hyperbolic initial-boundary value problems," *Mathematics of Computation*, vol. 56, no. 194, pp. 565–588, 1991.

[34] D. Gottlieb and J. S. Hesthaven, "Spectral methods for hyperbolic problems," *Journal of Computational and Applied Mathematics*, vol. 128, no. 1, pp. 83–131, 2001.

[35] J. P. Boyd, *Chebyshev and Fourier Spectral Methods.* Mineola, New York: Dover Publications, 2001.

[36] J. C. Tillett, M. I. Daoud, J. C. Lacefield, and R. C. Waag, "A k-space method for acoustic propagation using coupled first-order equations in three dimensions," *J. Acoust. Soc. Am.*, vol. 126, no. 3, pp. 1231–1244, 2009.

[37] J. Jaros, B. E. Treeby, and A. P. Rendell, "Use of multiple GPUs on shared memory multiprocessors for ultrasound propagation simulations," in *10th Australasian Symposium on Parallel and Distributed Computing* (J. Chen and R. Ranjan, eds.), vol. 127, pp. 43–52, ACS, 2012.

[38] F. A. Duck, *Physical Properties of Tissue: A Comprehensive Reference Book.* Academic Press, 1990.

[39] M. Caputo, "Linear models of dissipation whose Q is almost frequency independent-II," *Geophys. J. R. Astr. Soc.*, vol. 13, pp. 529–539, 1967.

[40] T. Szabo, "Time domain wave equations for lossy media obeying a frequency power law," *The Journal of the Acoustical Society of America*, vol. 96, no. 1, pp. 491–500, 1994.

[41] W. Chen and S. Holm, "Physical interpretation of fractional diffusion-wave equation via lossy media obeying frequency power law," *Arxiv preprint math-ph/0303040*, no. March, 2003.

[42] M. Liebler, S. Ginter, T. Dreyer, and R. E. Riedlinger, "Full wave modeling of therapeutic ultrasound: Efficient time-domain implementation of the frequency power-law attenuation," *J. Acoust. Soc. Am.*, vol. 116, no. 5, pp. 2742–2750, 2004.

[43] M. G. Wismer, "Finite element analysis of broadband acoustic pulses through inhomogenous media with power law attenuation," *J. Acoust. Soc. Am.*, vol. 120, no. 6, pp. 3493–3502, 2006.

[44] J. F. Kelly, R. J. McGough, and M. M. Meerschaert, "Analytical time-domain Green's functions for power-law media.," *The Journal of the Acoustical Society of America*, vol. 124, no. 5, pp. 2861–72, 2008.

[45] A. Nachman, J. Smith III, and R. Waag, "An equation for acoustic propagation in an inhomogeneous medium with relaxation loss," *The Journal of the Acoustical Society of America*, vol. 88, no. 3, pp. 1584–1595, 1990.

[46] I. Podlubny, *Fractional Differential Equations.* New York: Academic Press, 1999.

[47] J.-P. Berenger, "A perfectly matched layer for the absorption of electromagnetic waves," *J. Comput. Phys.*, vol. 114, no. 2, pp. 185–200, 1994.

[48] X. Yuan, S. Member, D. Borup, J. W. Wiskin, M. Berggren, R. Eidens, and S. A. Johnson, "Formulation and Validation of Berengers PML Absorbing Boundary for the FDTD Simulation of Acoustic Scattering," *IEEE Trans. Ultrason. Ferroelectr. Freq. Control*, vol. 44, no. 4, pp. 816–822, 1997.

[49] J.-P. Berenger, "Three-dimensional perfectly matched layer for the absorption of electromagnetic waves," *J. Comput. Phys.*, vol. 127, no. 2, pp. 363–379, 1996.

[50] X. Yuan, D. Borup, J. Wiskin, M. Berggren, and S. A. Johnson, "Simulation of Acoustic Wave Propagation in Dispersive Media with Relaxation Losses by Using FDTD Method with PML Absorbing Boundary Condition," *IEEE Trans. Ultrason. Ferroelectr. Freq. Control*, vol. 46, no. 1, pp. 14–23, 1999.

[51] J. L. Robertson, B. T. Cox, and B. E. Treeby, "Quantifying numerical errors in the simulation of transcranial ultrasound using pseudospectral methods," in *IEEE International Ultrasonics Symposium*, pp. 2000–2003, IEEE, 2014.

[52] M. Brio, A. R. Zakharian, and G. M. Webb, *Numerical Time-Dependent Partial Differential Equations for Scientists and Engineers.* Burlington: Elsevier, 2010.

[53] J. S. Hesthaven, S. Gottlieb, and D. Gottlieb, *Spectral Methods for Time-Dependent Problems.* Cambridge: Cambridge University Press, 2007.

[54] M. Frigo and S. G. Johnson, "The design and implementation of FFTW3," *Proc. IEEE*, vol. 93, no. 2, pp. 216–231, 2005.

[55] B. E. Treeby, E. Z. Zhang, and B. T. Cox, "Photoacoustic tomography in absorbing acoustic media using time reversal," *Inverse Probl.*, vol. 26, no. 11, p. 115003, 2010.

[56] B. E. Treeby, T. K. Varslot, E. Z. Zhang, J. G. Laufer, and P. C. Beard, "Automatic sound speed selection in photoacoustic image reconstruction using an autofocus approach," *Journal of Biomedical Optics*, vol. 16, no. 9, p. 090501, 2011.

[57] B. T. Cox and B. E. Treeby, "Effect of sensor directionality on photoacoustic imaging: A study using the k-Wave toolbox," in *Proc. SPIE*, vol. 7564, p. 75640I, 2010.

[58] O. T. Von Ramm and S. W. Smith, "Beam steering with linear arrays," *IEEE Trans. Biomed. Eng.*, vol. BME-30, no. 8, pp. 438–452, 1983.

[59] V. W. Lee, C. Kim, J. Chhugani, M. Deisher, D. Kim, A. D. Nguyen, N. Satish, M. Smelyanskiy, S. Chennupaty, P. Hammarlund, R. Singhal, and P. Dubey, "Debunking the 100X GPU vs. CPU Myth: An evaluation of throughput computing on CPU and GPU," in *Proceedings of the 37th annual international symposium on Computer architecture*, pp. 451–460, 2010.

[60] J. Jaros and P. Pospichal, "A fair comparison of modern CPUs and GPUs running the genetic algorithm under the knapsack benchmark," in *Applications of Evolutionary Computation*, vol. 7248, pp. 426–435, 2012.
