# Supplementary material for: Asynchronous magnetic resonance elastography: Shear wave speed reconstruction using noise correlation of incoherent waves
Source: Magn Reson Med. 2022 Oct 27;89(3):990–1001. doi: 10.1002/mrm.29502 (PMC9792433; doi:10.1002/mrm.29502)
Supplement: Supplementary file 1 — DATA S1 MATLAB code used to generate the numerical simulations of Figure 1 [file MRM-89-990-s002.zip › k-Wave/helpfiles/addNoise.html]

addNoise :: Functions (k-Wave)


# addNoise

Add Gaussian noise to a signal for a given SNR.

## Syntax

```
signal = addNoise(signal, snr)
signal = addNoise(signal, snr, mode)
[signal, snr] = addNoise(signal, snr)
[signal, snr] = addNoise(signal, snr, mode)
```

## Description

`addNoise` adds Gaussian random noise to a one dimensional input signal given the desired signal snr (signal to noise ratio) in decibels. By default, the magnitude of the added noise is calculated based on the RMS level of the input signal. For impulsive signals, the optional input mode should be set to `'peak'` so the magnitude of the added noise is calculated based on the peak level of the input signal. An example of adding noise to a sinusoidal signal is given below.

```
% specify signal properties
Fs = 50e6;          % [Hz]
dt = 1 / Fs;        % [s]
t = 0:dt:200 * dt;  % [s]

% create signal
signal = sin(0.5e6 * 2 * pi * t);

% add noise to give an snr of 20 dB and 10 dB
noisy_signal_a = addNoise(signal, 20);
noisy_signal_b = addNoise(signal, 10);

% plot
figure;
plot(t * 1e6, noisy_signal_a, 'r-', t * 1e6, noisy_signal_b, 'b-', t * 1e6, signal, 'k-');
xlabel('Time [\mus]');
ylabel('Signal [au]');
legend('20dB SNR', '10dB SNR', 'Original Signal');
```

## Inputs

|  |  |
| --- | --- |
| `signal` | input signal |
| `snr` | desired signal snr (signal to noise ratio) in decibels after adding noise |

## Optional Inputs

|  |  |
| --- | --- |
| `mode` | `'rms'` (default) or `'peak'` |

## Outputs

|  |  |
| --- | --- |
| `signal` | signal with added noise |
| `snr` | snr of output signal |

## Examples

- 2D Time Reversal For A Circular Sensor
- Attenuation Compensation Using Time Reversal
- Attenuation Compensation Using Time Variant Filtering

## See Also

`randn`
