# Supplementary material for: Asynchronous magnetic resonance elastography: Shear wave speed reconstruction using noise correlation of incoherent waves
Source: Magn Reson Med. 2022 Oct 27;89(3):990–1001. doi: 10.1002/mrm.29502 (PMC9792433; doi:10.1002/mrm.29502)
Supplement: Supplementary file 1 — DATA S1 MATLAB code used to generate the numerical simulations of Figure 1 [file MRM-89-990-s002.zip › k-Wave/helpfiles/acousticFieldPropagator.html]

acousticFieldPropagator :: Functions (k-Wave)


# acousticFieldPropagator

Calculate acoustic field for CW source.

## Syntax

```
pressure = acousticFieldPropagator(amp_in, phase_in, dx, f0, c0)
pressure = acousticFieldPropagator(amp_in, phase_in, dx, f0, c0, ...)
[amp_out, phase_out] = acousticFieldPropagator(amp_in, phase_in, dx, f0, c0)
[amp_out, phase_out] = acousticFieldPropagator(amp_in, phase_in, dx, f0, c0, ...)
[pressure, amp_out, phase_out] = acousticFieldPropagator(amp_in, phase_in, dx, f0, c0)
[pressure, amp_out, phase_out] = acousticFieldPropagator(amp_in, phase_in, dx, f0, c0, ...)
```

## Description

`acousticFieldPropagator` calculates the steady state field pattern (complex pressure or amplitude and phase) from an arbitrary phased array (or other acoustic source) driven by a single frequency continuous wave sinusoid in a homogeneous and lossless medium. The phased array is defined in completely general form as a matrix of amplitude and phase. This allows arrays of arbitrary geometry and numbers of elements to be defined.

The solution is based on the Green's function for the homogeneous wave equation expressed in the spatial frequency domain or k-space as outlined in [1]. The temporal convolution integral is solved analytically, and the remaining integrals are expressed in the form of the spatial Fourier transform. This allows the acoustic pressure for all spatial positions at any time t > 0 to be calculated in a single step without numerical quadrature. To avoid wave wrapping, the domain size used for calculation is automatically expanded to a suitable dimension size with small prime factors.

A simple example of computing the field from a line source in 2D is given below.

```
% define grid properties
Nx = 128; 		% [grid points]
Ny = 64;		% [grid points]
dx = 1e-4;		% [m]

% define input amplitude [Pa] and phase [rad] for a line source
amp_in = zeros(Nx, Ny);
amp_in(1, Ny/4:3*Ny/4) = 1;
phase_in = 0;

% define medium and source properties
f0 = 2e6;		% [Hz]
c0 = 1500;		% [m/s]

% compute pressure field
[amp_out, phase_out] = acousticFieldPropagator(amp_in, phase_in, dx, f0, c0);

% create plot axis
x_vec = 1e3 * (0:Nx-1) * dx;
y_vec = 1e3 * (-Ny/2:Ny/2 - 1) * dx;

% plot amplitude
figure;
subplot(1, 2, 1);
imagesc(y_vec, x_vec, amp_out);
axis image;
xlabel('Lateral Distance [mm]');
ylabel('Axial Distance [mm]');
title('Phase');

% plot phase
subplot(1, 2, 2);
imagesc(y_vec, x_vec, phase_out);
axis image;
xlabel('Lateral Distance [mm]');
ylabel('Axial Distance [mm]');
title('Phase');
```

[1] Treeby, B. E., Budisky, J., Wise, E. S., Jaros, J., & Cox, B. T. (2018). Rapid calculation of acoustic fields from arbitrary continuous-wave sources. *The Journal of the Acoustical Society of America*, 143(1), 529-537.

## Inputs

|  |  |
| --- | --- |
| `amp_in` | matrix of the source amplitude at each grid point [Pa] |
| `phase_in` | matrix of the source phase at each grid point [rad] |
| `dx` | grid spacing (assumed to be isotropic) [m] |
| `f0` | source frequency [Hz] |
| `c0` | medium sound speed [m/s] |

## Optional Inputs

Optional 'string', value pairs that may be used to modify the default computational settings.

| Input | Valid Settings | Default | Description |
| --- | --- | --- | --- |
| `'ExpandedGridSize'` | *(one, two, or three element vector)* or `'auto'` | `'auto'` | Option to specify the size of the grid after expansion used to avoid wave wrapping. |
| `'GridExpansionFactor'` | *(numeric scalar)* | `1.1` | Option to specify the multiplicative factor used to calculate the minimum expanded grid size to avoid wave wrapping based on time t. Note, setting a value for the optional input `'ExpandedGridSize'` will override this value. |
| `'GridSearchRange'` | *(integer)* | `50` | Option to set the search range used to find the expanded grid size with the smallest prime factors. Note, setting a value for the optional input `'ExpandedGridSize'` will override this value. |
| `'SaveToDisk'` | *(string)* | `''` | String containing a filename (including pathname if required). If set, after the precomputation phase, the input variables used in the time loop are saved the specified location in HDF5 format. The simulation then exits. The saved variables can be used to run simulations using the C++ code. |
| `'Time'` | *(numeric scalar)* or `'auto'` | `'auto'` | Option to specify the time t at which the wavefield is calculated to extract the amplitude and phase. |
| `'TimeExpansionFactor'` | *(numeric scalar)* | `1.5` | Option to specify the multiplicative factor used to calculate t based on the time taken to propagate across the longest grid diagonal. Note, setting a value for the optional input `'Time'` will override this value. |
| `'UseRamp'` | *(Boolean scalar)* | `true` | Option to use a smooth ramp to avoid start-up transients. |

## Outputs

|  |  |
| --- | --- |
| `pressure` | matrix of the complex pressure field at each grid point in steady state, where the real part corresponds to the pressure field for a cosine excitation, and the imaginary part corresponds to the pressure field for a sine excitation [Pa] |
| `amp_out` | matrix of the output amplitude at each grid point in steady state [Pa] |
| `phase_out` | matrix of the output phase at each grid point in steady state [rad] |

## See Also

`acousticFieldPropagatorC`, `kspaceFirstOrder1D`, `kspaceFirstOrder2D`, `kspaceFirstOrder3D`
