# Supplementary material for: Asynchronous magnetic resonance elastography: Shear wave speed reconstruction using noise correlation of incoherent waves
Source: Magn Reson Med. 2022 Oct 27;89(3):990–1001. doi: 10.1002/mrm.29502 (PMC9792433; doi:10.1002/mrm.29502)
Supplement: Supplementary file 1 — DATA S1 MATLAB code used to generate the numerical simulations of Figure 1 [file MRM-89-990-s002.zip › k-Wave/helpfiles/angularSpectrumCW.html]

angularSpectrumCW :: Functions (k-Wave)


# angularSpectrumCW

Project CW input plane using the angular spectrum method.

## Syntax

```
pressure = angularSpectrumCW(input_plane, dx, z_pos, f0, c0)
pressure = angularSpectrumCW(input_plane, dx, z_pos, f0, c0, ...)
pressure = angularSpectrumCW(input_plane, dx, z_pos, f0, medium)
pressure = angularSpectrumCW(input_plane, dx, z_pos, f0, medium, ...)
```

## Description

`angularSpectrumCW` projects an input plane of single-frequency continuous wave data (given as a 2D matrix of complex pressure values) to the parallel plane or planes specified by `z_pos` using the angular spectrum method. The implementation follows the spectral propagator with angular restriction described in reference [1].

For linear projections in a lossless medium, just the sound speed can be specified. For projections in a lossy medium, the parameters are given as fields to the input structure `medium`.

To compute the pressure field over an isotropic domain with `Nz` grid points (assuming the source plane is aligned with `z_ind = 1`), use the syntax:

```
pressure = angularSpectrumCW(input_plane, dx, (0:(Nz - 1)) * dx, f0, c0);
```

[1] Zeng, X., & McGough, R. J. (2008). Evaluation of the angular spectrum approach for simulations of near-field pressures. The Journal of the Acoustical Society of America, 123(1), 68-76.

## Inputs

|  |  |
| --- | --- |
| `input_plane` | 2D matrix of complex pressure values over a plane [Pa]. |
| `dx` | Spatial step between grid points in the input plane [m]. |
| `z_pos` | Vector specifying the relative z-position of the planes to which the data is projected [m]. |
| `f0` | Source frequency [Hz]. |
|  |  |
| `c0` | Medium sound speed [m/s]. |
|  | OR |
| `medium.sound_speed` | Medium sound speed [m/s]. |
| `medium.alpha_power` | Power law absorption exponent. |
| `medium.alpha_coeff` | Power law absorption coefficient [dB/(MHz^y cm)]. |

## Optional Inputs

Optional 'string', value pairs that may be used to modify the default computational settings.

| Input | Valid Settings | Default | Description |
| --- | --- | --- | --- |
| `'AngularRestriction'` | *(Boolean scalar)* | `true` | Boolean controlling whether angular restriction is used as described in [1]. |
| `'DataCast'` | *(string of data type)* | `'off'` | String input of the data type that variables are cast to before computation. For example, setting to `'single'` will speed up the computation time (due to the improved efficiency of `fft2` and `ifft2` for this data type). This variable is also useful for utilising GPU parallelisation the Parallel Computing Toolbox by setting `'DataCast'` to `'gpuArray-single'`. |
| `'DataRecast'` | *(Boolean scalar)* | `false` | Boolean controlling whether the output data is cast back to double precision. If set to `false`, `sensor_data` will be returned in the data format set using the `'DataCast'` option. |
| `'FFTLength'` | *(integer numeric scalar)* | 1 + the next power of two larger than the grid size | Length of the FFT used to compute the angular spectrum. |
| `'GridExpansion'` | *(integer numeric scalar)* | `0` | Grid padding used to increase the accuracy of the projection. The grid expansion is removed before returning the calculated pressure to the user. |
| `'Reverse'` | *(Boolean scalar)* | `false` | Boolean controlling whether the projection is in the forward (`false`) or backward (`true`) direction. |

## Outputs

|  |  |
| --- | --- |
| `pressure` | 3D matrix of complex pressure values across the 2D planes specified by `z_pos`, indexed as `(x_ind, y_ind, plane_index)` [Pa]. |

## See Also

`angularSpectrum`
