# Supplementary material for: Asynchronous magnetic resonance elastography: Shear wave speed reconstruction using noise correlation of incoherent waves
Source: Magn Reson Med. 2022 Oct 27;89(3):990–1001. doi: 10.1002/mrm.29502 (PMC9792433; doi:10.1002/mrm.29502)
Supplement: Supplementary file 1 — DATA S1 MATLAB code used to generate the numerical simulations of Figure 1 [file MRM-89-990-s002.zip › k-Wave/helpfiles/angularSpectrum.html]

angularSpectrum :: Functions (k-Wave)


# angularSpectrum

Project time-domain input plane using the angular spectrum method.

## Syntax

```
pressure_max = angularSpectrum(input_plane, dx, dt, z_pos, c0)
pressure_max = angularSpectrum(input_plane, dx, dt, z_pos, medium, ...)
[pressure_max, pressure_time] = angularSpectrum(input_plane, dx, dt, z_pos, c0)
[pressure_max, pressure_time] = angularSpectrum(input_plane, dx, dt, z_pos, medium, ...)
```

## Description

`angularSpectrum` projects a 2D input plane (given as a 3D matrix of time series at each spatial position) using the angular spectrum method. The time series are decomposed into spectral components and then each frequency is propagated using the spectral propagator with angular restriction described in reference [1].

The time signals are computed in retarded time, where the time series for each plane are offset by z/c0 and the input plane corresponds to z = 0. The projections are calculated directly from the input plane (no spatial stepping is used). For linear projections in a lossless medium, just the sound speed can be specified. For projections in a lossy medium, the parameters are given as fields to the input structure `medium`.

Two datasets are returned. The first, `pressure_max`, contains a 3D matrix of the maximum (temporal peak) pressure across the 2D planes specified by `z_pos`, and is indexed as `(x_ind, y_ind, plane_index)`. The second, `pressure_time`, contains the time-varying pressure signals across the 2D planes specified by `z_pos`, and is indexed as `(x_ind, y_ind, t_ind, plane_index)`.

To compute the maximum pressure field over an isotropic domain with `Nz` grid points (assuming the source plane is aligned with `z_ind = 1`) without storing the time-series (which can be memory consuming), use the syntax:

```
pressure_max = angularSpectrum(input_plane, dx, dt, (0:(Nz - 1)) * dx, c0)
```

To compute the time series over another parallel plane separated by a distance of `z_pos`, use the syntax:

```
[~, pressure_time] = angularSpectrum(input_plane, dx, dt, z_pos, c0)
```

[1] Zeng, X., & McGough, R. J. (2008). Evaluation of the angular spectrum approach for simulations of near-field pressures. The Journal of the Acoustical Society of America, 123(1), 68-76.

## Inputs

|  |  |
| --- | --- |
| `input_plane` | 3D matrix containing the time varying pressure over a 2D input plane indexed as (x, y, t) [Pa]. |
| `dx` | Spatial step between grid points in the input plane [m]. |
| `dt` | Temporal step between time points in the input plane [s]. |
| `z_pos` | Vector specifying the relative z-position of the planes to which the data is projected [m]. |
|  |  |
| `c0` | Medium sound speed [m/s]. |
|  | OR |
| `medium.sound_speed` | Medium sound speed [m/s]. |
| `medium.alpha_power` | Power law absorption exponent. |
| `medium.alpha_coeff` | Power law absorption coefficient [dB/(MHz^y cm)]. |

## Optional Inputs

Optional 'string', value pairs that may be used to modify the default computational settings.

| Input | Valid Settings | Default | Description |
| --- | --- | --- | --- |
| `'AngularRestriction'` | *(Boolean scalar)* | `true` | Boolean controlling whether angular restriction is used as described in [1]. |
| `'DataCast'` | *(string of data type)* | `'off'` | String input of the data type that variables are cast to before computation. For example, setting to `'single'` will speed up the computation time (due to the improved efficiency of `fft2` and `ifft2` for this data type). This variable is also useful for utilising GPU parallelisation the Parallel Computing Toolbox by setting `'DataCast'` to `'gpuArray-single'`. |
| `'DataRecast'` | *(Boolean scalar)* | `false` | Boolean controlling whether the output data is cast back to double precision. If set to `false`, `sensor_data` will be returned in the data format set using the `'DataCast'` option. |
| `'FFTLength'` | *(integer numeric scalar)* | 1 + the next power of two larger than the grid size | Length of the FFT used to compute the angular spectrum. |
| `'GridExpansion'` | *(integer numeric scalar)* | `0` | Grid padding used to increase the accuracy of the projection. The grid expansion is removed before returning the calculated pressure to the user. |
| `'Plot'` | *(Boolean scalar)* | `true` | Boolean controlling whether the field is plotted at each z step. |
| `'Reverse'` | *(Boolean scalar)* | `false` | Boolean controlling whether the projection is in the forward (`false`) or backward (`true`) direction. |

## Outputs

|  |  |
| --- | --- |
| `pressure_max` | 3D matrix of maximum pressure (temporal peak) across the 2D planes specified by `z_pos`, indexed as `(x_ind, y_ind, plane_index)` [Pa]. |
| `pressure_time` | 4D matrix of the time varying pressure signals across the 2D planes specified by `z_pos`, indexed as `(x_ind, y_ind, t_ind, plane_index)` [Pa]. |

## Examples

- Holographic Projections Using The Angular Spectrum Method

## See Also

`angularSpectrumCW`
