# Supplementary material for: Asynchronous magnetic resonance elastography: Shear wave speed reconstruction using noise correlation of incoherent waves
Source: Magn Reson Med. 2022 Oct 27;89(3):990–1001. doi: 10.1002/mrm.29502 (PMC9792433; doi:10.1002/mrm.29502)
Supplement: Supplementary file 1 — DATA S1 MATLAB code used to generate the numerical simulations of Figure 1 [file MRM-89-990-s002.zip › k-Wave/helpfiles/acousticFieldPropagatorC.html]

acousticFieldPropagatorC :: Functions (k-Wave)


# acousticFieldPropagatorC

Calculate acoustic field for CW source using C++ code.

## Syntax

See `acousticFieldPropagator`.

## Description

`acousticFieldPropagatorC` provides an interface to the C++ version of `acousticFieldPropagator` (called `acousticFieldPropagator-OMP`). The function works by appending the optional input `'SaveToDisk'` to the user inputs and then calling `acousticFieldPropagator` to save the input files to disk. The C++ code is run using the system command. The output files are then automatically loaded from disk and returned in the same fashion as `acousticFieldPropagator`. The input and output files are saved to the temporary directory native to the operating system, and are deleted after the function runs.

This function requires the C++ binary/executable of `acousticFieldPropagator-OMP` to be downloaded from http://www.k-wave.org/download.php and placed in the `"binaries"` directory of the k-Wave toolbox. Alternatively, the name and location of the binary can be specified using the optional input parameters `'BinaryName'` and `'BinariesPath'`.

## Optional Inputs

Optional 'string', value pairs that may be used to modify the default computational settings.

| Input | Valid Settings | Default | Description |
| --- | --- | --- | --- |
| `'BinaryName'` | *(string)* | `'acousticFieldPropagator-OMP'` on linux and `'acousticFieldPropagator-OMP.exe'` on windows | Name of the binary file. |
| `'BinaryPath'` | *(string)* | `'binaries/'` | Path of the binary file. |
| `'DataName'` | *(string)* | `'kwave_[input/output]_data_[date].h5'` | Prefix used to generate a custom name for the input and output data files (this is appended with `'_input.h5' and '_output.h5').` |
| `'DataPath'` | *(string)* | path returned by `tempdir` | Location of the folder where the input and output HDF5 files should be stored. |
| `'DeleteData'` | *(Boolean scalar)* | `true` | Boolean controlling whether the input and output HDF5 files should be deleted after running the simulation. |

## See Also

`acousticFieldPropagator`
