# Supplementary material for: Asynchronous magnetic resonance elastography: Shear wave speed reconstruction using noise correlation of incoherent waves
Source: Magn Reson Med. 2022 Oct 27;89(3):990–1001. doi: 10.1002/mrm.29502 (PMC9792433; doi:10.1002/mrm.29502)
Supplement: Supplementary file 1 — DATA S1 MATLAB code used to generate the numerical simulations of Figure 1 [file MRM-89-990-s002.zip › k-Wave/helpfiles/applyFilter.html]

applyFilter :: Functions (k-Wave)


# applyFilter

Filter input with high or low pass filter.

## Syntax

```
filtered_signal = applyFilter(signal, Fs, cutoff_f, filter_type)
filtered_signal = applyFilter(signal, Fs, cutoff_f, filter_type, ...)
```

## Description

`applyFilter` filters an input signal using `filter`. The FIR filter coefficients are based on a Kaiser window with the specified cut-off frequency and filter type (`'HighPass'`, `'LowPass'` or `'BandPass'`). Both causal and zero phase filters can be applied. An example of using `applyFilter` is given below.

```
% create a time series with a single monopolar pulse
N = 100;
dt = 1e-3;
t = 0:dt:dt * (N-1);
pulse = zeros(length(t), 1);
pulse(25) = 1;
    
% filter using applyFilter
pulse_filtered_lp = applyFilter(pulse, 1/dt, 100, 'LowPass', 'Plot', true, 'ZeroPhase', true);
pulse_filtered_hp = applyFilter(pulse, 1/dt, 100, 'HighPass', 'Plot', true);
pulse_filtered_bp = applyFilter(pulse, 1/dt, [50, 250], 'BandPass', 'Plot', true, 'ZeroPhase', true);

% plot the filtered time series
figure;
[~, scale, prefix] = scaleSI(max(t));
plot(t * scale, pulse, 'k-', ...
     t * scale, pulse_filtered_lp, 'r-', ...
     t * scale, pulse_filtered_hp, 'b-', ...
     t * scale, pulse_filtered_bp, 'g-');
xlabel(['Time [' prefix 's]']);
ylabel('Signal Amplitude [au]');
legend('Original Signal', 'Zero Phase Low Pass Filter', 'Causal High Pass Filter', 'Zero Phase Band Pass Filter', 'Location', 'best');
```


## Inputs

|  |  |
| --- | --- |
| `func` | signal to filter |
| `Fs` | sampling frequency [Hz] |
| `cutoff_f` | filter cutoff frequency/s [Hz] |
| `filter_type` | `'HighPass'`, `'LowPass'` or `'BandPass'` |

## Optional Inputs

Optional 'string', value pairs that may be used to modify the default computational settings.

| Input | Valid Settings | Default | Description |
| --- | --- | --- | --- |
| `'Plot'` | *(Boolean scalar)* | `false` | Boolean controlling whether the amplitude spectrum is displayed before and after filtering. |
| `'StopBandAtten'` | *(numeric scalar)* | `60` | Attenuation in decibels in the stop band. |
| `'TransitionWidth'` | *(numeric scalar)* | `0.1` | Size of the transition based on the temporal sampling frequency. |
| `'ZeroPhase'` | *(Boolean scalar)* | `false` | Boolean controlling whether a zero phase filter is used. |

## Outputs

|  |  |
| --- | --- |
| `filtered_signal` | the filtered signal |

## Examples

- Image Reconstruction With Bandlimited Sensors

## See Also

`filter`, `filterTimeSeries`
