# Supplementary material for: Asynchronous magnetic resonance elastography: Shear wave speed reconstruction using noise correlation of incoherent waves
Source: Magn Reson Med. 2022 Oct 27;89(3):990–1001. doi: 10.1002/mrm.29502 (PMC9792433; doi:10.1002/mrm.29502)
Supplement: Supplementary file 1 — DATA S1 MATLAB code used to generate the numerical simulations of Figure 1 [file MRM-89-990-s002.zip › k-Wave/helpfiles/beamPlot.html]

beamPlot :: Functions (k-Wave)


# beamPlot

Plot volumetric data using intersecting planes.

## Syntax

```
beamPlot(mat)
beamPlot(mat, plot_yz)
beamPlot(xy_slice, xz_slice)
```

## Description

`beamPlot` plots 3D volumetric data as intersecting planes using
`slice`. The data is assumed to be indexed as (x, y, z). The central x-y and x-z planes are extracted and plotted as intersecting planes. The first y-z plane can also be plotted by setting the optional input `plot_yz` to true. This orientation is useful for plotting the beam patterns produced by ultrasound transducers facing in the x-direction.

`beamPlot` can alternatively be called with two 2D slices which are plotted as intersecting planes indexed as (x, y) and (x, z). The slices must have the same size in the x-direction.

Examples:

```
beamPlot(makeBall(30, 30, 30, 15, 15, 15, 12));
beamPlot(makeDisc(40, 30, 20, 15, 10), makeDisc(40, 20, 20, 10, 5));
```

## Inputs

|  |  |
| --- | --- |
| `mat` | 3D matrix to plot |
| `plot_yz` | Boolean controlling whether the first y-z plane is displayed |
| `xy_slice` | slice to plot in the x-y plane |
| `xz_slice` | slice to plot in the x-z plane |

## See Also

`slice`
