# Supplementary material for: Asynchronous magnetic resonance elastography: Shear wave speed reconstruction using noise correlation of incoherent waves
Source: Magn Reson Med. 2022 Oct 27;89(3):990–1001. doi: 10.1002/mrm.29502 (PMC9792433; doi:10.1002/mrm.29502)
Supplement: Supplementary file 1 — DATA S1 MATLAB code used to generate the numerical simulations of Figure 1 [file MRM-89-990-s002.zip › k-Wave/helpfiles/attenComp.html]

attenComp :: Functions (k-Wave)


# attenComp

Attenuation compensation using time-variant filtering.

## Syntax

```
signal = attenComp(signal, dt, c, alpha_0, y)
signal = attenComp(signal, dt, c, alpha_0, y, ...)
[signal, tfd, cutoff_freq] = attenComp(signal, dt, c, alpha_0, y)
[signal, tfd, cutoff_freq] = attenComp(signal, dt, c, alpha_0, y, ...)
```

## Description

`attenComp` corrects for frequency dependent acoustic attenuation in photoacoustic signals using time-variant filtering [1]. The time-variant filter is constructed to correct for acoustic attenuation and dispersion following a frequency power law of the form `alpha_0*f^y` under the assumption the distribution of attenuation parameters is homogeneous. The filter is applied directly to the recorded time-domain signals using a form of non-stationary convolution. The approach is computationally efficient and can be used with any detector geometry or reconstruction algorithm.

To prevent high-frequency noise from being amplified, the compensation is regularised using a Tukey window with a time-variant cutoff frequency. The cutoff frequency can be specified manually using the optional input `'FilterCutoff'`. This is set as a two-element vector corresponding to the cutoff frequency in Hz for the first and last time points, respectively. For a fixed cutoff, these should be specified as the same value, e.g., `[3e6, 3e6]`. Alternatively, if `'FilterCutoff'` is set to `'auto'` (the default), the cutoff frequency is chosen based on the local time-frequency distribution of the recorded signals using the following steps:

1. Compute the average time-frequency distribution of the input signals (the method can be defined using the optional input `'Distribution'`)
2. Threshold the time-frequency distribution to remove noise (the threshold value can be defined using the optional input `'NoiseThreshold'`)
3. Calculate the integral of the thresholded time-frequency distribution at each time point using `cumsum`
4. Find the cutoff frequency at each time point where the integral reaches a given percentage of the maximum value (this percentage can be defined using the optional input `'EnergyThreshold'`)
5. Increase the filter cutoff frequency by a fixed multiplier so the cutoff corresponds to the edge of the passband for the Tukey window (the multiplier can be defined using the optional input `'FrequencyMultiplier'`)
6. Smooth the variation of the cutoff frequency over time (the smoothing function can be defined using the optional input `'FitType'`)
7. Threshold any values of the cutoff frequency below zero or above the Nyquist limit

If the input contains a matrix of signals, the cutoff frequency is based on the average time frequency distribution. To calculate the cutoff frequency for each signal individually, this function should be called in a loop. This can be parallelised, for example, using `parfor` from the parallel computing toolbox. For further details about this function and attenuation compensation using time variant filtering, see the reference below.

[1] B. E. Treeby (2013) "Acoustic attenuation compensation in photoacoustic tomography using time-variant filtering," J. Biomed. Opt., vol. 18, no. 3, p.036008.

## Inputs

|  |  |
| --- | --- |
| `signal` | matrix of time series to compensate indexed as `(sensor_index, time_index)` |
| `dt` | time step [s] |
| `c` | sound speed [m/s] |
| `alpha_0` | power law absorption prefactor [dB/(MHz^y cm)] |
| `y` | power law absorption exponent [0 < y < 3, y ~= 1] |

## Optional Inputs

Optional 'string', value pairs that may be used to modify the default computational settings.

| Input | Valid Settings | Default | Description |
| --- | --- | --- | --- |
| `'DisplayUpdates'` | *(Boolean scalar)* | `true` | Boolean controlling whether command line updates and compute time are printed to the command line. |
| `'Distribution'` | `'Rihaczek'` `'Wigner'` | `'Rihaczek'` | Time-frequency distribution used to automatically compute the filter cutoff frequency if `'FilterCutoff'` is set to `'auto'`. Note, the option `'Wigner'` requires the Discrete TFD toolbox from http://tfd.sourceforge.net. |
| `'EnergyThreshold'` | *(numeric scalar)* | `0.98` | Threshold value given as a percentage of the total amplitude spectrum used to choose the filter cutoff frequency at each time point. |
| `'FilterCutoff'` | *(numeric two element vector)* or `'auto'` | `'auto'` | Option to manually define the cutoff frequencies for a linear variation in the filter cutoff instead of using an automatic search. |
| `'FitType'` | `'spline'` `'linear'` `'mav'` | `'spline'` | Fitting type used to smooth the filter cutoff frequency after an automatic search, where `'spline'` fits a smoothed spline, `'linear'` fits a linear line, and `'mav'` computes the moving average. |
| `'FrequencyMultiplier'` | *(numeric scalar)* | `2` | By default, the compensation is regularised using a Tukey window with a time-variant cutoff frequency. The default Tukey window has a taper ratio of 0.5, so the filter cutoff frequency found by the automatic search is increased by a frequency multiplier so that the filter cutoff frequency corresponds to the edge of the passband of the Tukey window. |
| `'NumSplines'` | *(numeric scalar)* | `40` | Number of spline segments used in the smoothing spline if `'FitType'` is set to `'spline'`. |
| `'NoiseThreshold'` | *(numeric scalar)* | `0.03` | Threshold value given as a percentage of the signal maximum used to threshold the TFD before the automatic search for the filter cutoff. |
| `'Plot'` | *(Boolean scalar)* | `false` | Boolean controlling whether a plot of the time frequency distribution and filter cutoff frequency are displayed. |
| `'PlotRange'` | *(numeric two element vector)* or `'auto'` `'full'` | `'auto'` | Option to manually set the plot range in the frequency axis when `'Plot'` is set to `true`. This can be manually specified, or set to `'auto'` (1.5 x the maximum filter cutoff frequency) or `'full'` (maximum supported frequency range). |
| `'TaperRatio'` | *(numeric scalar)* | `0.5` | Taper ratio used to construct the Tukey Windows. |
| `'T0'` | *(numeric scalar)* | `0` | Time index of T0 in the input signals. For photoacoustic imaging, T0 corresponds to the arrival of the excitation laser pulse at the sample. |

## Outputs

|  |  |
| --- | --- |
| `signal_comp` | time series after attenuation compensation |
| `tfd` | average time frequency distribution of the input signals |
| `cutoff_freq` | filter cutoff frequency for each time index |

## Examples

- Attenuation Compensation Using Time Variant Filtering
